# Structural modeling of the N-terminal signal–receiving domain of IκBα

**DOI:** 10.3389/fmolb.2015.00032

**Published:** 2015-06-23

**Authors:** Samira Yazdi, Serdar Durdagi, Michael Naumann, Matthias Stein

**Affiliations:** ^1^Molecular Simulations and Design Group, Max Planck Institute for Dynamics and Complex Technical SystemsMagdeburg, Germany; ^2^Medical Faculty, Institute of Experimental Internal Medicine, Otto von Guericke UniversityMagdeburg, Germany

**Keywords:** signal transduction, NF-κB, IκBα, secondary structure prediction, N-terminal extension, molecular dynamics simulation, protein-protein complex refinement, signal receiving domain

## Abstract

The transcription factor nuclear factor-κB (NF-κB) exerts essential roles in many biological processes including cell growth, apoptosis and innate and adaptive immunity. The NF-κB inhibitor (IκBα) retains NF-κB in the cytoplasm and thus inhibits nuclear localization of NF-κB and its association with DNA. Recent protein crystal structures of the C-terminal part of IκBα in complex with NF-κB provided insights into the protein-protein interactions but could not reveal structural details about the N-terminal signal receiving domain (SRD). The SRD of IκBα contains a degron, formed following phosphorylation by IκB kinases (IKK). In current protein X-ray structures, however, the SRD is not resolved and assumed to be disordered. Here, we combined secondary structure annotation and domain threading followed by long molecular dynamics (MD) simulations and showed that the SRD possesses well-defined secondary structure elements. We show that the SRD contains 3 additional stable α-helices supplementing the six ARDs present in crystallized IκBα. The IκBα/NF-κB protein-protein complex remained intact and stable during the entire simulations. Also in solution, free IκBα retains its structural integrity. Differences in structural topology and dynamics were observed by comparing the structures of NF-κB free and NF-κB bound IκBα-complex. This study paves the way for investigating the signaling properties of the SRD in the IκBα degron. A detailed atomic scale understanding of molecular mechanism of NF-κB activation, regulation and the protein-protein interactions may assist to design and develop novel chronic inflammation modulators.

## Introduction

### NF-κB signaling and its inhibitor IκBα

NF-κB plays a crucial role in mediating responses to various types of external stimuli, thus it is a key element in multiple physiological and pathological processes (Oeckinghaus and Ghosh, 2009). Defective NF-κB activity may lead to very serious health problems such as cancer and chronic inflammatory diseases (i.e., arthritis and Crohn's disease; for reviews see Bouma and Strober, 2003; Schreiber et al., 2005; Viatour et al., 2005). The NF-κB protein is bound by IκBα in unstimulated cells, keeping it inactive and retaining it in the cytoplasm and thus inhibiting nuclear localization of NF-κB and its association with DNA. Since NF-κB binds to a specific DNA motif in the nucleus and regulates transcription of target genes, the inhibition of NF-κB can be a therapeutic target for the prevention or treatment of undesired biologic responses caused by the uncontrolled activation of NF-κB.

Upon activation, the IKKβ kinase phosphorylates IκBα at specific amino acid positions (i.e., Ser32 and Ser36) (Viatour et al., 2005). This site-specific phosphorylation of IκBα is a prerequisite for its ubiquitination by a specific E3 ubiquitin ligase the SKP1-CULLIN1-F-box (SCF) E3 ligase SCF(β-TrCP). SCF(β-TrCP)-mediated IκBα ubiquitination and degradation is very efficient and resulting in complete degradation of IκBα within a few seconds of cell stimulation (Suzuki et al., 1999; Neumann and Naumann, 2007).

**Figure 1:**
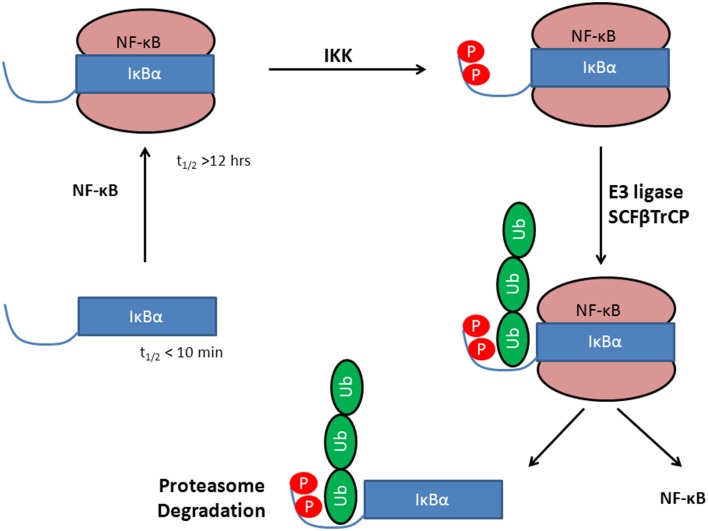
**Stabilization of IκBα upon protein-protein complex formation with the transcription factor NF-κB**. Phosphorylation by IKK leads to a degron, recognized by SCF(β-TrCP) and subsequent IκBα ubiquitination by E3. This activates NF-κB whereas IκBα is degraded in the 26S proteasome.

IκBα in complex with NF-κB is highly stable and has an intracellular half-life of several hours (Hatada et al., 1992; Jaffray et al., 1995), while the free IκBα has a half-life of less than 10 min (Jaffray et al., 1995; Sun et al., 1996) and all efforts to crystallize IκBα in its unbound state have been unsuccessful so far (Huxford et al., 1999). Once bound to NF-κB, IκBα is only degraded if it is first phosphorylated, then ubiquitinated, and finally degraded by the proteasome (see Figure 1).

**Figure 2:**
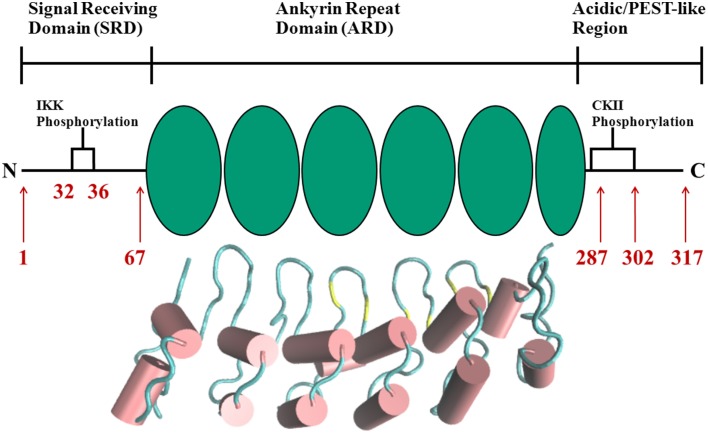
**Schematic representation of protein domains in IκBα**. The N-terminal SRD is the site of phosphorylation by IKK and subsequent ubiquitination by the SCF (β-TrCP) E3 ligase, six ankyrin repeat units make up the central ARD domain. The ankyrin repeat units in the protein crystal structure of IκBα in complex with NF-κB (pdb entry 1IKN) are shown as a cartoon representation. Indicated for the C-terminal PEST-like region are the CKII sites of phosphorylation. The diversity of binding sites, the great variability of κB-sites in the DNA motif and the existence of suppressive and inductive NF-κB dimers lead to a complexity and versatility of the downstream signaling network.

The complexity and versatility of the downstream signaling network is controlled, among others, by NF-κB-specific inhibitor proteins, namely IκBs (Schuster et al., 2013). IκBs are the critical regulators of NF-κB activity. They contain a signal receiving domain (SRD), six to seven ankryin repeat units (Dyson and Komives, 2012) and a largely unstructured PEST [enriched in amino acids proline (P), glutamate (E), serine (S) and threonine (T)] domain at the C-terminus (Figure 2). The C-terminal PEST domain is also the site of post-translational modifications due to the casein kinase II (CK2) phosphorylation at positions 283, 288, 291, 293, and 299 (Cuff et al., 1998; Palopoli et al., 2009).

A detailed understanding of molecular mechanism of NF-κB activation, regulation and the protein-protein communication with partners may assist to design and develop novel chronic inflammation modulators as well as anti-cancer drugs. The insight gained from structural biology of NF-κB and IκBα proteins and its implications for the signaling process control have been reviewed extensively by i.e., (Moorthy et al., 2006; Ferreiro and Komives, 2010; Huxford et al., 2011; Ghosh et al., 2012).

The SRD of IκBα is the central signal receiving and transmitting domain when activating NF-κB. It contains sites for post-translational modifications [phosphorylation by kinases IKKα and IKKβ (Huxford et al., 1998; Moorthy et al., 2006) at Ser32 and Ser36; and Lys21 and Lys22 as the sites for subsequent ubiquitination by SCF(β-TrCP), respectively (Jacobs and Harrison, 1998; Cervantes et al., 2009)]. The SRD was always assumed to be unstructured or highly disordered based on the failed attempts to crystallize full-length IκBα in complex with NF-κB. The instability of free IκBα in solution and the absence of significant SRD contributions to the interaction energy of the protein-protein complex of IκBα/NF-κB lead to the hypothesis of the SRD not being critical for this complex formation. Detailed knowledge of the NF-κB/IκBα interaction comes from protein crystallography (Huxford et al., 1998) and high resolution NMR experiments (Schuster et al., 2013). However, these results do not include any structural information about the SRD (residues 1–72) of IκBα. Previous investigations by molecular dynamics (MD) simulations of NF-κB/IκBα focused on the amide proton/deuterium exchange kinetics of four central ankyrin repeat units of co-crystallized IκBα by accelerated molecular dynamics (aMD) simulations (Cervantes et al., 2009), a truncated free-IκBα (Ferreiro et al., 2007) and the structure of a free, doubly phosphorylated 24 amino acid peptide of the SRD (Pons et al., 2007).

The concept of conservation of secondary structure elements (SSEs) (Rost, 2001) in families can be used to identify proteins only distantly related in sequence, which may, however, still share a higher degree of conservation of SSEs. Recent approaches have demonstrated that the use of multiple tools of secondary structure prediction and the use of a “consensus” of methods yields more reliable results than single algorithms (Cuff et al., 1998; Palopoli et al., 2009).

### Ankyrin repeat units as interaction modules domain

The crystal structure of IκBα illustrates how the six repeating ankyrin domain assumes the shape of an arched cylinder assembled on top of the interface of the NF-κB heterodimer. Every repeat unit in IκBα is composed of two α-helices connected to each following repeat with a loop of varying size and a β-hairpin turn containing short β-strands. However, repeats 1, 3, and 4 deviate from the canonical 33 amino acid repeat unit. These repeats are longer than the repeat units in the ankyrin consensus sequence, with the insertions contained in the loop sections, as these regions are those with the lowest sequence similarity among all ankyrin repeat proteins. Lack of homology is also observed in the sixth and last repeat unit, where the dissimilarity falls in after the second helix clearing the last 11 residues of any secondary structural elements (Huxford et al., 1998).

Free IκBα (67–317) was characterized by circular dichroism (CD) spectroscopy, 8-anilino-1-napthalenesulphonic acid (ANS) binding, differential scanning calorimetry (DSC), and amide hydrogen/deuterium exchange experiments (Croy et al., 2004). The CD spectrum of free IκBα is nearly identical to the CD spectrum of the IκBα/NF-κB complex but it shows significant ANS binding and rapid amide exchange over much of the protein. These findings suggest that the secondary structure of IκBα is formed but the tertiary structure may not be compact. The β-hairpins of AR2 and AR3 were remarkably resistant to exchange, whereas AR5 and AR6 exchanged completely within the first minute in free IκBα. When bound to NF-κB, the β-hairpins of AR5 and AR6 showed dramatically less exchange in the bound state (Truhlar et al., 2006).

### The SRD in IκBα protein crystal structures

From the structure of IκBα in complex with NF-κB, a valuable level of insight was rendered into the regulation of NF-κB by IκBα and the nature of their association (Huxford et al., 1998). Each unit of the complex was partially truncated leading to a missing IκBα N-terminal segment comprising ~70 residues. This N-terminal SRD receives the phosphorylation and ubiquitination signals and targets the protein to the proteasome for degradation (Traenckner and Baeuerle, 1995), but has no measureable effect on binding of IκBα to NF-κB (Huxford et al., 1999). While SRD plays a crucial part in activation of NF-κB, it has not been found to be engaged in enabling the complex formation of IκBα/NF-κB (Hatada et al., 1992; Jaffray et al., 1995; Sun et al., 1996). Protein crystallization and structure determination were unsuccessful for free IκBα due to its short lifetime and degradation within minutes. This led to the suggestion of conformational disorder in the free protein (Cervantes et al., 2009). For IκBα in complex with NF-κB, however, there are two protein crystal structures available (PDB IDs 1IKN and 1NFI). The truncated IκBα sequences in 1IKN (residues 73–292) (Huxford et al., 1998) and 1NFI (residues 71–280) (Jacobs and Harrison, 1998), however, did not reveal information about possible secondary structure elements in the SRD.

In this study, we present for the first time, the structural elements of the full length SRD of IκBα in complex with NF-κB and in free IκBα. We clearly show that the SRD displays well-defined secondary structure elements and cannot be considered as “unstructured.” In contrast, it contains three α-helical regions which are stable during molecular dynamics simulations. Also, in free IκBα the SRD is structured albeit displaying a larger degree of flexibility and larger fluctuations.

This represents the first step in an approach to model the signal transduction cascade of the NF-κB/IκBα complex from IKK phosphorylation to degradation.

## Materials and methods

### Structural modeling

The secondary structure prediction of the full IκBα sequence was performed with SYMPRED^1^ which builds upon results from PROF (Rost and Sander, 1994), SSPRO (Pollastri et al., 2002), YASPIN (Lin et al., 2005), and PSIPRED (Jones, 1999). In addition, JPRED3 (Leman et al., 2013), JUFO (Leman et al., 2013), NetSurfP (Petersen et al., 2009), PORTER (Pollastri and McLysaght, 2005), PredictProtein (Rost et al., 2004), and ScratchProteinPredictor (Cheng et al., 2005) were also used. All secondary structure prediction algorithms correctly identified and positioned the six ankyrin repeat units in the crystal structure (PDB ID 1IKN) in addition to four additional α-helical regions in the N-terminal SRD, which is not resolved in the protein crystal structure. The consensus of predicted secondary structure elements was used for structural modeling of the SRD.

To identify a suitable structural template for modeling the SRD of IκBα, we used pDomThreader (Lobley et al., 2009), a profile based recognition fold method incorporating domain superfamily discrimination, which distinguished 46 probable structural templates. We chose the fourth ranked template, 1N11, as a suitable template basing our decision on a top alignment score, the degree of coverage and the structural alignment of 286 out of 317 residues in IκBα. pDomTHREADER (Lobley et al., 2009) makes use of the CATH database of annotation of protein structural superfamilies from the PDB (Sillitoe et al., 2013). It is an implementation of GenTHREADER, a method which predicts protein fold from sequence by integrating profile-profile alignments, secondary structure gap penalties and both classic pair and solvation potentials employing an optimized regression SVM model. pDomTHREADER is thus able to discriminate between different structural superfamilies from the protein sequences and to detect distant homology to proteins of known structure.

The structural model was generated with Prime (Jacobson et al., 2002, 2004). A manually constructed sequence alignment of our templates 1IKN and 1N11 was used. A two-template composite model was thereby constructed; residues 73–292 were based on the crystallized IκBα protein 1IKN while the secondary structure elements of ankyrin protein 1N11 served as basis for residues 1–98. The Build process involves coordination of the copying of the backbone atoms for aligned regions and side chains of conserved residues, building insertions and deletions in the alignment, optimization of side chains not found in template and energy minimization of those residues not derived from the templates. The Prime Build process applies the OPLS_2005 all-atom force field for energy scoring and the Surface Generalized Born (SGB) continuum solvation model for treating solvation energies and effects. Additionally it utilizes the residue-specific side-chain rotamer and backbone dihedral libraries, derived from the non-redundant data sets extracted from the PDB.

### System assembly and protocol for MD simulations

The MD simulations were carried out using Gromacs 4.5 (van der Spoel et al., 2005; Pronk et al., 2013) employing the GROMOS96 43a1 force field (Scott et al., 1999). The all-atom structural model of IκBα bound to the X-ray crystallographic structure of NF-κB included 6945 atoms in total. The protein complex was immersed in a rectangular box of dimensions 78 × 89 × 145 Å^3^ solvated with 29686 SPC water molecules together with 117 Na^+^ and 91 Cl^−^ ions in order to neutralize the net system charge. The structural model of the free IκBα was immersed in a slightly smaller rectangular box of dimensions 66 × 68 × 114 Å^3^ solvated with 15757 SPC water molecules together with 70 Na^+^ and 47 Cl^−^ ions, in total containing 50270 atoms. The LINCS algorithm (Hess et al., 1997) was applied for constraining bond lengths. Electrostatic interactions were calculated every step with the Particle-Mesh Ewald algorithm (Essmann et al., 1995). Neighbor lists were saved and reused for five steps. The simulations were performed at constant pressure of 1.0 bar with Parrinello-Rahman pressure coupling and the isotropic pressure scaling, time constant of 1.0 ps, and a system compressibility of 4.5e-5 bar^−1^. The temperature of the system was coupled to 300 K using the velocity-rescaling algorithm with a time constant of 0.1 ps. Newton's equations of motion were integrated using the leap-frog algorithm with a 2 fs time step.

The solvated system was first minimized with the steepest descent algorithm until a maximum force of < 100.0 kJ/mol was reached. Equilibration of the system was initiated by 10000 steps of position-restrained MD by relaxing the solvent and keeping the non-hydrogen atoms of the system fixed. With the system relatively free of strain an NVT equilibration phase followed by an NPT phase of 10000 steps each was then carried out. Coordinates were saved every 2 ps for analysis and the production phase of the simulation ran for a total of 200 ns. Fan and Mark have shown that molecular dynamics simulation in explicit water are able to refine homology-based protein structures within a short period of simulation (Fan and Mark, 2004). For small to medium-sized proteins (50–100 amino acids), the first 1–5 ns were able to remove initial distortions and only in few cases simulations of > 100 ns were necessary to obtain a significant reduction of RMSD. We took this as a lower threshold and added a factor of two considering the complexity of the system. Three independent replicates of our system were simulated for 600 ns in total, each starting with different initial velocities. Simulating independent replicates is a rather cost-effective way to sample conformational space (Elofsson and Nilsson, 1993).

## Results and discussion

### Structural elements in the signal-receiving domain (SRD)

In order to better understand the effects of phosphorylation and the mechanisms, which govern recognition of phosphorylated IκBα, and consequentially initiate ubiquitination, one requires a structural model of the complete N-terminal protein SRD. A BLASTp search of the first 72 residues of IκBα did not yield any significant sequence similarity with other known proteins.

**Figure 3:**
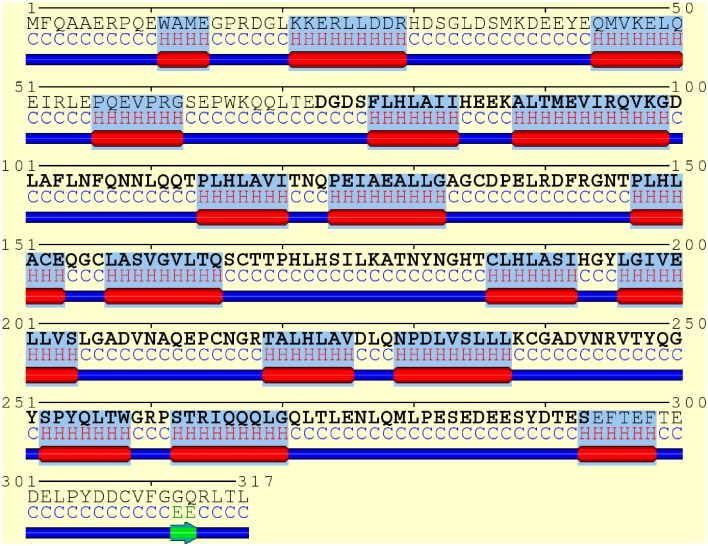
**Consensus secondary structure annotation of full length IκBα (residues 1–317)**. Truncated, as crystallized IκBα from 1IKN (residues 73–293) is shown in bold letters. The six ankyrin repeat units of the ARD are recovered, correctly annotated and positioned. Two additional α-helix-loop-α-helix regions were detected in the N-terminal SRD. The C-terminal PEST domain displays less structural features.

A consensus-based secondary structure annotation of the full length IκBα sequence with SYMPRED was performed which builds upon results from PROF (Rost and Sander, 1994), SSPRO (Pollastri et al., 2002), YASPIN (Lin et al., 2005), and PSIPRED (Jones, 1999) (see Figure 3).

In addition, JPRED3 (Leman et al., 2013), JUFO (Leman et al., 2013), NetSurfP (Petersen et al., 2009), PORTER (Pollastri and McLysaght, 2005), PredictProtein (Rost et al., 2004), and ScratchProteinPredictor (Cheng et al., 2005) were also employed and give close to identical results (see Figure 1 of the Supplementary Material).

All six ankyrin repeat units in the crystal structure (PDB ID: 1IKN (Huxford et al., 1998)) are recovered, correctly annotated and positioned. In addition four α-helical regions were detected in the N-terminal SRD, which is not resolved in the protein crystal structure (see Figure 3).

This indicates that the SRD region may contain secondary structured subregions with a high α-helical content (residues 11–14, 21–29, 44–50, 56–62) not covered in any of the available IκBα crystal structures and not investigated in any of the NMR studies of free or complexed IκBα. The position of these α-helices is not fixed with respect to each other and may obstruct protein crystallization of full length IκBα.

A detailed residue-based secondary structure prediction with confidence score can be found in the Supplementary Material, Figure 2. This initial finding prompted the generation of a full-length IκBα model including the SRD and the investigation of its spatial and temporal integrity and stability.

Due to the absence of sequence similarity of the SRD region to any structurally resolved protein in the PDB (~12%) sequence-based comparative modeling is not a feasible approach here. As an alternative, the choice of template was based on identification of a remote structurally related protein template with a similar secondary structure fold. The conservation of secondary structure elements (SSEs) in protein superfamilies can guide the design of a structural model. Even when the structure of only a single member of a superfamily is known the conservation of SSEs can be used to predict the structure of other superfamily members (Mizuguchi and Blundell, 2000; Geourjon et al., 2001). Such information is useful when modeling the structure of other members of a superfamily or identifying structurally and functionally important positions in the fold. An efficient template detection allows the structural modeling to be extended even in the twilight zone of 10–30% sequence identity (Geourjon et al., 2001).

pDomTHREADER (Lobley et al., 2009) identifies 46 possible structural templates with reliable secondary structural similarity. Based on a top alignment score, the degree of coverage and the structural alignment of 286 out of 317 residues in IκBα, we chose one of the top ranked structures (1N11) as a template for modeling IκBα (for a full list of templates see Supplementary Material, Figure 3).

As an alternative approach, a combination between comparative modeling and *de novo* protein structure prediction was performed using Robetta. For proteins with detected PDB homologs, comparative models are built based on templates that are found and aligned with incorporated versions of HHSEARCH/HHpred, RaptorX, and Sparks-X. Protein domains with no close PDB homologs are generated with the Rosetta *de novo* protocol (Simons et al., 1997; Bradley et al., 2005). A structure prediction carried out by Robetta (Kim et al., 2004) for the full IκBα sequence also yielded 1N11 as the top-ranked template of choice for the generation of its structural models.

**Figure 4:**
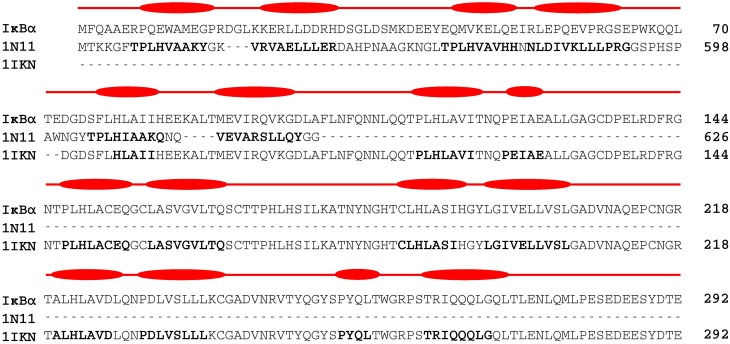
**Two-template sequence alignment used for the generation of a composite structural model of the full-sequence IκBα**. The bold segments in each template correspond to the α-helical regions forming the ankyrin repeat units present in the crystal structures of 1IKN and 1N11. The curved boxes in red display the helical segments in our generated structural model of IκBα.

Figure 4 shows the alignment of secondary structure elements of IκBα and 1N11 in the SRD region.

Despite an overall low primary sequence identity of only 23%, the alignment of secondary structural elements is striking. 1N11 is the crystal structure of a 12 ankyrin repeat units stack from the human ankyrinR. AnkyrinR belongs to a family of adaptor proteins that mediate anchoring between integral membrane proteins and the spectrin-actin cytoskeleton. The membrane-binding domain of ankyrins contains 24 ankyrin repeats of which the crystal structure of the human ankyrinR maps the D34 region. This region, which consists of repeats 13–24, is stacked contiguously in the shape of a left-handed superhelix (Michaely et al., 2002).

A composite model from crystallized IκBα (67–317) 1IKN and ankyrinR 1N11 PDB structures was generated. Residues at positions 73–292 were taken from the crystallized IκBα protein (PDB ID: 1IKN) and for residues 1–98 SSEs of the SRD were taken from the X-ray structure 1N11. For an overlapping stretch of residues 73–98, two α-helices forming one ankyrin repeat in the 1N11 template was taken to remove any possible artifacts from truncated sequence crystallization.

### Structural refinement by molecular dynamics simulations

The protein-protein complex model was used as a starting configuration for subsequent MD refinement. The stability of the suggested secondary structural elements in the SRD and the dynamics of possible rearrangements were investigated.

In order to achieve a reliable full-sequence structural model, we performed three independent MD simulations of IκBα in complex with NF-κB for 200 ns each in a neutralized solvent box of about 30000 explicit water molecules. Thus, a total production simulation time of 600 ns was achieved. After energy minimization, a stepwise relaxation of the simulation setup and careful equilibration first in an NVT and then in an NPT ensemble, the general behavior of all simulation runs reveals well-behaved and stable systems. This is reflected in the conservation of total energy and temperature of the entire system (Supplemental Material, Figure 4), which is kept at a constant room temperature of 300 K (Supplemental Material, Figure 5) throughout the whole 200 ns simulation runs.

The structural stability of the IκBα/NF-κB complex is also monitored by calculating the root mean square displacement (RMSD) from the starting protein-protein complex structure (Supplemental Material, Figure 6). The RMSD increases sharply to 3.5–4.5 Å for the three replicate runs during the first 100 ns of the simulations, and settles at roughly 4.5–5.5 Å for the last 100 ns, indicating a well-structured stable complex. The results discussed herewith are the average findings of the three replicate runs unless otherwise stated.

In order to investigate the secondary structural profile of our IκBα initial model and possible structural re-arrangements, we have sectioned our 200 ns simulation into two equal parts. This provides a comparison of results at the beginning and end of the production run periods.

**Figure 5:**
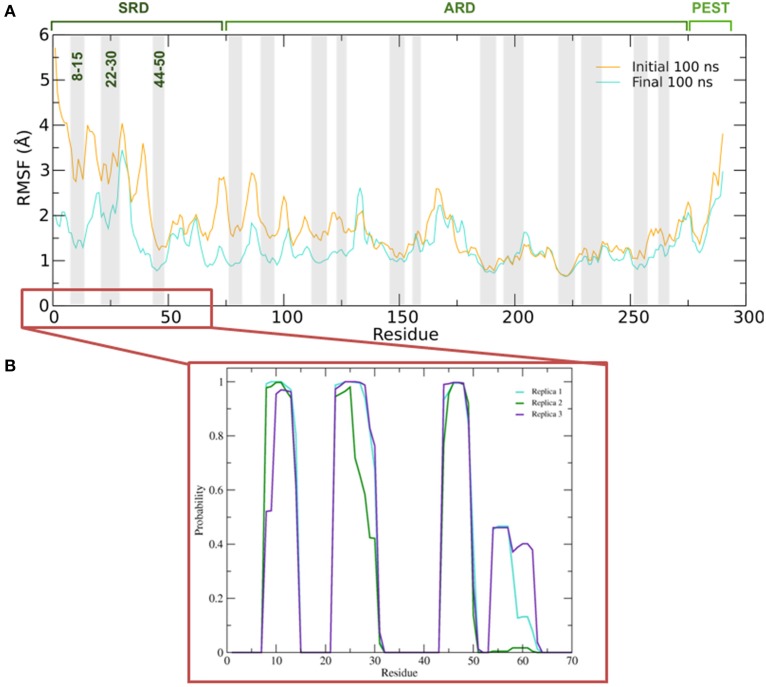
**(A)** Average root mean square fluctuations (RMSF) of the backbone of IκBα for the initial and final 100 ns of the simulation. Shaded areas depict α-helical regions at the end of the three independent 200 ns simulation periods. **(B)** Probability distribution of α-helix formation of the first 70 residues of the SRD of IκBα in complex with NF-κB.

To better understand the inherent flexibility of our protein, the root mean square fluctuations (RMSF) of the backbone Cα atoms of IκBα around the average structure were calculated (Figure 5A). The SRD N-terminal segment comprising ~75 residues clearly stands out as the most flexible region, particularly in the initial 100 ns of the simulations. Although not as expressed as in the initial 100 ns of simulations, the flexibility in the subsequent 100 ns run region is still comparatively high. We encounter the two most flexible helical regions of the whole protein, namely helices one and two, also in this region. This result indeed explains the difficulty to crystallize the SRD region. Instead an N-terminal truncation of IκBα was necessary to obtain protein single crystals (Cervantes et al., 2009). We see, in general, the retention of all crystallographically resolved six ankyrin repeat units in IκBα during the entire simulation runs (Figure 5A). While the peaks mark the hairpin loop segments connecting the α-helices in each ankyrin repeat unit, the troughs of the RMSF plot correspond to helical regions. This result shows that while the helical regions are stable and not so flexible, greater flexibility is observed in the β-loop segments. This is in agreement with the amide ^1^H/^2^H exchange experiments followed by MALDI-TOF mass spectrometry (MS) in bound and free IκBα (Croy et al., 2004). The β-hairpins of some ankyrin repeats readily exchange amide protons for deuterons (1st, 5th, and 6th ankyrin units) whereas other units (Bouma and Strober, 2003; Schreiber et al., 2005; Viatour et al., 2005) are less solvent accessible. In particular, ankyrin repeat unit 1 remains highly solvent accessible even in the complex. The solvent accessibility of the β-hairpin in ankyrin repeat unit 1 (AR 1) decreases slightly upon NF-κB binding (Truhlar et al., 2006).

Figure 5B gives the probability distributions of helical formations in the SRD of IκBα. Together with the RMSF of Figure 5A, we obtain a consistent picture of stable vs. flexible subregions in the SRD.

**Figure 6:**
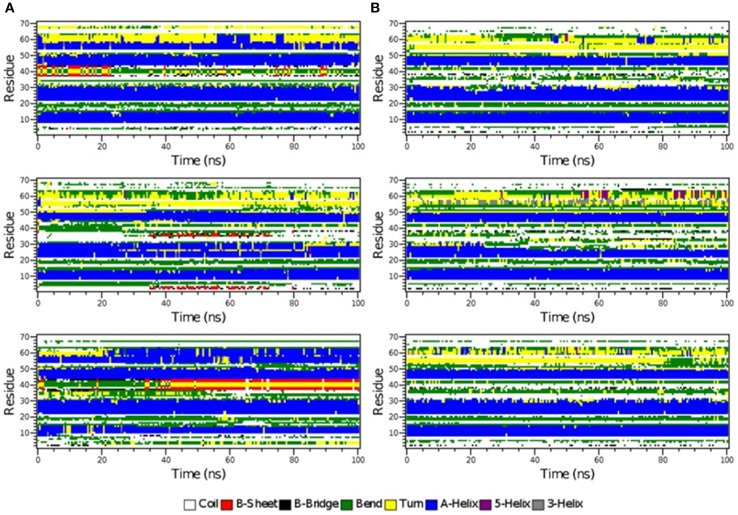
**The secondary structure elements of the first 70 N-terminal residues of IκBα in complex with NF-κB as calculated by DSSP for the three system replicas during the initial 100ns (A) and final 100 ns (B) of the simulation**.

Residues 31–37 in the SRD immediately adjacent to the second α-helix in the N-terminal region represent the most flexible part of IκBα, in the case of disregarding residues beyond 275 (Figure 5A). It is natural to discard residues beyond 275 from the comparison as they form a long loop and constitute a rather disordered region void of any tertiary structure. We do see the conservation of three α-helices, residues between 8–15, 22–30, and 44–50 within the SRD region. These values are in agreement with the predicted secondary structure models, which identified the three α-helices to lie between residues 10–13, 22–29, and 44–50. The last two α-helices align perfectly, while SYMPRED predicts a somewhat shorter α-helix compared to that observed in the refined structure. Furthermore, the fourth α-helical element, which was positioned from residues 54–63 from the 1N11 template, no longer adopts an α-helical shape but acquires instead a less ordered loop conformation (see Figures 5B, 6, below). Here, obviously our refinement by MD simulations is sufficient to remove the ambiguous assignment of secondary structure elements and provide a more stable conformation of this stretch of ten amino acid residues in length. All other secondary structure elements are retained during the MD simulations. This gives us confidence in the reliability of our protein-protein complex model and the existence of well-defined secondary structural elements in the SRD of IκBα when it is in complex with NF-κB.

The time-evolution of secondary structure elements in the N-terminal SRD during the MD refinement is then analyzed in detail. The DSSP-annotated SSEs of the first 70 amino acid residues in IκBα for each of the replica systems is plotted in order to analyze the SSEs of the first 70 amino acid residues during the MD trajectory frames (see Figure 6). The first three α-helices, residues between 8–15, 22–30, and 44–50 retain their α-helical structure (blue regions) during the initial 100 ns MD simulations in all three system replicas (Figure 6A). They are followed by a recurring β-sheet turn β-sheet formation (green-yellow-green). This region is followed by an unstable α-helix that is formed between residues 52–62. This short helix is observed only in two of the replicas (top and bottom). This segment mainly adopts the turn/bend secondary structure in the third replica. The structural stability is observed for the first three α-helices throughout the entire simulations during the final 100 ns of the simulation runs. (Figure 6B) The temporarily formed fourth α-helix, however, observed in the first 100 ns, is no longer formed and the sequence instead remains variable in its secondary structure. During most of the production runs, it takes a turn-like secondary structure (yellow) or bend (green) with short interludes of stretches of 3_10_-helices (gray) and π-helices (purple).

**Figure 7:**
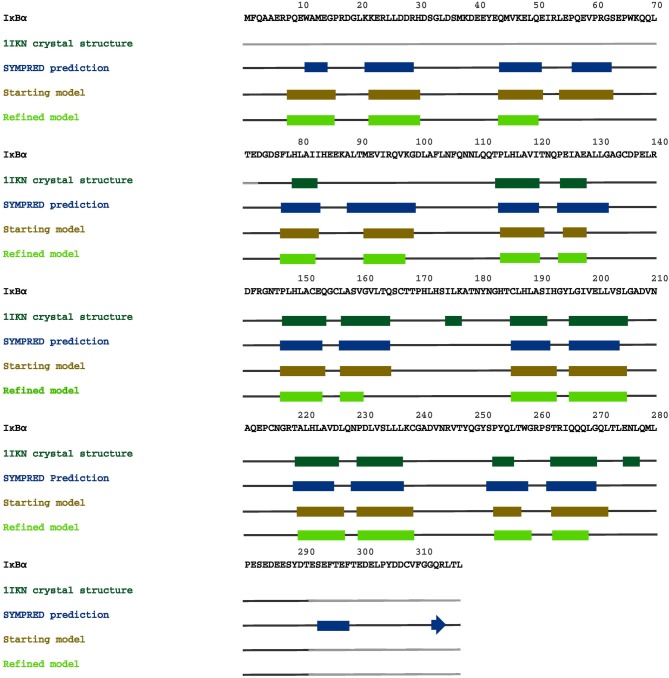
**A graphical map of the secondary structure elements of IκBα, displayed on its complete sequence**. The boxes highlight the α-helical regions, and the arrows indicate β-strands. Dark green designates secondary structures determined in the crystal structure 1IKN, blue denotes secondary structures predicted by SYMPRED, and brown and fluorescent green indicate secondary structures suggested by our initial and refined structural models.

In Figure 7, we summarize our results from secondary structure prediction, initial model generation and secondary structure elements of the full-length IκBα obtained after MD refinement. Four helical stretches were detected from consensus SSE prediction and thus also represented the starting SRD model (top line, up to residue 70). After MD refinement, three helical stretches are structurally retained and the fourth one was not stable and adopts a disordered conformation. The ankyrin repeats of the ARD are structurally stable during the MD simulations of the protein-protein complex and well-positioned with respect to the crystal structure.

**Figure 8:**
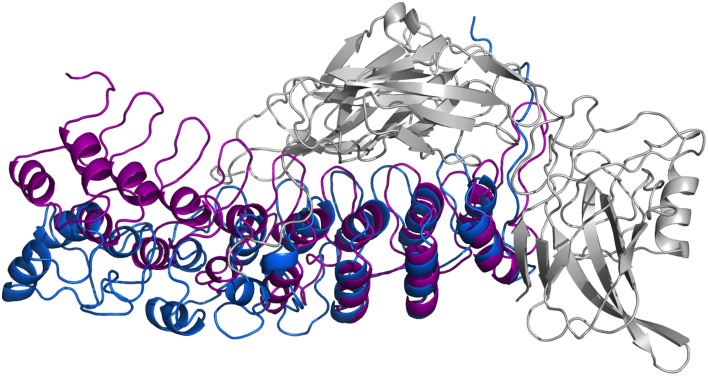
**Ribbon diagrams of the three-dimensional initial structure (purple) and the refined structure after a 200 ns MD simulation (blue) of IκBα**. The structures are shown in comparison by superpositioning IκBα's binding partner NF-κB (gray).

In Figure 8, we present our refined structural model of the IκBα-NF-κB complex (blue) portrayed together with the initial structural model (purple). The refined representative structure is depicting the last frame of a system replicate that has the lowest RMSD with respect to the average structure. This model reveals three helical structures in the previously not resolved SRD unit in addition to the six ankyrin repeats in the ANK protein domain. While the inner helix is nine residues long and extends from positions 22–30, the initial helix in the first pair of helices is eight residues long, spanning from positions 8–15 in the IκBα. The α-helix pair is followed by a 13-residue long loop, joining this element with the consecutive α-helix of seven residues long covering positions 44–50. The lengthy loop linking the third helix to the subsequent ankyrin repeat domain comprises 26 residues, and connects the unresolved N-terminal segment of IκBα to the crystallized ankyrin repeat domain of this protein. The structural superpositioning of the initial and refined models of IκBα bound to its partner, NF-κB, reveals an ANK domain that is partly rigid and well-structured. Ankyrin repeats 4–6 remained intact and display greater stability when bound to NF-κB, while ankyrin repeats 1–3 show increased flexibility. This is in agreement with the analysis of residual dipolar coupling (RDC) of free and bound IκBα which showed that helix two from ankyrin repeat 3 differed most in the free and bound forms (Cervantes et al., 2009).

In particular, ankyrin repeat 1 shows the greatest displacement, which together with the SRD segment move away from NF-κB and deviate the most from the initial structure. This is in agreement with experimental studies which could show that the SRD does not contribute to the overall NF-κB binding affinity to IκBα (Malek et al., 1998). Also, NMR studies of IκBα in complex with its binding partner, NF-κB, show a more flexible ankyrin 1–4 domain in comparison to rather rigid ankyrin repeats 5–6 (Sue et al., 2008). An earlier amide H/D exchange study (Truhlar et al., 2006) indicated that when in complex with NF-κB, ankyrin repeats five and six-fold into compact domains upon binding to NF-κB. Along with ankyrin repeats 5 and 6, ankyrin repeat 1 is another region seen to display greater conformational flexibility as observed here in the refined structure of IκBα. The RMSFs of amino acid residues mapped onto the Cα-backbone atoms of IκBα can be seen in the Supplementary Material, Figure 7.

### Conformational change induced in IκBα in its bound form to NF-κB

Thus, so far we have looked at the structural elements in IκBα only. In the crystal structures and in our simulations however, IκBα is in complex with NF-κB (the RelA/p50 heterodimer) and for this reason it is imperative to look at the conformation of IκBα in relation to its binding partner, NF-κB, and see how the nature of this association was affected. The protein-protein surface area of interaction is larger than 4000 Å^2^ and all six ankyrin repeat units are involved in forming a non-contiguous contact surface. We discuss here in particular electrostatic and hydrogen bonding interactions between IκBα and RelA/p50. The hydrogen bonds that are discussed here remain intact for longer than 10% of the simulation time and occur in at least two of the replicate simulations.

#### The IκBα/RelA interface

IκBα binds to RelA by forming a number of hydrogen bonds between different regions of each protein (Table 1). Several residues situated on ankyrin repeats 5 and 6 form hydrogen bonds with residues located on both the RelA dimerization subunit and the RelA amino-terminal. The IκBα carboxy-terminal residues are in close contact with regions on the amino-terminal and dimerization subunit of RelA and form several hydrogen bonds.

**Table 1:** **Hydrogen bond contacts between IκBα and the p50/RelA subunits of NF-κB**.

**IκBα**	**RelA**	**IκBα**	**p50**	**RelA**	**p50**
**HYDROGEN BONDS**					
GLY155	ARG297	ASP73	LYS352	ARG198	HIS304
LEU157	**ARG297**	GLN107	LYS352	**ASN200**	**ASP254**
ASN216	**ASP243**	ASN109	ASP353	**ASP243**	**ARG252**
ASP226	SER238	ASN109	LYS352	**HIS245**	CYS270
THR247	ASP243	**TYR181**	THR256		
GLN249	ASP243	**ASN182**	THR256		
**TRP258**	GLN26	GLY183	THR256		
**GLY259**	GLN241	CYS215	MET253		
GLN266	ILE24	**TYR248**	LYS343		
GLN267	**GLU22**	**TYR248**	GLU341		
GLN271	LEU179	GLN249	**ARG252**		
GLN271	VAL21				
LEU280	GLN29				
SER283	GLU222				
ASP285	GLN247				
GLU286	GLN247				
GLU286	THR191				
SER288	GLN247				

*Residues found in interactions in the crystal structure 1IKN are shown in bold. All Bonds are present for more than 10% of the total simulation time in at least 2 of the replicate simulations*.

The other major source of stabilization is via electrostatic interactions from the salt bridge interactions between the carboxy-terminal of IκBα and different regions of RelA (Table 2). The ARD region of IκBα contributes to the IκBα/RelA stabilization by forming salt bridges between Asp226 and Arg218 on ANK5 and between Arg253 and Asp243 as well as Glu211 on the dimerization component. In addition, Arg264 on ANK6 interacts with Glu22 on the amino-terminal of RelA. Specifically the interaction between Arg218 and Asp243 is also observed to form in the crystal structure of IκBα (Huxford et al., 1998).

**Table 2:** **Salt bridge formations between IκBα and the p50/RelA subunits of NF-κB**.

**IκBα**	**RelA**	**IκBα**	**p50**	**RelA**	**p50**
**SALT BRIDGES**					
GLU85	ARG302	GLU41	LYS354	ARG198	**ASP302**
GLU85	LYS301	GLU72	LYS352	ARG198	GLU265
GLU86	ARG302	GLU72	LYS354	ARG201	**ASP254**
GLU125	ARG302	ASP73	LYS354	ARG201	GLU265
ARG143	ASP294	GLU138	LYS323	**GLU211**	**ARG252**
GLU153	ARG295	ARG143	GLU350	**ASP217**	**ARG305**
GLU153	**ARG297**	GLU286	ARG305	**ASP243**	**ARG252**
**ARG218**	**ASP243**	GLU286	LYS272	**ARG246**	**ASP271**
ARG218	GLU211	GLU287	LYS272		
ASP226	ARG253	ASP290	LYS272		
ARG264	**GLU22**	GLU292	**LYS249**		
GLU282	ARG30	GLU292	LYS272		
GLU282	**ARG158**				
GLU282	LYS79				
GLU284	ARG246				
GLU284	LYS79				
ASP285	LYS218				
GLU286	LYS218				
GLU287	ARG246				
GLU287	LYS218				
ASP290	LYS221				
GLU292	ARG246				

*Residues found in interactions in the crystal structure 1IKN are shown in bold. All salt bridges occur in at least 2 of the replicate simulations throughout the whole simulation*.

The elongated and relatively flexible 13 residue carboxy-terminal of RelA, known as the NLS polypeptide, extends across ankyrin repeats 1–3 and makes several contacts with residues present on the loops and helical regions of these ankyrin repeats, forming both hydrogen bonds and salt bridges.

#### The IκBα/p50 interface

A number of residues on ankyrin repeats 4–6 interact with the dimerization domain on p50 by forming hydrogen bonds. Among these interactions, Tyr181 has previously been shown to be a key player in the interaction between NF-κB and IκBα (Huxford et al., 1998). Eminently, residues Cys215, Tyr248, and Arg252 on the p50 subunit are among those reported to form interactions in the crystal structure of IκBα. The amino acid residues Lys352-Asp353 located on the carboxy-terminal of p50 engage in additional hydrogen bond interaction with the residues Asp73, Gln107, and Asn109 situated on ankyrin repeats 1 and 2. The interaction between IκBα and p50 is further stabilized by electrostatic interactions. The carboxy-terminal PEST sequence residues Glu286-Glu287, Asp290, GLU292 in IκBα take part in forming salt bridges with the residues Lys249, Lys272, Arg305 on the amino-terminal and the interconnecting loops on the “top” of the p50 subunit. Ankyrin repeats 1–3 and the SRD in IκBα and the carboxy-terminal and an interconnecting loop at the “bottom” of p50 participate in another set of salt bridge network involving residues Glu41, Glu72-Asp73, Glu138, and Lys323, Lys352, Lys354, on respective chain. Notably, with one single exception, the acidic residues are contributed by IκBα, whereas the basic residues are to be found on the p50 subunit.

#### The RelA/p50 interface

The dimerization interface takes part in several hydrogen bonds formed by 8 residues including an Asp254(p50)/Asn200(RelA) hydrogen bond. This hydrogen bond can also be found in the crystal structure and is considered one of the most critical interactions in discriminating subunit dimerization specificity among NF-κB dimers (Huang et al., 1997; Chen et al., 1998; Huxford et al., 1998). The other hydrogen bonds include His304(p50)/Arg198(RelA), Arg252(p50)/Asp243(RelA), Cys270(p50)/His245(RelA). The RelA/p50 dimer interface is additionally stabilized by electrostatic interactions. Several residues form salt bridges between the two subunits. Two of these include salt bridges that are also reported for the crystal structure namely residues Asp217 and Asp271 on the p50 subunit and Arg305 and Arg246 on the RelA component, respectively (Huxford et al., 1998).

### Free IκBα vs. bound IκBα

All the simulations discussed above were describing the stable, long-living complex of IκBα with its binding partner NF-κB, as revealed in their crystal structures. All efforts to crystallize IκBα in its unbound state have been unsuccessful (Croy et al., 2004). For this reason, additional simulations of full-length free IκBα in solution were performed and compared with the more stable NF-κB-bound state.

**Figure 9:**
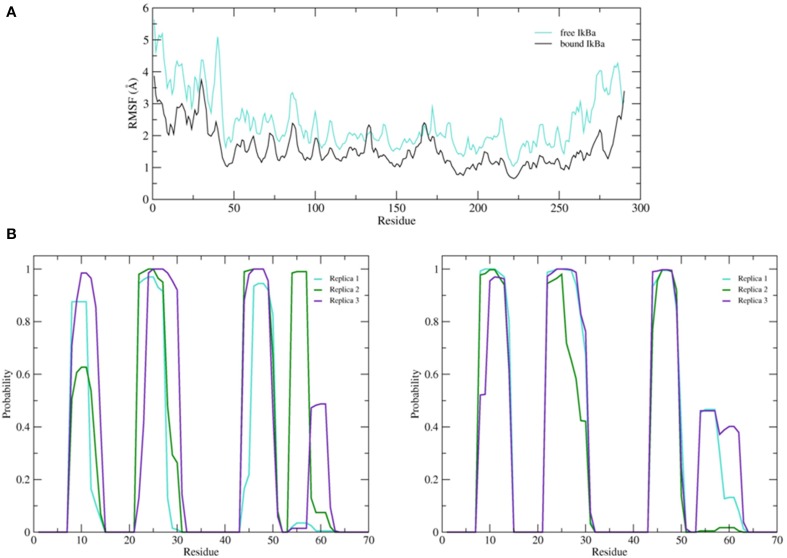
**(A)** Average root mean square fluctuations (RMSF) of the backbone of the free IκBα (cyan) in comparison to the one in complex with NF-κB (black). **(B)** Probability distributions of α-helix formation of the first 70 residues of the SRD. Left: free IκBα. Right: IκBα in complex with NF-κB.

The simulation setups followed the same procedure as for the bound IκBα, resulting in three replicated systems of 200 ns each. Conservation of total energy and temperature of the three simulations (Supplementary Material, Figure 8) points to systems that have reached a stable state. In contrast to the bound IκBα, the RMSF of the free state of IκBα remains on average ~1 Å higher compared to its complexed state. This points to a higher degree of flexibility of free IκBα compared to its complexed state. The RMSF of the backbone of the protein around the average structure in the modeled SRD remains the most flexible domain throughout the protein in addition to the unstructured C-terminal region (see Figure 9A top). The probability distributions of the helical propensity in the SRD of the bound IkBa reveal (Figure 9B) the first three helical segments to be stable throughout the whole simulation. In free IκBα, although the first three helical segments are present in all three replicate simulations, we observe different probabilities across the different replicate simulations. Bound IκBα displays a narrower distribution of probabilities of helical regions and this indicates to a stabilization of the SRD upon complexation with NF-κB. The fourth initially assigned helix in the SRD varies in both length and probability in both the bound and free forms of IκBα, indicating that this fourth helix is not well-defined and not stable during MD refinement (see above).

**Figure 10:**
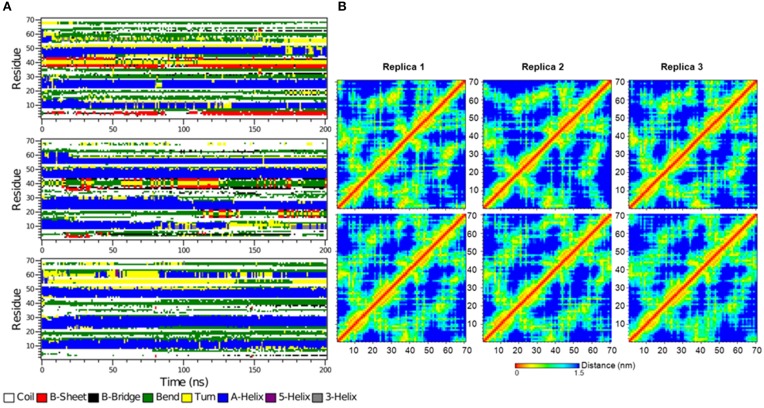
**(A)** The secondary structure elements of the first 70 N-terminal residues of free IκBα calculated by DSSP for the three system replicas for the entire simulation. **(B)** Interatomic distance matrices for the first 70 N-terminal residues of free IκBα (top) and in complex with NF-κB (bottom).

The secondary structure evolution of the first 70 amino acid residues in the SRD of the free IκBα (see Figure 10A) reveals greater differences in the SRD in terms of secondary structure element evolution in comparison to the bound IκBα. The first helix in the free IκBα is considerably shorter than its counterpart in the bound IκBα. During the first 100 ns of the simulations, this helix can be clearly distinguished whereas it is only present in two of the replicate runs in the final simulation period. The second and third helices remain intact throughout the entire 200 ns simulations in all three replicate runs, which is very similar to the pattern seen in the bound IκBα simulations. In contrast to the bound IκBα, here we observe the formation of a 4 residue long fourth helix in two of the replicate runs; in one of the simulations this helix is present during the entire simulation, whereas in the other run it appears in the last 100 ns of the simulations with irregular intervals. In a previous study (Pons et al., 2007), the conformations of a short 24 amino acid peptide (residues 21–44) of the doubly phosphorylated free IκBα were characterized by NMR spectroscopy and MD simulations and compared to its β-TrCP bound state using saturation transfer difference NMR. The conformational observation agreed on the presence of a bend between residues 30 and 36 in both states of the phosphorylated peptide, a trend which we also observe throughout our simulations of the free and NF-κB bound states of IκBα. While the N-terminal of amino acids 30 to 36 is preceded by a short α-helix and the C-terminal succeeded by a region of β-sheet–turn–β-sheet flanked by bends in the free and bound states of IκBα in this study, Pons et al. observed disordered N- and C-terminal segments in the free IκBα vs. the adoption of turns in the bound state IκBα. This difference in results can be rationalized from the truncation of the peptide which could have influenced the conformational integrity of the N- and C-terminals, an effect which would not be detectable in our structural models of the full-length IκBα.

Figure 10B shows the interatomic distance matrices depicting the smallest distance between residue pairs in the SRD of IkBa for both free (top) and complexed IκBα (bottom). The distance matrices of all three replicates are very similar and there are no large differences in interatomic distances upon NF-κB binding. The red and yellow colors indicate shorter distances between the residues and are more detectable for regions where helical segments are present in the SRD. In both the unbound and free forms of IκBα, the fourth segment is less apparent across the replicates.

There are, however, also apparent stretches of amino acids which display a higher degree of flexibility upon NF-κB complexation (see Figure 9A; top). The residues around positions 133 and 167 become more flexible upon protein-protein complex formation. These positions correspond to loop regions following the outer helices in AR2 and AR3. This was also found by analyzing residual dipolar coupling (RDC) of ARs 1–4 (Cervantes et al., 2009).

Another interesting comparison between the free and bound IκBα structures is the solvent accessible surface area (SASA) or the relative solvent accessible area (RSA) of the phosphorylation and ubiquitination sites located on the SRD (Table 3). These sites (Ser32 and Ser36 for phosphorylation and Lys21 or Lys22 for ubiquitination) ought to become accessible by the kinase IKK and the E3 ligase, respectively, in the complexed form of IκBα. The RSA is computed by the SASA of the residue normalized by the accessible surface area of that residue in its extended tri-peptide (Gly-X-Gly) conformation. By setting a threshold of < 20% for buried residues, Ser32 and Ser36 are both surface-exposed in the bound IκBα, while in its free state only Ser32 lies above the threshold. SER36 in the free state has an RSA of 9.3%, which is considerably lower than the threshold and can be considered to be a buried residue. As regards to the ubiquitination sites, Lys21 stays well-buried in both the free and bound states of IκBα. However, Lys22 with an RSA of well over 60% in both states of IκBα remains surface exposed. Thus, in the bound-form of IκBα the phosphorylation Ser32 and Ser36 sites are accessible by the IKK and we suggest Lys22 to be the putative site of ubiquitination.

**Table 3:** **Solvent accessible surface area (SASA) and relative surface area (RSA) of the free and bound IκBα**.

	**SASA (Å)**		**RSA (%)**	
	**Bound IκBα**	**Free IκBα**	**Bound IκBα**	**Free IκBα**
SER32	61.2 ± 18.5	43.4 ± 15.7	50.2 ± 15.2	35.6 ± 12.8
SER36	50.6 ± 14.0	11.3 ± 9.1	41.5 ± 11.5	9.3 ± 7.4
LYS21	18.5 ± 4.8	39.2 ± 16.4	8.8 ± 2.3	18.6 ± 7.8
LYS22	135.6 ± 6.0	127.5 ± 17.9	64.3 ± 2.8	60.4 ± 8.5

*The accessible surface areas of serine and lysine are 122 Å and 211 Å, respectively as calculated by Miller et al. (1987). The SASA values are the averages of the three replicate simulations over the 200 ns of total simulation time*.

## Conclusion and outlook

The lack of crystallographic information about the SRD of IκBα has led to the speculation of a disordered N-terminal extension that could not be crystallized. Furthermore, the SRD was shown not to be a major contributor to the IκBα/NF-κB binding affinity (Hatada et al., 1992; Jaffray et al., 1995; Sun et al., 1996). This particular region, however, contains the two highly conserved serine residues, 32 and 36, which are the sites of phosphorylation by IKKs and involved in the regulation of IκBα. After two-fold phosphorylation, the IκBα/NF-κB becomes poly-ubiquinitated at Lysine residues 21 and 22, the protein-protein complex releases NF-κB and IκBα to be degraded *in vivo*. Thus, investigation of dynamical and structural properties of this domain is very important for understanding of the post-translational modifications and signaling properties of this domain.

Previous structural investigations (Mizuguchi and Blundell, 2000; Ferreiro and Komives, 2010) have used bioinformatics tools like PONDR (Geourjon et al., 2001) and IUPRED (Simons et al., 1997) to annotate the potential disorder and flexibility of the ankyrin units of the ARD. For IκBα (67–317) the β-hairpin loops of each ankyrin repeat displayed a greater degree of disorder than the α-helical regions. Furthermore, ankyrin repeat units 2, 3, and 4 were more structured than units 1, 5, and 6. In particular the C-terminal part of the ARD and the PEST domain were considered as being intrinsically disordered. For the SRD, however, the results were not unambiguous.

The lack of sequence identity to any known three-dimensional protein structure obstructed the comparative modeling approach. A predicted well-ordered secondary structure profile of the SRD, however, allowed the assignment of α-helical structural elements in this region. Protein domain threading suggested the poly-ankyrin human ankyrinR as a suitable structural but not sequence-based model. From a secondary structure alignment, a structural model for the SRD of IκBα in complex with NF-κB was generated and refined by multiple-template MD simulations. In the final model, the SRD region was shown to contain three stable α-helices. The MD simulations resolved ambiguities of secondary structure elements for residues 54–63 which were α-helical in the template but rather occupied a loop conformation after MD refinement.

The structural stability of the model has been validated through long MD simulations. For the protein domain annotation of IκBα, our results clearly display stable helical conformations in the N-terminal SRD. Interestingly, the amino acid sequence composition of the SRD is in good agreement with the consensus sequence for typical ankyrin repeat domains (Mosavi et al., 2002, 2004). This provides additional support for the stability of secondary structural elements in the SRD. The C-terminal PEST domain, however, displays large atomic fluctuations and a high degree of flexibility, which make the reliable assignment of any secondary structural element impossible.

When compared to free IκBα in solution, the complexed IκBα displays a significantly reduced degree of intrinsic flexibility and disorder. In particular, the SRD and the PEST domains show significantly reduced flexibility upon NF-κB binding. For the central ARD, the picture is less clear. Different ARs were shown to possess different solvent accessibilities by H/D exchange and MS (Croy et al., 2004). This was interpreted as an increased structural flexibility for ARs 1, 5, and 6 but retaining all of the SSEs at the same time. According to our results, however, the degree of solvent accessibility is not determined by helical flexibility of ARs 1, 5, 6 but rather by their adjacent hairpin/loop regions. This is in agreement with the first four ankyrin repeat units exhibiting little change in solvent accessibility upon NF-κB binding (Truhlar et al., 2006) whereas ARs 5 and 6 undergoing a coupled folding and binding process. When we compared the free IκBα with the IκBα bound to NF-κB the order parameters from NMR and aMD compared well and showed not drastic structural rearrangement upon complexation (Cervantes et al., 2009). However, for IκBα the order parameters were generally lower for variable loop regions parameters when in complex with NF-κB than for free IκBα. Our simulations can rationalize this finding in terms of an increase in intrinsic flexibility of the loop regions upon complexation and thus it is in good agreement with the hypothesis of a degree of protein “fuzziness” in the IκBα/NF-κB complex (Komives, 2012).

To experimentally address the structural composition of the IκBα N-terminal SRD, it is desirable to perform NMR studies using a recombinant full-sequence IκBα in free solution and/or in complex with NF-κB. The overexpression of human IκBα in sufficient yield is currently being performed in our laboratory.

The complete structural model of IκBα in complex with NF-κB has now been prepared and will be used as starting structures for upcoming multi-scale investigations regarding the structural basis for IκBα signaling after phosphorylation and ubiquitination at the molecular level.

### Conflict of interest statement

The authors declare that the research was conducted in the absence of any commercial or financial relationships that could be construed as a potential conflict of interest.

## Footnotes

^1^Optimally Segmented Consensus Secondary Structure Prediction. SYMPRED is available online at: www.ibi.vu.nl/programs/sympredwww/.
