# Supplementary material for: Structural modeling of the N-terminal signal–receiving domain of IκBα
Source: Front Mol Biosci. 2015 Jun 23;2:32. doi: 10.3389/fmolb.2015.00032 (PMC4477481; doi:10.3389/fmolb.2015.00032)
Supplement: Supplementary file 1 [file DataSheet1.PDF]

Supplementary Material

Structural modeling of the N-terminal signal–receiving domain of IκBα

Samira Yazdi[a], Serdar Durdagi[a,b], Michael Naumann[c], Matthias Stein [a*]
[a]Max Planck Institute for Dynamics and Complex Technical Systems, Molecular Simulations and Design Group, Sandtorstrasse 1, 39106 Magdeburg, Germany.
[b]Current addresss:Department of Biophysics, School of Medicine, Bahcesehir University, Istanbul, Turkey.
[c]Institute of Experimental Internal Medicine, Medical Faculty, Otto von Guericke University, Leipziger Strasse 44, 39120 Magdeburg, Germany.

*** Correspondence:** Corresponding Author, Dr. Matthias Stein, Molecular Simulations and Design Group, Max Planck Institute for Dynamics of Complex Technical Systems, Sandtorstrasse 1, 39106 Germany. Fax +49-391-6110403. Email matthias.stein@mpi-magdeburg.mpg.de

Supplementary Data

- **Supplementary Figure 1**. A comparison of secondary structure predictions of the full length IκBα using different methods.

- **Supplementary Figure 2**. Results from JPRED3 analysis of the full 317 amino acid residues from human IκBα with confidence scores.

- **Supplementary Figure 3**. pDomThreader prediction results.

- **Supplementary Figure 4**. The total energy variation of the three system replicas for the initial (A) and final (B) 100ns simulation.

- **Supplementary Figure 5**. Temperature of the three system replicas for the initial 100 ns (A) and the final 100ns (B).

- **Supplementary Figure 6**. RMSD of the complexed IκBα/NF-κB protein backbone against the starting structure of the three system replicas for the initial 100 ns (A) and the final 100ns (B).

- **Supplementary Figure 7.** Root mean square fluctuations of amino acid residues mapped onto Cα-backbone atoms of IκBα.

- **Supplementary Figure 8.** (A) The total energy variation of the three system replicas for the free IκBα simulations.(B) Temperature of the three system replicas for the free IκBα simulations

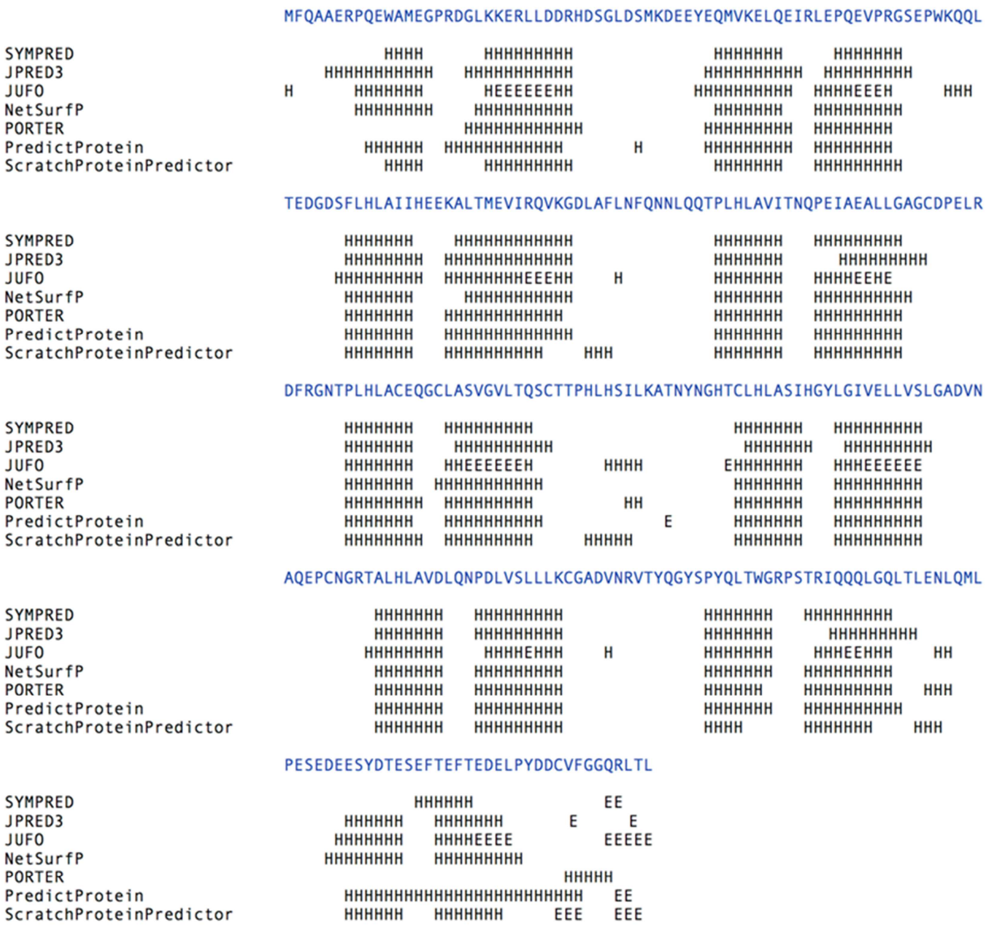

Supplementary Figure 1. A comparison of secondary structure predictions of the full length IκBα using different methods.

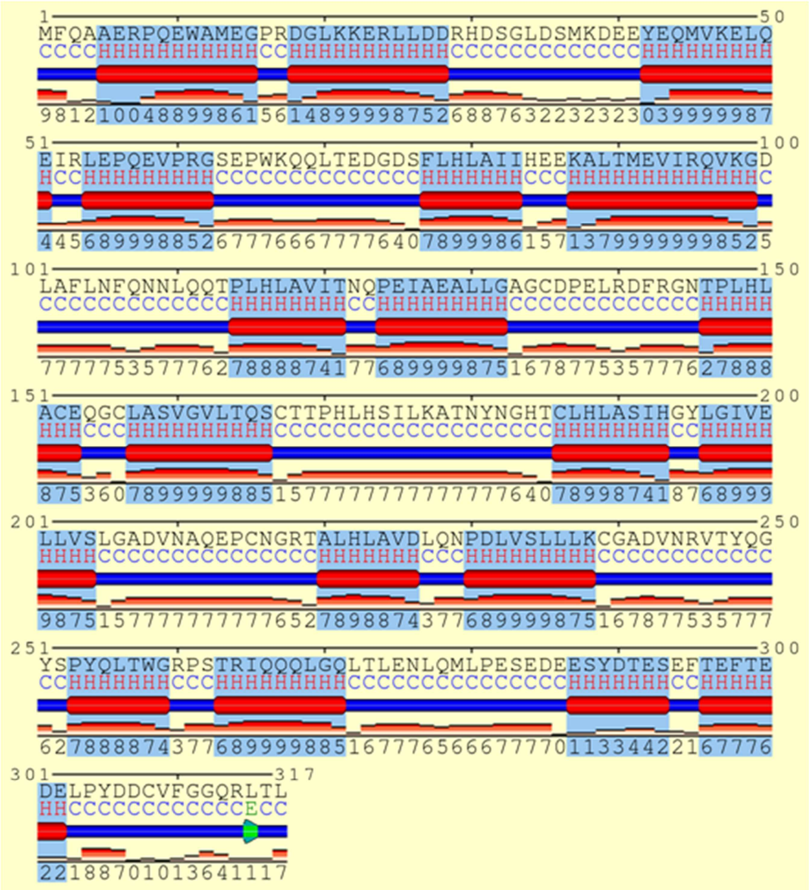

Supplementary Figure 2. Results from JPRED analysis of the full 317 amino acid residues from human IκBα. Two helix-coil-helix motifs can be recognized between residues 5-62 which is indicative of an ankyrin repeat-like organization of the SRD.

```
domTHREADER PREDICTION RESULTS:
HTML formatted results can be found at:
http://bioinf.cs.ucl.ac.uk/psipred/result/333460
You can also view the results in a machine printable format at:
http://bioinf.cs.ucl.ac.uk/genthreader_output/333460.html

- Key ------------------------------------------------------------
Conf   = Description of confidence level
Score  = Raw score from SVM
p-val  = Probability of false positive
Epair  = Pairwise energy for model
Esolv  = Solvation energy for model
AlnSc  = Sequence alignment score
Alen   = Length of alignment
Dlen   = Length of PDB entry
Tlen   = Length of target sequence
Start  = Start position of the domain
Stop   = End position of the domain
PDB_ID = PDB identifier (+ chain code + domain code in CATH format)
------------------------------------------------------------------
Confidence levels:
CERT   p-value < 0.0001
HIGH   p-value < 0.001
MEDIUM p-value < 0.01
LOW    p-value < 0.1
GUESS  p-value >= 0.1
------------------------------------------------------------------
```

CERT	11.734	2e-08	-287.9	1.6	549.5	216	216	317	73	293	1iknD00
CERT	11.479	2e-08	-291.4	-1.3	473.4	126	126	317	108	242	1n0rA00
CERT	11.232	3e-08	-203.6	-0.7	408.8	93	93	317	141	242	1n0qB00
CERT	11.142	3e-08	-262.6	0.3	627.0	272	404	317	1	286	1n11A00
CERT	10.815	4e-08	-295.3	-6.7	364.0	153	153	317	108	270	1awcB00
CERT	10.750	5e-08	-373.3	-7.6	470.0	216	228	317	72	317	1k1aA00
CERT	10.541	6e-08	-240.0	-0.6	327.0	131	131	317	100	237	1ympA00
CERT	10.521	6e-08	-336.6	-6.9	415.0	200	285	317	73	287	1wdyA00
CERT	10.505	6e-08	-279.6	-3.1	409.0	152	155	317	112	270	2bkgA00
CERT	10.471	7e-08	-380.9	-5.8	406.0	214	223	317	36	271	1uohA00
CERT	10.469	7e-08	-389.9	-4.5	405.0	204	209	317	55	272	2f8yB00
CERT	10.053	1e-07	-401.7	-4.9	351.0	215	226	317	35	274	2dznA00
CERT	9.872	1e-07	-301.0	-0.3	324.0	155	156	317	73	240	1bd8A00
CERT	9.872	1e-07	-244.9	2.0	320.0	150	162	317	83	242	1s70B01
CERT	9.559	2e-07	-189.0	0.7	299.0	122	124	317	136	267	3c5rA00
CERT	9.433	2e-07	-228.6	-2.2	298.0	123	128	317	114	242	1ycsB01
CERT	9.431	2e-07	-186.3	-0.6	259.0	125	125	317	109	241	1bi7B00
CERT	9.029	4e-07	-110.3	-2.1	244.0	93	93	317	40	136	1n0qB00
CERT	9.016	4e-07	-144.4	2.2	267.0	125	129	317	112	243	1s70B02
CERT	7.289	3e-06	-208.8	-3.0	205.0	215	254	317	15	259	1sw6A00
HIGH	5.818	2e-05	-137.0	-3.9	164.0	96	124	317	35	136	3c5rA00
HIGH	5.697	2e-05	36.1	4.7	164.0	98	126	317	4	108	1n0rA00
HIGH	5.411	2e-05	37.7	7.9	157.0	105	155	317	1	112	2bkgA00
HIGH	4.308	9e-05	22.1	1.4	132.0	103	129	317	6	112	1s70B02
MEDIUM	4.165	1e-04	0.2	1.0	129.0	99	128	317	8	114	1ycsB01
MEDIUM	3.991	1e-04	50.7	7.3	125.0	98	153	317	1	108	1awcB00
MEDIUM	3.163	3e-04	78.3	2.5	108.0	91	131	317	1	98	1ympA00
LOW	2.171	0.001	51.5	0.8	89.0	80	162	317	1	83	1s70B01
LOW	1.936	0.001	0.1	2.7	84.1	15	61	317	303	317	2pw8I00
LOW	1.905	0.001	-28.1	2.8	83.0	34	125	317	72	109	1bi7B00
LOW	1.464	0.002	-0.0	1.9	75.3	16	132	317	302	317	1gupA02
LOW	1.131	0.004	80.0	6.0	69.0	70	285	317	1	73	1wdyA00
LOW	1.061	0.004	-0.0	-0.3	68.7	15	29	317	302	317	1ubdC04
LOW	1.031	0.004	-3.3	-1.0	68.7	14	205	317	301	317	1vg0A01
LOW	0.905	0.005	58.5	1.5	65.0	69	228	317	1	72	1k1aA00
LOW	0.770	0.006	1.0	-1.4	64.2	17	115	317	299	317	2r6fA04
LOW	0.692	0.006	-1.7	-1.6	62.9	15	230	317	303	317	2nvkX01
LOW	0.686	0.006	60.7	1.3	61.0	70	216	317	1	73	1iknD00
LOW	0.427	0.008	-2.8	2.2	56.0	37	93	317	1	40	1n0qB00
LOW	0.380	0.009	-204.1	-9.2	53.0	163	196	317	65	244	1tx4A00
LOW	0.309	0.010	-39.2	-2.1	43.0	72	72	317	62	145	1pveA00
LOW	0.266	0.010	-32.9	-2.6	43.0	52	52	317	19	75	1rquA01
GUESS	0.104	0.012	32.9	4.4	50.0	52	209	317	1	55	2f8yB00
GUESS	0.103	0.012	65.8	5.0	50.0	69	156	317	1	73	1bd8A00
GUESS	0.018	0.013	-31.6	-5.0	39.0	57	57	317	208	301	1tteA02
GUESS	0.011	0.013	-16.6	-1.2	38.0	56	56	317	209	284	1h9eA00

```
>>> Alignment with 1iknD00:
                 10        20        30        40        50        60
         CCCCCCHHHHHCCCCCCCCCCCCCCCCCCCCCCCCCHHHHHHHCCCHHHHHCCCCCCCCC
1iknD00  DGDSFLHLAIIHEEKALTMEVIRLAFLNFQNNLQQTPLHLAVITNQPEIAEALLGAGCDP

Query    ------------------------------------------------------------
         ------------------------------------------------------------
```

```
                 70        80        90       100       110
         CCCCCCCCCHHHHHHHHCCHHHHHHHHHCCCCCCCCCCCCCCCCCCCCHHHHHHHCCC--
1iknD00  ELRDFRGNTPLHLACEQGCLASVGVLTQSCTTPHLHSILKATNYNGHTCLHLASIHGY--
                                                 |           |
Query    -------------------------------------MFQA----AERPQEWAMEGPRDG
         -------------------------------------CCCC----CCCCHHHHHCCCCCC
                                                          10


         120       130       140       150       160       170
         -HHHHHHHHHHCCCCCCCCCCCCCCHHHHHHHHCCCHHHHHHHHHCCCCCCCCCCCCCCCCHH
1iknD00  -LGIVELLVSLGADVNAQEPCNGRTALHLAVDLQNPDLVSLLLKCGADVNRVTYQGYSPY
               |                                             |
Query    LKKERLLDDRHDSGLDS-MKDEEYEQMVKELQEIRLEPQEVPRGSEPWKQQLTED-----
         HHHHHHHHHHCCCCCCC-CCCCCHHHHHHHHHHHHCCCCCCCCCCCCCCCCCCCC-----
                  30        40        50        60        70


         180       190       200       210
         HHCCCCCCHHHHHHHHCCCCCCCCCCCCCCCCCCCCCCC
1iknD00  QLTWGRPSTRIQQQLGQLTLENLQMLPESEDEESYDTE-

Query    ---------------------------------------
         ---------------------------------------


Percentage Identity = 1.9.

>>> Alignment with 1n0qB00:
                 10        20        30        40          50
         CCCCHHHHHHHHCCCHHHHHHHHHHHCCCCCCCCCCCCHHHHHHHCCC--HHHHHHHHHC-
1n0qB00  NGRTPLHLAARNGHLEVVKLLLEAGADVNAKDKNGRTPLHLAARNGH--LEVVKLLLEA-
                                              |   |       |    ||
Query    ------------------------------MFQAAERPQEWAMEGPRDGLKKERLLDDRH
         ------------------------------CCCCCCCCHHHHHCCCCCHHHHHHHHHHHC
                                               10        20        30


          60        70        80        90
         CCCCCCCCCCCCHHHHHHHCCCHHHHHHHHHHCCCC
1n0qB00  GADVNAKDKNGRTPLHLAARNGHLEVVKLLLEAGAY

Query    DSGLDSMKDE--------------------------
         CCCCCCCCCC--------------------------
                 40


Percentage Identity = 5.4.

>>> Alignment with 1tx4A00:

         ------------------------------------------------------------
1tx4A00  ------------------------------------------------------------

Query    MFQAAERPQEWAMEGPRDGLKKERLLDDRHDSGLDSMKDEEYEQMVKELQEIRLEPQEVP
         CCCCCCCCHHHHHCCCCCCHHHHHHHHHHHCCCCCCCCCCCCHHHHHHHHHHHCCCCCCC
                 10        20        30        40        50        60


                       10        20        30          40        50
         ----CCCCCC--CCCCHHHHHHHCCCCCCCHHHHHHHHHHHHHHC--CCCCCCCCCCC--
1tx4A00  ----PLPNQQ--FGVSLQHLQEKNPEQEPIPIVLRETVAYLQAHA--LTTEGIFRRSA--
             |   |    | |  ||     |         |           |
Query    RGSEPWKQQLTEDGDSFLHLAIIHEEK----ALTMEVIRQVKGDLAFLNFQNNLQQTPLH
         CCCCCCCCCCCCCCCCHHHHHHHCCCH----HHHHHHHHHHHHCCCCCCCCCCCCCHHHH
                 70        80            90       100       110


                       60        70        80             90
         ------CHHHHHHHHHHHHHCCCCCCCCCCCCHHHH-----HHHHHHHHHCCCCCCCHHH
1tx4A00  ------NTQVVREVQQKYNMGLPVDFDQYNALHLPA-----VILKTFLRELPEPLLTFDL
                                 ||     |||                   | |
Query    LAVITNQPEIAEALLGAGCDPELRDFRGNTPLHLACEQGCLASVGVLTQSCTTPHLH---
         HHHHCCCHHHHHHHHHHCCCCCCCCCCCCCHHHHHHHCCHHHHHHHHHHCCCCCCCC---
          120       130       140       150       160       170


                 110       120       130       140       150
         HHHHHCCCCCHHHHHHHHHHHHHCCCCHHHHHHHHHHHHHHHHHHHCHHHHCCCHHHHHH
1tx4A00  YPHVVGFLNIDESQRVPATLQVLQTLPEENYQVLRFLTAFLVQISAHSDQNKMTNTNLAV
                 |          |                |        |    |  |   |||
Query    --SILKATNYN--GHTCLHLASIHG----YLGIVELLVSLGADVNAQEPCNGRTALHLAV
         --CCCCCCCCC--CCHHHHHHHHCC----CHHHHHHHHHCCCCCCCCCCCCCCHHHHHHH
               180         190           200       210       220
```

```
                 170       180       190
         HHHHHHCCCCCHHHHHHCHHHHHHHHHHHHHCHHHHC-----------------------
1tx4A00  VFGPNLLWAKDAAITLKAINPINTFTKFLLDHQGELF-----------------------
               |                     ||
Query    DLQNPDL------------------VSLLLKCGADVNRVTYQGYSPYQLTWGRPSTRIQQ
         HCCCHHH------------------HHHHHHCCCCCCCCCCCCCHHHHHHHCCCHHHHHH
           230                         240       250       260


         --------------------------------------------------
1tx4A00  --------------------------------------------------

Query    QLGQLTLENLQMLPESEDEESYDTESEFTEFTEDELPYDDCVFGGQRLTL
         HHHHCCCCCCCCCCCCCCCCCCCCHHCCCCCCCCCCCCCCCCCCCCCCCC
         270       280       290       300       310

Percentage Identity = 14.3.

>>> Alignment with 1pveA00:

         ------------------------------------------------------------
1pveA00  ------------------------------------------------------------

Query    MFQAAERPQEWAMEGPRDGLKKERLLDDRHDSGLDSMKDEEYEQMVKELQEIRLEPQEVP
         CCCCCCCHHHHHHCCCCCCHHHHHHHHHHCCCCCCCCCCCHHHHHHHHHHHCCCCCCCCC
                 10        20        30        40        50        60

                  10        20        30                    40
         -CCCCCCCCCCCCCCCCCHHHHHCCCHHHHHHHHHHHHC------------CCHHHHHHH
1pveA00  -GSHMPLEFLRNQPQFQQMRQIIQQNPSLLPALLQQIGR------------ENPQLLQQI
          ||      |           ||     |      |                 |  |  |
Query    RGSEPWKQQLTEDGDSFLHLAIIHEEKALTMEVIRQVKGDLAFLNFQNNLQQTPLHLAVI
         CCCCCCCCCCCCCCCHHHHHHHCCCHHHHHHHHHHHHHHCCCCCCCCCCCCHHHHHHHHH
                 70        80        90       100       110       120

          50        60        70
         HCCHHHHHHHHCCCCCCHHHHCCCC-----------------------------------
1pveA00  SQHQEHFIQMLNEPVQEAGGQGGGG-----------------------------------
             |     |            |
Query    TNQPEIAEALLGAGCDPELRDFRGNTPLHLACEQGCLASVGVLTQSCTTPHLHSILKATN
         CCCHHHHHHHHHCCCCCCCCCCCCCCHHHHHHHCCHHHHHHHHHHCCCCCCCCCCCCCCC
                130       140       150       160       170       180


         --------------------------
1pveA00  --------------------------

Query    YNGHTCLHLASIHGYLGIVELLVSLG
         CCCCHHHHHHHHCCCHHHHHHHHHCC
                190       200

Percentage Identity = 18.1.

>>> Alignment with 1rquA01:
                                   10             20        30
         ------------------CCCHHHHHHHHC-----CCCHHHHHHHHHHHHHCCCCCCCCC
1rquA01  ------------------SITKDQIIEAVA-----AMSVMDVVELISAMEEKFGVSAAAA
                              |              |             |
Query    MFQAAERPQEWAMEGPRDGLKKERLLDDRHDSGLDSMKDEEYEQMVKELQEIRLEPQEVP
         CCCCCCCHHHHHHCCCCCCHHHHHHHHHHCCCCCCCCCCCHHHHHHHHHHHCCCCCCCCC
                 10        20        30        40        50        60

          40        50
         CCCCCCCCCCCCCCC---------------------------------------------
1rquA01  VAVAAGPVEAAEEKT---------------------------------------------
                    |
Query    RGSEPWKQQLTEDGDSFLHLAIIHEEKALTMEVIRQVKGDLAFLNFQNNLQQTPLHLAVI
         CCCCCCCCCCCCCCCHHHHHHHCCCHHHHHHHHHHHHHHCCCCCCCCCCCCHHHHHHHHH
                 70        80        90       100       110       120


         ------------------------------------------------------------
1rquA01  ------------------------------------------------------------

Query    TNQPEIAEALLGAGCDPELRDFRGNTPLHLACEQGCLASVGVLTQSCTTPHLHSILKATN
         CCCHHHHHHHHHCCCCCCCCCCCCCCHHHHHHHCCHHHHHHHHHHCCCCCCCCCCCCCCC
                130       140       150       160       170       180
```

```
         ------------------------------------------------------------
1rquA01  ------------------------------------------------------------

Query    YNGHTCLHLASIHGYLGIVELLVSLGADVNAQEPCNGRTALHLAVDLQNPDLVSLLLKCG
         CCCCHHHHHHHHCCCHHHHHHHHHCCCCCCCCCCCCCHHHHHHHHHCCHHHHHHHHHHCC
                190       200       210       220       230       240


         ------------------------------------------------------------
1rquA01  ------------------------------------------------------------

Query    ADVNRVTYQGYSPYQLTWGRPSTRIQQQLGQLTLENLQMLPESEDEESYDTESEFTEFTE
         CCCCCCCCCCCCHHHHHHHCCCHHHHHHHHHCCCCCCCCCCCCCCCCCCCCHHCCCCCCC
                250       260       270       280       290       300


         -----------------
1rquA01  -----------------

Query    DELPYDDCVFGGQRLTL
         CCCCCCCCCCCCCCCCC
                310

Percentage Identity = 7.7.

>>> Alignment with 2f8yB00:
                 10        20        30        40        50        60
         CCCCHHHHCCCCCCCCCCCCCCCHHHHHHHCCHHHHHHHHHHCCCCCCCCCCCCCCHHHH
2f8yB00  AVISDFIYQGASLHNQTDRTGETALHLAARYSRSDAAKRLLEASADANIQDNMGRTPLHA

Query    ------------------------------------------------------------
         ------------------------------------------------------------


                 70        80        90       100       110       120
         HHHHCCHHHHHHHHCCCCCCCCCCCCCCCHHHHHHHHHCHHHHHHHHHHCCCCCCCCCCC
2f8yB00  AVSADAQGVFQILIRNRATDLDARMHDGTTPLILAARLAVEGMLEDLINSHADVNAVDDL

Query    --------------------------------------------------------MFQA
         --------------------------------------------------------CCCC


                130          140       150       160       170
         CCCHHHHHHHC---CCHHHHHHHHCCCCCCCCCCCCCCHHHHHHHHHCCHHHHHHHHHCC
2f8yB00  GKSALHWAAAV---NNVDAAVVLLKNGANKDMQNNREETPLFLAAREGSYETAKVLLDHF
               ||                      |     |         |   |
Query    AERPQEWAMEGPRDGLKKERLLDDRHDSGLDSMKDEEYEQMVKELQEIRLE---------
         CCCCHHHHHCCCCCHHHHHHHHHHHCCCCCCCCCCCHHHHHHHHHHHCCCC---------
             10        20        30        40        50

         180       190       200
         CCCCCCCCCCCHHHHHHHHCCCHHHHHHHHHC
2f8yB00  ANRDITDHMDRLPRDIAQERMHHDIVRLLDEY

Query    --------------------------------
         --------------------------------


Percentage Identity = 2.9.

>>> Alignment with 1bd8A00:
                 10        20        30        40        50        60
         CHHHHHHHHHHHCCHHHHHHHHHCCCCCCCCCCCCCCCHHHHCCCCCHHHHHHHHHCCCC
1bd8A00  RAGDRLSGAAARGDVQEVRRLLHRELVHPDALNRFGKTALQVMMFGSTAIALELLKQGAS

Query    ------------------------------------------------------------
         ------------------------------------------------------------


                 70        80          90       100       110
         CCCCCCCCCCHHHHHHHCCC---HHHHHHHHHCCCCCCCCCCCCCHHHHHHHHCCHHHHH
1bd8A00  PNVQDTSGTSPVHDAARTGF---LDTLKVLVEHGADVNVPDGTGALPIHLAVQEGHTAVV
                   |   |                 |                   ||
Query    ---MFQAAERPQEWAMEGPRDGLKKERLLDDRHDSGLDSMKDEEYEQMVKELQEIRLEPQ
         ---CCCCCCCCHHHHHCCCCCHHHHHHHHHHHCCCCCCCCCCCHHHHHHHHHHHCCCCCC
                    10        20        30        40        50

         120        130       140       150
         HHHHC-CCCCCCCCCCCCCHHHHHHCCCHHHHHHHHHCCC
1bd8A00  SFLAA-ESDLHRRDARGLTPLELALQRGAQDLVDILQGHM
```

```
               |
Query    EVPRGSEPWKQQLTED------------------------
         CCCCCCCCCCCCCCCC------------------------
          60        70
```

Percentage Identity = 3.8.

```
>>> Alignment with 1tteA02:

         ------------------------------------------------------------
1tteA02  ------------------------------------------------------------

Query    MFQAAERPQEWAMEGPRDGLKKERLLDDRHDSGLDSMKDEEYEQMVKELQEIRLEPQEVP
         CCCCCCCCHHHHHCCCCCCHHHHHHHHHHCCCCCCCCCCCHHHHHHHHHHCCCCCCCCCC
                 10        20        30        40        50        60


         ------------------------------------------------------------
1tteA02  ------------------------------------------------------------

Query    RGSEPWKQQLTEDGDSFLHLAIIHEEKALTMEVIRQVKGDLAFLNFQNNLQQTPLHLAVI
         CCCCCCCCCCCCCCCCCHHHHHHHCCCHHHHHHHHHHHHHHCCCCCCCCCCCCCHHHHHHHH
                 70        80        90       100       110       120


         ------------------------------------------------------------
1tteA02  ------------------------------------------------------------

Query    TNQPEIAEALLGAGCDPELRDFRGNTPLHLACEQGCLASVGVLTQSCTTPHLHSILKATN
         CCCHHHHHHHHHCCCCCCCCCCCCCCHHHHHHHCCCHHHHHHHHHCCCCCCCCCCCCCCC
                130       140       150       160       170       180

                                             10                  20
         ---------------------------CCC-CCCCCCC----------CHHHHHHHHHHC
1tteA02  ---------------------------NVE-ESDLYGI----------DHDLIDEFESQG
                                     |       |             ||       |
Query    YNGHTCLHLASIHGYLGIVELLVSLGADVNAQEPCNGRTALHLAVDLQNPDLVSLLLKCG
         CCCCHHHHHHHHHCCCHHHHHHHHHHCCCCCCCCCCCCCHHHHHHHHCCCHHHHHHHHHHCC
                190       200       210       220       230       240

                                  30               40        50
         -------------------CCHHHHHHHHHHCCC-------CCCCCCCCHHHHHHHHHHC
1tteA02  -------------------FEKDKIVEVLRRLGV-------KSLDPNDNNTANRIIEELL
                                 |   |  |          | |     |     |
Query    ADVNRVTYQGYSPYQLTWGRPSTRIQQQLGQLTLENLQMLPESEDEESYDTESEFTEFTE
         CCCCCCCCCCCCHHHHHHHCCCHHHHHHHHHHCCCCCCCCCCCCCCCCCCCHHCCCCCCC
                250       260       270       280       290       300


         C----------------
1tteA02  K----------------

Query    DELPYDDCVFGGQRLTL
         CCCCCCCCCCCCCCCCC
                310
```

Percentage Identity = 21.1.

```
>>> Alignment with 1h9eA00:

         ------------------------------------------------------------
1h9eA00  ------------------------------------------------------------

Query    MFQAAERPQEWAMEGPRDGLKKERLLDDRHDSGLDSMKDEEYEQMVKELQEIRLEPQEVP
         CCCCCCCCHHHHHCCCCCCHHHHHHHHHHCCCCCCCCCCCHHHHHHHHHHCCCCCCCCCC
                 10        20        30        40        50        60


         ------------------------------------------------------------
1h9eA00  ------------------------------------------------------------

Query    RGSEPWKQQLTEDGDSFLHLAIIHEEKALTMEVIRQVKGDLAFLNFQNNLQQTPLHLAVI
         CCCCCCCCCCCCCCCCCHHHHHHHCCCHHHHHHHHHHHHHHCCCCCCCCCCCCCHHHHHHHH
                 70        80        90       100       110       120


         ------------------------------------------------------------
1h9eA00  ------------------------------------------------------------

Query    TNQPEIAEALLGAGCDPELRDFRGNTPLHLACEQGCLASVGVLTQSCTTPHLHSILKATN
```

```
         CCCHHHHHHHHHCCCCCCCCCCCCCHHHHHHHHCCCHHHHHHHHHCCCCCCCCCCCCCCC
                130       140       150       160       170       180


                                             10                 20
         ----------------------------CCCCCCCCCCC---------CCHHHHHHHHCC
1h9eA00  ----------------------------PEFLEDPSVLT---------KDKLKSELVANN
                                         |     |            | | |
Query    YNGHTCLHLASIHGYLGIVELLVSLGADVNAQEPCNGRTALHLAVDLQNPDLVSLLLKCG
         CCCCHHHHHHHHCCCHHHHHHHHHCCCCCCCCCCCCCHHHHHHHHHCCCHHHHHHHHHCC
                190       200       210       220       230       240

              30                   40        50
         CCCCCCCC-----------CCCHHHHHHHHHCCCCCCCCCCCCC----------------
1h9eA00  VTLPAGEQ-----------RKDVYVQLYLQHLTARNRPPLPAGT----------------
                            |     |  |  ||  |   ||
Query    ADVNRVTYQGYSPYQLTWGRPSTRIQQQLGQLTLENLQMLPESEDEESYDTESEFTEFTE
         CCCCCCCCCCCHHHHHHHHCCCHHHHHHHHHCCCCCCCCCCCCCCCCCCCHHCCCCCCCC
                250       260       270       280       290       300


         -----------------
1h9eA00  -----------------

Query    DELPYDDCVFGGQRLTL
         CCCCCCCCCCCCCCCCC
                310

Percentage Identity = 23.2.



>>> Alignment with 1vg0A01:

         ------------------------------------------------------------
1vg0A01  ------------------------------------------------------------

Query    MFQAAERPQEWAMEGPRDGLKKERLLDDRHDSGLDSMKDEEYEQMVKELQEIRLEPQEVP
         CCCCCCCCHHHHHCCCCCHHHHHHHHHHHCCCCCCCCCCCHHHHHHHHHHHCCCCCCCCC
                 10        20        30        40        50        60


         ------------------------------------------------------------
1vg0A01  ------------------------------------------------------------

Query    RGSEPWKQQLTEDGDSFLHLAIIHEEKALTMEVIRQVKGDLAFLNFQNNLQQTPLHLAVI
         CCCCCCCCCCCCCCCCHHHHHHHCCHHHHHHHHHHHHHHCCCCCCCCCCCCHHHHHHHHH
                 70        80        90       100       110       120


         ------------------------------------------------------------
1vg0A01  ------------------------------------------------------------

Query    TNQPEIAEALLGAGCDPELRDFRGNTPLHLACEQGCLASVGVLTQSCTTPHLHSILKATN
         CCCHHHHHHHHHCCCCCCCCCCCCCHHHHHHHHCCCHHHHHHHHHCCCCCCCCCCCCCCC
                130       140       150       160       170       180


         ------------------------------------------------------------
1vg0A01  ------------------------------------------------------------

Query    YNGHTCLHLASIHGYLGIVELLVSLGADVNAQEPCNGRTALHLAVDLQNPDLVSLLLKCG
         CCCCHHHHHHHHCCCHHHHHHHHHCCCCCCCCCCCCCHHHHHHHHHCCCHHHHHHHHHCC
                190       200       210       220       230       240


         ------------------------------------------------------------
1vg0A01  ------------------------------------------------------------

Query    ADVNRVTYQGYSPYQLTWGRPSTRIQQQLGQLTLENLQMLPESEDEESYDTESEFTEFTE
         CCCCCCCCCCCHHHHHHHHCCCHHHHHHHHHCCCCCCCCCCCCCCCCCCCHHCCCCCCCC
                250       260       270       280       290       300

                 10           20        30        40        50
         CCCCCCCCEEEECC---CHHHHHHHHHHHHCCCCEEEECCCCCCCCCCCCCCHHHHHHHH
1vg0A01  DNLPSDFDVIVIGT---GLPESIIAAACSRSGQRVLHVDSRSYYGGNWASFSFSGLLSWL
         | ||   |  | |     |
Query    DELP--YDDCVFGGQRLTL-----------------------------------------
         CCCC--CCCCCCCCCCCCC-----------------------------------------
                  310
```

```
          60        70        80        90       100       110
         HHCCCHHHHCCCCEEEEECCCCCCCCCCCCCCHHHHHHHHHHHHHCCCEEECCCCEEEEEE
1vg0A01  KEYQMWQEQILENEEAIPLSSKDFLFPLYGQGELPQCFCRMCAVFGGIYCLRHSVQCLVV

Query    ------------------------------------------------------------
         ------------------------------------------------------------


         120       130       140       150       160       170
         ECCCCCEEEEEECCCCEEECCEEEEECCCCCCCCCCCCCCCCCCCCCCCCCCCCCEEEEC
1vg0A01  DKESRKCKAVIDQFGQRIISKHFIIEDSYLSENTCSRVQYSSDISRDCYNDLPSNVYVCS

Query    ------------------------------------------------------------
         ------------------------------------------------------------


         180       190       200
         CCCCCCCCHHHHHHHHHHHHHHHCCCCCC
1vg0A01  GPDSGLGNDNAVKQAETLFQQICPNEDF

Query    ----------------------------
         ----------------------------
```

Percentage Identity = 3.4.

```
>>> Alignment with 1k1aA00:
                 10        20        30        40        50        60
         CCCCCHHHHHHHCCCHHHHHHHHHHHHHHCCCCCCCCCCCCCHHHHHHHCCCHHHHHHHH
1k1aA00  EDGDTPLHIAVVQGNLPAVHRLVNLFQQGGRELDIYNNLRQTPLHLAVITTLPSVVRLLV

Query    ------------------------------------------------------------
         ------------------------------------------------------------


                 70        80        90       100       110       120
         HCCCCCCCCCCCCCCCHHHHHHHCCCHHHHHHHHHHCCCCCCCCCCCCCCCCHHHHHHHH
1k1aA00  TAGASPMALDRHGQTAAHLACEHRSPTCLRALLDSAAPGTLDLEARNYDGLTALHVAVNT

Query    ------------------------------------------------------------
         ------------------------------------------------------------


                130       140       150         160        170
         CCHHHHHHHHHCCCCCCCCCCCCCCHHHHHHHCC--CHHHHHHHHHC-CCCCCCCCCC
1k1aA00  ECQETVQLLLERGADIDAVDIKSGRSPLIHAVENN--SLSMVQLLLQH-GANVNAQMYSG
                                   |   | |     |    ||
Query    -------------------MFQAAERPQEWAMEGPRDGLKKERLLDDRHDSGLDSMKDEE
         -------------------CCCCCCCCHHHHHCCCCCCHHHHHHHHHHCCCCCCCCCCC
                                   10        20        30        40

         180       190       200       210       220
         CCHHHHHHHHCCHHHHHHHHHHCCCCCCCCCCCCCCCCCCCCCHHHHHHHCC
1k1aA00  SSALHSASGRGLLPLVRTLVRSGADSSLKNCHNDTPLMVARSRRVIDILRG
                     |        |
Query    YEQMVKELQEIRLEPQEVPRGSEPWKQQLTE--------------------
         HHHHHHHHHHCCCCCCCCCCCCCCCCCCCCC--------------------
                50        60        70
```

Percentage Identity = 3.5.

```
>>> Alignment with 2r6fA04:

         ------------------------------------------------------------
2r6fA04  ------------------------------------------------------------

Query    MFQAAERPQEWAMEGPRDGLKKERLLDDRHDSGLDSMKDEEYEQMVKELQEIRLEPQEVP
         CCCCCCCCHHHHHCCCCCCHHHHHHHHHHCCCCCCCCCCCCHHHHHHHHHHCCCCCCCCC
                 10        20        30        40        50        60


         ------------------------------------------------------------
2r6fA04  ------------------------------------------------------------

Query    RGSEPWKQQLTEDGDSFLHLAIIHEEKALTMEVIRQVKGDLAFLNFQNNLQQTPLHLAVI
         CCCCCCCCCCCCCCCCCHHHHHHHCCCHHHHHHHHHHHHHHCCCCCCCCCCCCCHHHHHHHH
                 70        80        90       100       110       120


         ------------------------------------------------------------
```

```
2r6fA04  ------------------------------------------------------------

Query    TNQPEIAEALLGAGCDPELRDFRGNTPLHLACEQGCLASVGVLTQSCTTPHLHSILKATN
         CCCHHHHHHHHHCCCCCCCCCCCCCCHHHHHHHCCCHHHHHHHHHCCCCCCCCCCCCCCC
                130       140       150       160       170       180


         ------------------------------------------------------------
2r6fA04  ------------------------------------------------------------

Query    YNGHTCLHLASIHGYLGIVELLVSLGADVNAQEPCNGRTALHLAVDLQNPDLVSLLLKCG
         CCCCHHHHHHHHCCCHHHHHHHHHCCCCCCCCCCCCCHHHHHHHHHCCHHHHHHHHHHCC
                190       200       210       220       230       240


         ----------------------------------------------------------CE
2r6fA04  ----------------------------------------------------------AK

Query    ADVNRVTYQGYSPYQLTWGRPSTRIQQQLGQLTLENLQMLPESEDEESYDTESEFTEFTE
         CCCCCCCCCCCHHHHHHHHCCCHHHHHHHHHCCCCCCCCCCCCCCCCCCCHHCCCCCCCC
                250       260       270       280       290       300

                 10        20        30        40        50        60
         ECC--CHHHHCCCCCCCCCCCCCCCCCCHHHHHHHHHHHHHCCCCCCCCCCHHHHHHHHH
2r6fA04  LEV--DLDLVIPNDELTLKEHAIAPWEPYYPQLLEAVCRHYGIPDVPVKDLPKEQLDKIL
          |   | | |     |||
Query    DELPYD-DCVFGGQRLTL------------------------------------------
         CCCCCC-CCCCCCCCCCC------------------------------------------
                 310

                 70        80        90       100       110
         HCCCCCCCCCCCCCCCCCCCCCCCCCCHHHHHHHHHHHCCCCCCCCCCCCCCEEC
2r6fA04  YGSGGEPIYFRYTNDFGQVREQYIAFEGVIPNVERRYRETSSDYIREQEKYAEQP

Query    -------------------------------------------------------
         -------------------------------------------------------
```

Percentage Identity = 6.1.

```
>>> Alignment with 2nvkX01:

         ------------------------------------------------------------
2nvkX01  ------------------------------------------------------------

Query    MFQAAERPQEWAMEGPRDGLKKERLLDDRHDSGLDSMKDEEYEQMVKELQEIRLEPQEVP
         CCCCCCCCHHHHHCCCCCCHHHHHHHHHCCCCCCCCCCCCHHHHHHHHHHCCCCCCCCCC
                 10        20        30        40        50        60


         ------------------------------------------------------------
2nvkX01  ------------------------------------------------------------

Query    RGSEPWKQQLTEDGDSFLHLAIIHEEKALTMEVIRQVKGDLAFLNFQNNLQQTPLHLAVI
         CCCCCCCCCCCCCCCHHHHHHHHCCCHHHHHHHHHHHHHCCCCCCCCCCCCHHHHHHHHH
                 70        80        90       100       110       120


         ------------------------------------------------------------
2nvkX01  ------------------------------------------------------------

Query    TNQPEIAEALLGAGCDPELRDFRGNTPLHLACEQGCLASVGVLTQSCTTPHLHSILKATN
         CCCHHHHHHHHHCCCCCCCCCCCCCCHHHHHHHCCCHHHHHHHHHCCCCCCCCCCCCCCC
                130       140       150       160       170       180


         ------------------------------------------------------------
2nvkX01  ------------------------------------------------------------

Query    YNGHTCLHLASIHGYLGIVELLVSLGADVNAQEPCNGRTALHLAVDLQNPDLVSLLLKCG
         CCCCHHHHHHHHCCCHHHHHHHHHCCCCCCCCCCCCCHHHHHHHHHCCHHHHHHHHHHCC
                190       200       210       220       230       240


         ------------------------------------------------------------
2nvkX01  ------------------------------------------------------------

Query    ADVNRVTYQGYSPYQLTWGRPSTRIQQQLGQLTLENLQMLPESEDEESYDTESEFTEFTE
         CCCCCCCCCCCHHHHHHHHCCCHHHHHHHHHCCCCCCCCCCCCCCCCCCCHHCCCCCCCC
                250       260       270       280       290       300
```

```
                   10        20        30        40        50
         --CCCCEEEECCCHHHHHHHHHHHHCCCCEEEECCCCCCCCCCCCCCCCCHHHHHCHHHH
2nvkX01  --YDYLIVIGGGSAGLACAKEAVLNGARVACLDFVKPTPTLGTKWGVGGTCVNVGCIPK
             ||  | ||    |
Query    DELPYDDCVFGGQRLTL-------------------------------------------
         CCCCCCCCCCCCCCCCC-------------------------------------------
                310

                   70        80        90       100       110
         HHHHHHHHHHHHHHHHHHHCCCCCCCCCCHHHHHHHHHHHHHHHHHHHHHHHHHHCCCEE
2nvkX01  KLMHQASLLGEAVHEAAAYGWNVDDKIKPDWHKLVQSVQNHIKSVNWVTRVDLRDKKVEY

Query    ------------------------------------------------------------
         ------------------------------------------------------------


                  130       140       150       160       170
         ECEEEEEEECCEEEEEECCCCEECCCCEEEECCCEECCCEECCCCCCCCCCCCCCCCCCC
2nvkX01  INGLGSFVDSHTLLAKLKSGERTITAQTFVIAVGGRPGRKGLVDDLNLPNAGVTVQKDKI

Query    ------------------------------------------------------------
         ------------------------------------------------------------


                  190       200       210       220       230
         CCCCCCCCCCCCEEECCCCCCCCCCCHHHHHHHHHHHHHHHHCCCCCCCCCC
2nvkX01  PVDSQEATNVANIYAVGDIIYGKPELTPVAVLAGRLLARRLYGGSTQRMDYK

Query    ----------------------------------------------------
         ----------------------------------------------------


Percentage Identity = 2.6.


>>> Alignment with 1iknD00:

         ------------------------------------------------------------
1iknD00  ------------------------------------------------------------

Query    MFQAAERPQEWAMEGPRDGLKKERLLDDRHDSGLDSMKDEEYEQMVKELQEIRLEPQEVP
         CCCCCCCCHHHHHCCCCCHHHHHHHHHHHCCCCCCCCCCCCHHHHHHHHHHCCCCCCCCC
                 10        20        30        40        50        60

                             10        20             30        40
         ------------CCCCCHHHHHCCCCCCCCCCCC-----CCCCCCCCCCCCCHHHHHHHH
1iknD00  ------------DGDSFLHLAIIHEEKALTMEVI-----RLAFLNFQNNLQQTPLHLAVI
                     ||||||||||||||||||||||     |||||||||||||||||||||
Query    RGSEPWKQQLTEDGDSFLHLAIIHEEKALTMEVIRQVKGDLAFLNFQNNLQQTPLHLAVI
         CCCCCCCCCCCCCCCCHHHHHHCCHHHHHHHHHHHHHHCCCCCCCCCCCCHHHHHHHHHH
                 70        80        90       100       110       120

              50        60        70        80        90       100
         CCCHHHHHCCCCCCCCCCCCCCCCCHHHHHHHHHCCHHHHHHHHCCCCCCCCCCCCCCCC
1iknD00  TNQPEIAEALLGAGCDPELRDFRGNTPLHLACEQGCLASVGVLTQSCTTPHLHSILKATN
         ||||||||||||||||||||||||||||||||||||||||||||||||||||||||||||
Query    TNQPEIAEALLGAGCDPELRDFRGNTPLHLACEQGCLASVGVLTQSCTTPHLHSILKATN
         CCCHHHHHHHHHCCCCCCCCCCCCCHHHHHHHHCCHHHHHHHHHCCCCCCCCCCCCCCCC
                130       140       150       160       170       180

             110       120       130       140       150       160
         CCCCCHHHHHHHCCCHHHHHHHHHHCCCCCCCCCCCCCHHHHHHHHCCCHHHHHHHHCCC
1iknD00  YNGHTCLHLASIHGYLGIVELLVSLGADVNAQEPCNGRTALHLAVDLQNPDLVSLLLKCG
         ||||||||||||||||||||||||||||||||||||||||||||||||||||||||||||
Query    YNGHTCLHLASIHGYLGIVELLVSLGADVNAQEPCNGRTALHLAVDLQNPDLVSLLLKCG
         CCCCHHHHHHHHCCCHHHHHHHHCCCCCCCCCCCCCCHHHHHHHHCCHHHHHHHHHHHCC
                190       200       210       220       230       240

             170       180       190       200       210
         CCCCCCCCCCCCHHHHCCCCCCHHHHHHHHCCCCCCCCCCCCCCCCCCCCCCC-------
1iknD00  ADVNRVTYQGYSPYQLTWGRPSTRIQQQLGQLTLENLQMLPESEDEESYDTE--------
         ||||||||||||||||||||||||||||||||||||||||||||||||||||
Query    ADVNRVTYQGYSPYQLTWGRPSTRIQQQLGQLTLENLQMLPESEDEESYDTESEFTEFTE
         CCCCCCCCCCCCHHHHHHHCCCHHHHHHHHHCCCCCCCCCCCCCCCCCCCCHHCCCCCCC
                250       260       270       280       290       300


         -----------------
1iknD00  -----------------
```

```
Query    DELPYDDCVFGGQRLTL
         CCCCCCCCCCCCCCCCC
                310

Percentage Identity = 99.1.

>>> Alignment with 1n0rA00:

         ------------------------------------------------------------
1n0rA00  ------------------------------------------------------------

Query    MFQAAERPQEWAMEGPRDGLKKERLLDDRHDSGLDSMKDEEYEQMVKELQEIRLEPQEVP
         CCCCCCCCHHHHHCCCCCCHHHHHHHHHHCCCCCCCCCCCHHHHHHHHHHHCCCCCCCCC
                 10        20        30        40        50        60

                                                                  10
         -----------------------------------------------CC--CCHHHHHHH
1n0rA00  -----------------------------------------------NG--RTPLHLAAR
                                                        |    ||||||
Query    RGSEPWKQQLTEDGDSFLHLAIIHEEKALTMEVIRQVKGDLAFLNFQNNLQQTPLHLAVI
         CCCCCCCCCCCCCCCCHHHHHHHCCCHHHHHHHHHHHHHCCCCCCCCCCCCCHHHHHHHH
                 70        80        90       100       110       120

                20        30        40        50        60
         HCCHHHHHHHHHHCCCCCCCCCCCCHHHHHHHHCCHHHHHHHHHHCCCCCC------CCC
1n0rA00  NGHLEVVKLLLEAGADVNAKDKNGRTPLHLAARNGHLEVVKLLLEAGADVN------AKD
             |    || || |    |  | ||||||   | |  |  |              |
Query    TNQPEIAEALLGAGCDPELRDFRGNTPLHLACEQGCLASVGVLTQSCTTPHLHSILKATN
         CCCHHHHHHHHHCCCCCCCCCCCCCHHHHHHHHCCHHHHHHHHHHCCCCCCCCCCCCCCC
                130       140       150       160       170       180

            70        80        90        100       110       120
         CCCCHHHHHHHHCCHHHHHHHHHHCCCCCCCC-CCCCCCHHHHHHHCCHHHHHHHHHHHC
1n0rA00  KNGRTPLHLAARNGHLEVVKLLLEAGADVNAK-DKNGRTPLHLAARNGHLEVVKLLLEAG
          || | ||||   | |  | ||   ||||||    |||| ||||        | |||  |
Query    YNGHTCLHLASIHGYLGIVELLVSLGADVNAQEPCNGRTALHLAVDLQNPDLVSLLLKCG
         CCCCHHHHHHHHCCCHHHHHHHHHCCCCCCCCCCCCCHHHHHHHHHCCHHHHHHHHHHCC
                190       200       210       220       230       240

         CC----------------------------------------------------------
1n0rA00  AY----------------------------------------------------------
         |
Query    ADVNRVTYQGYSPYQLTWGRPSTRIQQQLGQLTLENLQMLPESEDEESYDTESEFTEFTE
         CCCCCCCCCCCCHHHHHHHCCCHHHHHHHHHCCCCCCCCCCCCCCCCCCCCHHCCCCCCC
                250       260       270       280       290       300

         -----------------
1n0rA00  -----------------

Query    DELPYDDCVFGGQRLTL
         CCCCCCCCCCCCCCCCC
                310

Percentage Identity = 46.0.

>>> Alignment with 1n0qB00:

         ------------------------------------------------------------
1n0qB00  ------------------------------------------------------------

Query    MFQAAERPQEWAMEGPRDGLKKERLLDDRHDSGLDSMKDEEYEQMVKELQEIRLEPQEVP
         CCCCCCCCHHHHHCCCCCCHHHHHHHHHHCCCCCCCCCCCHHHHHHHHHHHCCCCCCCCC
                 10        20        30        40        50        60

         ------------------------------------------------------------
1n0qB00  ------------------------------------------------------------

Query    RGSEPWKQQLTEDGDSFLHLAIIHEEKALTMEVIRQVKGDLAFLNFQNNLQQTPLHLAVI
         CCCCCCCCCCCCCCCCHHHHHHHCCCHHHHHHHHHHHHHCCCCCCCCCCCCCHHHHHHHH
                 70        80        90       100       110       120

                                       10        20              30
         --------------------C--CCCHHHHHHHCCCHHHHHHHHHCCCCCC------CCC
1n0qB00  --------------------N--GRTPLHLAARNGHLEVVKLLLEAGADVN------AKD
                                | ||||||   | |  |  |              |
Query    TNQPEIAEALLGAGCDPELRDFRGNTPLHLACEQGCLASVGVLTQSCTTPHLHSILKATN
```

```
         CCCHHHHHHHHHCCCCCCCCCCCCCCHHHHHHHCCHHHHHHHHHCCCCCCCCCCCCCC
                130       140       150       160       170       180


               40        50        60         70        80        90
         CCCCCHHHHHHHCCHHHHHHHHHHCCCCCCCC-CCCCCCHHHHHHHCCHHHHHHHHHHCC
1n0qB00  KNGRTPLHLAARNGHLEVVKLLLEAGADVNAK-DKNGRTPLHLAARNGHLEVVKLLLEAG
          || | ||||   | |  | ||   ||||||    |||| ||||        | |||  |
Query    YNGHTCLHLASIHGYLGIVELLVSLGADVNAQEPCNGRTALHLAVDLQNPDLVSLLLKCG
         CCCCHHHHHHHHCCHHHHHHHHHHCCCCCCCCCCCCHHHHHHHHCCHHHHHHHHHHCC
                190       200       210       220       230       240


         CC----------------------------------------------------------
1n0qB00  AY----------------------------------------------------------
         |
Query    ADVNRVTYQGYSPYQLTWGRPSTRIQQQLGQLTLENLQMLPESEDEESYDTESEFTEFTE
         CCCCCCCCCCCCHHHHHHHCCCHHHHHHHHHHCCCCCCCCCCCCCCCCCCCCHHCCCCCCC
                250       260       270       280       290       300


         -----------------
1n0qB00  -----------------

Query    DELPYDDCVFGGQRLTL
         CCCCCCCCCCCCCCCCC
                310

Percentage Identity = 47.3.

>>> Alignment with 1n11A00:
                 10        20        30        40        50        60
         CCHHHHHHHHCCHHHHHHHHHHCCCCCCCCCCCCCCHHHHHHHHCCHHHHHHHHHHHCCCCC
1n11A00  LTPLHVASFMGHLPIVKNLLQRGASPNVSNVKVETPLHMAARAGHTEVAKYLLQNKAKVN

Query    ------------------------------------------------------------
         ------------------------------------------------------------


                 70        80        90       100       110       120
         CCCCCCCCHHHHHHHHCCHHHHHHHHHHCCCCCCCCCCCCCHHHHHHHHCCHHHHHHHHH
1n11A00  AKAKDDQTPLHCAARIGHTNMVKLLLENNANPNLATTAGHTPLHIAAREGHVETVLALLE

Query    ------------------------------------------------------------
         ------------------------------------------------------------


                130       140          150       160       170
         CCCCCCCCCCCCCCHHHHHHHCCC---HHHHHHHHHCCCCCCCCCCCCCHHHHHHHHCCC
1n11A00  KEASQACMTKKGFTPLHVAAKYGK---VRVAELLLERDAHPNAAGKNGLTPLHVAVHHNN
                |      |   |             |    |
Query    -------MFQAAERPQEWAMEGPRDGLKKERLLDDRHDSGLDSMKDEEYEQMVKELQEIR
         -------CCCCCCCCHHHHHCCCCCCHHHHHHHHHHHCCCCCCCCCCCCHHHHHHHHHHHCC
                        10        20        30        40        50

         180       190       200       210           220       230
         HHHHHHHHHHCCCCCCCCCCCCCHHHHHHHHCCC----HHHHHHHHHCCCCCCCCCCCCCC
1n11A00  LDIVKLLLPRGGSPHSPAWNGYTPLHIAAKQNQ----VEVARSLLQYGGSANAESVQGVT
         |                   |   || |          || |         |       |
Query    LEPQEVPRGSEPWKQQLTEDGDSFLHLAIIHEEKALTMEVIRQVKGDLAFLNFQNNLQQT
         CCCCCCCCCCCCCCCCCCCCCCCCCHHHHHHHCCHHHHHHHHHHHHHHCCCCCCCCCCCCCH
              60        70        80        90       100       110

             240       250       260       270       280       290
         HHHHHHHCCCHHHHHHHHHCCCCCCCCCCCCCCCHHHHHHHHCCHHHHHHHHHHHCCCCC--
1n11A00  PLHLAAQEGHAEMVALLLSKQANGNLGNKSGLTPLHLVAQEGHVPVADVLIKHGVMVD--
         |||||      |    ||       |    | |||||    |      ||
Query    PLHLAVITNQPEIAEALLGAGCDPELRDFRGNTPLHLACEQGCLASVGVLTQSCTTPHLH
         HHHHHHHCCCHHHHHHHHHHCCCCCCCCCCCCCCCHHHHHHHHCCHHHHHHHHHHCCCCCCCC
             120       130       140       150       160       170

                   300       310       320        330       340
         ----CCCCCCCCHHHHHHHCCCCHHHHHHHHHCCCCCCCCCC-CCCCHHHHHHHHCCCHHHH
1n11A00  ----ATTRMGYTPLHVASHYGNIKLVKFLLQHQADVNAKTK-LGYSPLHQAAQQGHTDIV
             ||   | | || ||  |    |  |    |||||     |   || |      | |
Query    SILKATNYNGHTCLHLASIHGYLGIVELLVSLGADVNAQEPCNGRTALHLAVDLQNPDLV
         CCCCCCCCCCCHHHHHHHHHCCCHHHHHHHHHHCCCCCCCCCCCCCHHHHHHHHHCCCHHHH
             180       190       200       210       220       230

          350       360       370       380       390       400
         HHHHHCCCCCCCCCCCCCCCHHHHHHHHCCCHHHHHHHHHHHCCCCCCCCCCCCCCCCCCCC--
```

```
1n11A00  TLLLKNGASPNEVSSDGTTPLAIAKRLGYISVTDVLKVVTDETSFVLHRMSFPETVDE--
          |||| ||  | |   |  |               |   | |    |     ||  ||
Query    SLLLKCGADVNRVTYQGYSPYQLTWGRPSTRIQQQLGQLTLENLQML-----PESEDEES
         HHHHHCCCCCCCCCCCCCCHHHHHHHCCCHHHHHHHHCCCCCCCCCC-----CCCCCCCC
             240       250       260       270       280


         -----------------------------
1n11A00  -----------------------------

Query    YDTESEFTEFTEDELPYDDCVFGGQRLTL
         CCCHHCCCCCCCCCCCCCCCCCCCCCCCC
                  300       310

Percentage Identity = 23.0.

>>> Alignment with 1awcB00:

         ------------------------------------------------------------
1awcB00  ------------------------------------------------------------

Query    MFQAAERPQEWAMEGPRDGLKKERLLDDRHDSGLDSMKDEEYEQMVKELQEIRLEPQEVP
         CCCCCCCHHHHHCCCCCCHHHHHHHHHHHCCCCCCCCCCCCHHHHHHHHHHCCCCCCCCC
                 10        20        30        40        50        60


                                                                  10
         -----------------------------------------------C--HHHHHHHHHH
1awcB00  -----------------------------------------------D--LGKKLLEAAR
                                                               |  |
Query    RGSEPWKQQLTEDGDSFLHLAIIHEEKALTMEVIRQVKGDLAFLNFQNNLQQTPLHLAVI
         CCCCCCCCCCCCCCCCHHHHHHHCCHHHHHHHHHHHHHHCCCCCCCCCCCCHHHHHHHHH
                 70        80        90       100       110       120

                20         30        40        50        60
         HCCHHHHHHHHHHCCCCC-CCCCCCHHHHHHHHHCCHHHHHHHHCCCCCCC------CCC
1awcB00  AGQDDEVRILMANGAPFT-TDWLGTSPLHLAAQYGHFSTTEVLLRAGVSRD------ART
           |      |   |      |  |  |||||   |      ||              |
Query    TNQPEIAEALLGAGCDPELRDFRGNTPLHLACEQGCLASVGVLTQSCTTPHLHSILKATN
         CCCHHHHHHHHHCCCCCCCCCCCCCHHHHHHHCCHHHHHHHHHHHCCCCCCCCCCCCCCC
                130       140       150       160       170       180

             70        80        90       100        110       120
         CCCCHHHHHHHHHCCHHHHHHHHCCCCCCCCCCCCC-CCHHHHHHHCCCHHHHHHHHHCC
1awcB00  KVDRTPLHMAASEGHANIVEVLLKHGADVNAKDMLK-MTALHWATEHNHQEVVELLIKYG
             | || |   |   ||| |   ||||||       |||| |        | || | |
Query    YNGHTCLHLASIHGYLGIVELLVSLGADVNAQEPCNGRTALHLAVDLQNPDLVSLLLKCG
         CCCCHHHHHHHHCCCHHHHHHHHHCCCCCCCCCCCCCHHHHHHHHHCCCHHHHHHHHHCC
                190       200       210       220       230       240

             130       140       150
         CCCCCCCCCCCCHHHHHHHCCCHHHHHHHC------------------------------
1awcB00  ADVHTQSKFCKTAFDISIDNGNEDLAEILQ------------------------------
         |||                         |
Query    ADVNRVTYQGYSPYQLTWGRPSTRIQQQLGQLTLENLQMLPESEDEESYDTESEFTEFTE
         CCCCCCCCCCCCHHHHHHHCCCHHHHHHHHCCCCCCCCCCCCCCCCCCCCCHHCCCCCCC
                250       260       270       280       290       300


         -----------------
1awcB00  -----------------

Query    DELPYDDCVFGGQRLTL
         CCCCCCCCCCCCCCCCC
                310

Percentage Identity = 29.4.

>>> Alignment with 1k1aA00:

         ------------------------------------------------------------
1k1aA00  ------------------------------------------------------------

Query    MFQAAERPQEWAMEGPRDGLKKERLLDDRHDSGLDSMKDEEYEQMVKELQEIRLEPQEVP
         CCCCCCCHHHHHCCCCCCHHHHHHHHHHHCCCCCCCCCCCCHHHHHHHHHHCCCCCCCCC
                 10        20        30        40        50        60

                            10        20        30        40
         -----------CCCCCHHHHHHHCCHHHHHHHHHHHHHHCCCCCCCCCCCCHHHHHHHHH
1k1aA00  -----------EDGDTPLHIAVVQGNLPAVHRLVNLFQQGGRELDIYNNLRQTPLHLAVI
                    ||||  || |                      |   ||| |||||||||
```

```
Query    RGSEPWKQQLTEDGDSFLHLAIIHEEKALTMEVIRQVKGDLAFLNFQNNLQQTPLHLAVI
         CCCCCCCCCCCCCCCCHHHHHHHCCCHHHHHHHHHHHHHHCCCCCCCCCCCCCHHHHHHHH
                 70        80        90       100       110       120


                  60        70        80        90       100
         CCCHHHHHHHHHHCCCCCCCCCCCCCCCHHHHHHHCCCHHHHHHHHHHCCCCCCCC--CCCC
1k1aA00  TTLPSVVRLLVTAGASPMALDRHGQTAAHLACEHRSPTCLRALLDSAAPGTLDL--EARN
         |  |     |  ||  |   |  | |  |||||         |  |     |     | |
Query    TNQPEIAEALLGAGCDPELRDFRGNTPLHLACEQGCLASVGVLTQSCTTPHLHSILKATN
         CCCHHHHHHHHHHCCCCCCCCCCCCCCCHHHHHHHCCCHHHHHHHHHHCCCCCCCCCCCCCC
                130       140       150       160       170       180


         110       120       130       140       150       160
         CCCCCHHHHHHHHHCCHHHHHHHHHHCCCCCCCCCCCCCCHHHHHHHCCCHHHHHHHHHHCC
1k1aA00  YDGLTALHVAVNTECQETVQLLLERGADIDAVDIKSGRSPLIHAVENNSLSMVQLLLQHG
         | | | || |        | ||   |||  |     ||  |  ||       | |||  |
Query    YNGHTCLHLASIHGYLGIVELLVSLGADVNAQEPCNGRTALHLAVDLQNPDLVSLLLKCG
         CCCCHHHHHHHHHCCCHHHHHHHHHHCCCCCCCCCCCCCCHHHHHHHHCCCHHHHHHHHHHCC
                190       200       210       220       230       240


         170       180       190       200
         CCCCCCCCCCCCHHHHHHHHHCCHHHHHHHHHHCC-----CCCCCCCCC-------------
1k1aA00  ANVNAQMYSGSSALHSASGRGLLPLVRTLVRSG-----ADSSLKNCH-------------
         | ||   | | |      ||        |
Query    ADVNRVTYQGYSPYQLTWGRPSTRIQQQLGQLTLENLQMLPESEDEESYDTESEFTEFTE
         CCCCCCCCCCCCHHHHHHHCCCHHHHHHHHHHCCCCCCCCCCCCCCCCCCCHHCCCCCCC
                250       260       270       280       290       300


                 210       220
         ----------CCCCCCCCCCHHHHHHHCC
1k1aA00  ----------NDTPLMVARSRRVIDILRG
                       |
Query    DELPYDDCVFGGQRLTL------------
         CCCCCCCCCCCCCCCCC------------
                310

Percentage Identity = 31.6.

>>> Alignment with 1ympA00:

         ------------------------------------------------------------
1ympA00  ------------------------------------------------------------

Query    MFQAAERPQEWAMEGPRDGLKKERLLDDRHDSGLDSMKDEEYEQMVKELQEIRLEPQEVP
         CCCCCCCCHHHHHCCCCCCHHHHHHHHHHHCCCCCCCCCCCCHHHHHHHHHHHCCCCCCCCC
                 10        20        30        40        50        60


                                                        10        20
         ---------------------------------------CCCCCCCCCCCCCCHHHHHHH
1ympA00  ---------------------------------------RATDLDARMHDGTTPLILAAR
                                                   |        ||| ||
Query    RGSEPWKQQLTEDGDSFLHLAIIHEEKALTMEVIRQVKGDLAFLNFQNNLQQTPLHLAVI
         CCCCCCCCCCCCCCCCHHHHHHHCCCHHHHHHHHHHHHHHCCCCCCCCCCCCCHHHHHHHH
                 70        80        90       100       110       120


                30        40        50        60        70
         HCCCCHHHHHHHCCCCCCCCCCCCCCHHHHHHHCCCHHHHHHHHHCCCCCCCC------C
1ympA00  LALEGMLEDLINSHADVNAVDDLGKSALHWAAAVNNVDAAVVLLKNGANKDMQ------N
                | |     |    |  |   || |          ||                |
Query    TNQPEIAEALLGAGCDPELRDFRGNTPLHLACEQGCLASVGVLTQSCTTPHLHSILKATN
         CCCHHHHHHHHHHCCCCCCCCCCCCCCCHHHHHHHCCCHHHHHHHHHHCCCCCCCCCCCCCC
                130       140       150       160       170       180


            80        90       100        110       120       130
         CCCCCHHHHHHHHCCHHHHHHHHHHCCCCCCC-CCCCCCHHHHHHHCCCHHHHHHHC---
1ympA00  NKEETPLFLAAREGSYETAKVLLDHFANRDIT-DHMDRLPRDIAQERMHHDIVRLLD---
             | | ||   |       |    |          |     |      | | ||
Query    YNGHTCLHLASIHGYLGIVELLVSLGADVNAQEPCNGRTALHLAVDLQNPDLVSLLLKCG
         CCCCHHHHHHHHHCCCHHHHHHHHHHCCCCCCCCCCCCCCHHHHHHHHCCCHHHHHHHHHHCC
                190       200       210       220       230       240


         ------------------------------------------------------------
1ympA00  ------------------------------------------------------------

Query    ADVNRVTYQGYSPYQLTWGRPSTRIQQQLGQLTLENLQMLPESEDEESYDTESEFTEFTE
         CCCCCCCCCCCCHHHHHHHCCCHHHHHHHHHHCCCCCCCCCCCCCCCCCCCHHCCCCCCC
                250       260       270       280       290       300
```

```
         -----------------
1ympA00  -----------------

Query    DELPYDDCVFGGQRLTL
         CCCCCCCCCCCCCCCCC
                310
```

```
Percentage Identity = 22.9.
```

```
>>> Alignment with 1wdyA00:

         ------------------------------------------------------------
1wdyA00  ------------------------------------------------------------

Query    MFQAAERPQEWAMEGPRDGLKKERLLDDRHDSGLDSMKDEEYEQMVKELQEIRLEPQEVP
         CCCCCCCCHHHHHCCCCCCHHHHHHHHHHCCCCCCCCCCCCHHHHHHHHHHCCCCCCCCC
                 10        20        30        40        50        60


                             10            20        30        40
         ------------CHHHHHHHHHHHHHCCC----HHHHHHHHHCCCCCCCCCCCCCCCHHH
1wdyA00  ------------AAVEDNHLLIKAVQNED----VDLVQQLLEGGANVNFQEEEGGWTPLH
                             |  |   |          |     |  |||      ||||
Query    RGSEPWKQQLTED---GDSFLHLAIIHEEKALTMEVIRQVKGDLAFLNFQ-NNLQQTPLH
         CCCCCCCCCCCCC---CCCHHHHHHHCCCHHHHHHHHHHHHHCCCCCCCC-CCCCCHHHH
                 70           80        90       100        110


             50        60        70        80        90
         HHHHCCCHHHHHHHHHCCCCCCCCCCCCCHHHHHHHHHCCHHHHHHHHHCCCCCC-----
1wdyA00  NAVQMSREDIVELLLRHGADPVLRKKNGATPFLLAAIAGSVKLLKLFLSKGADVN-----
          ||      | | ||  | || ||   | ||  ||   |
Query    LAVITNQPEIAEALLGAGCDPELRDFRGNTPLHLACEQGCLASVGVLTQSCTTPHLHSIL
         HHHHCCCHHHHHHHHHCCCCCCCCCCCCCHHHHHHHHCCHHHHHHHHHHCCCCCCCCCCC
          120       130       140       150       160       170


                  110       120       130       140       150
         -CCCCCCCCHHHHHHHCCCHHHHHHHHHCCCCCCCCCCCHHHHHCCCCCCCHHHHHHHHH
1wdyA00  -ECDFYGFTAFMEAAVYGKVKALKFLYKRGANVNLRRKTKEDQERLRKGGATALMDAAEK
               | |    |   |       |   || ||
Query    KATNYNGHTCLHLASIHGYLGIVELLVSLGADVNAQEPC---------------------
         CCCCCCCCHHHHHHHHCCCHHHHHHHHHCCCCCCCCCCC---------------------
          180       190       200       210


                  170       180       190       200       210
         CCHHHHHHHHHCCCCCCCCCCCCCCHHHHHHHCCCCCCHHHHHHHHHHHCCCCCCCCCCC
1wdyA00  GHVEVLKILLDEMGADVNACDNMGRNALIHALLSSDDSDVEAITHLLLDHGADVNVRGER

Query    -----------------------------------------------------------N
         -----------------------------------------------------------C


                  230       240       250       260       270
         CCCHHHHHHHCCCHHHHHHHHHCCCCCCCCCCCCCCHHHHHHHHCCCHHHHHHHHHHCCC
1wdyA00  GKTPLILAVEKKHLGLVQRLLEQEHIEINDTDSDGKTALLLAVELKLKKIAELLCKRGAS
         | | | |||      ||  ||       |     |     |        |   |
Query    GRTALHLAVDLQNPDLVSLLLKC-GADVNRVTYQGYSPYQLTWGRPSTRIQQQLGQLTLE
         CCHHHHHHHHCCCHHHHHHHHHC-CCCCCCCCCCCCHHHHHHHHCCCHHHHHHHHHCCCC
          220       230        240       250       260       270


             280
         -----CCCCCCC------------------------------
1wdyA00  -----TDCGDLV------------------------------
                  |
Query    NLQMLPESEDEESYDTESEFTEFTEDELPYDDCVFGGQRLTL
         CCCCCCCCCCCCCCCCHHCCCCCCCCCCCCCCCCCCCCCCCC
           280       290       300       310
```

```
Percentage Identity = 18.9.
```

```
>>> Alignment with 2bkgA00:

         ------------------------------------------------------------
2bkgA00  ------------------------------------------------------------

Query    MFQAAERPQEWAMEGPRDGLKKERLLDDRHDSGLDSMKDEEYEQMVKELQEIRLEPQEVP
         CCCCCCCCHHHHHCCCCCCHHHHHHHHHHCCCCCCCCCCCCHHHHHHHHHHCCCCCCCCC
                 10        20        30        40        50        60


         ---------------------------------------------------CHHHHHHHH
2bkgA00  ---------------------------------------------------SDLGKKLLE
```

```
                                                                  |
Query    RGSEPWKQQLTEDGDSFLHLAIIHEEKALTMEVIRQVKGDLAFLNFQNNLQQ---TPLHL
         CCCCCCCCCCCCCCCCHHHHHHCCHHHHHHHHHHHHHHHCCCCCCCCCCCCC---HHHHH
                 70        80        90       100       110


                  20        30        40        50        60
         HHHHCCHHHHHHHHHHCCCCCCCCCCCCHHHHHHHHHCCHHHHHHHHHCCCCCCCC----
2bkgA00  AARAGQDDEVRILMANGADVNAEDTYGDTPLHLAARVGHLEIVEVLLKNGADVNAL----
         |    |      |   | |    |  | ||||||   | |  | ||
Query    AVITNQPEIAEALLGAGCDPELRDFRGNTPLHLACEQGCLASVGVLTQSCTTPHLHSILK
         HHHCCCHHHHHHHHHCCCCCCCCCCCCCHHHHHHHHCCHHHHHHHHHHCCCCCCCCCCCC
         120       130       140       150       160       170


              70        80        90        100       110       120
         --CCCCCCHHHHHHHCCCHHHHHHHHHCCCCCCC-CCCCCCCHHHHHHHCCCHHHHHHHH
2bkgA00  --DFSGSTPLHLAAKRGHLEIVEVLLKYGADVNA-DDTIGSTPLHLAADTGHLEIVEVLL
              | | ||||   | | ||| |   ||||||     | | |||| |      |  ||
Query    ATNYNGHTCLHLASIHGYLGIVELLVSLGADVNAQEPCNGRTALHLAVDLQNPDLVSLLL
         CCCCCCCHHHHHHHHCCCHHHHHHHHHCCCCCCCCCCCCCCHHHHHHHHCCCHHHHHHHH
         180       190       200       210       220       230


              130       140       150
         HCCCCCCCCCCCCCCHHHHHHHCCCCCHHHHCC---------------------------
2bkgA00  KYGADVNAQDKFGKTAFDISIDNGN-DLAEIL----------------------------
         | |||||     |                  |
Query    KCGADVNRVTYQGYSPYQLTWGRPSTRIQQQLGQLTLENLQMLPESEDEESYDTESEFTE
         HCCCCCCCCCCCCCCHHHHHHHCCCHHHHHHHHHCCCCCCCCCCCCCCCCCCCCHHCCCC
         240       250       260       270       280       290


         --------------------
2bkgA00  --------------------

Query    FTEDELPYDDCVFGGQRLTL
         CCCCCCCCCCCCCCCCCCCC
         300       310

Percentage Identity = 35.5.

>>> Alignment with 1uohA00:
                                                    10        20
         -----------------------------------CCCCHHHHHHHHCCHHHHHHHHHHH
1uohA00  -----------------------------------CVSNLMVCNLAYSGKLEELKESILA
                                                            |
Query    MFQAAERPQEWAMEGPRDGLKKERLLDDRHDSGLDSMKD---------EEYEQMVKELQE
         CCCCCCCCHHHHHCCCCCHHHHHHHHHHHCCCCCCCCCC---------CCHHHHHHHHHH
                 10        20        30                 40        50


            30                   40            50        60        70
         CHHHHHC-----------CCCCCCHHHHHHHHHCC----HHHHHHHHHHCCCCCCCCCCC
1uohA00  DKSLATR-----------TDQDSRTALHWACSAGH----TEIVEFLLQLGVPVNDKDDAG
                              |    || |          |            |
Query    IRLEPQEVPRGSEPWKQQLTEDGDSFLHLAIIHEEKALTMEVIRQVKGDLAFLNFQNNLQ
         CCCCCCCCCCCCCCCCCCCCCCCCCHHHHHHCCHHHHHHHHHHHHHHHCCCCCCCCCCCC
                60        70        80        90       100       110


                 80        90       100       110       120       130
         CCHHHHHHHHHCCHHHHHHHHCCCCCCCCCCCCCHHHHHHHHHCHHHHHHHHHCCCCCCC
1uohA00  WSPLHIAASAGRDEIVKALLGKGAQVNAVNQNGCTPLHYAASKNRHEIAVMLLEGGANPD
           ||| |      ||  |||| |         | |||| |           |      |
Query    QTPLHLAVITNQPEIAEALLGAGCDPELRDFRGNTPLHLACEQGCLASVGVLTQSCTTPH
         CHHHHHHHHCCCHHHHHHHHHCCCCCCCCCCCCCHHHHHHHHCCHHHHHHHHHHCCCCCC
               120       130       140       150       160       170


                      140       150       160        170       180
         ------CCCCCCCHHHHHHHHCCHHHHHHHHHHCCCCCCCC-CCCCCCHHHHHHHCCCHH
1uohA00  ------AKDHYEATAMHRAAAKGNLKMIHILLYYKASTNIQ-DTEGNTPLHLACDEERVE
               |      |  | |   | |     |    |  | |    | | |||| |
Query    LHSILKATNYNGHTCLHLASIHGYLGIVELLVSLGADVNAQEPCNGRTALHLAVDLQNPD
         CCCCCCCCCCCCCHHHHHHHHCCCHHHHHHHHHCCCCCCCCCCCCCCHHHHHHHHCCCHH
               180       190       200       210       220       230


             190       200       210       220
         HHHHHHHCCCCCCCCCCCCCHHHHCCCCHHHHHHHHHHCC--------------------
1uohA00  EAKLLVSQGASIYIENKEEKTPLQVAKGGLGLILKRMVEG--------------------
            ||   ||           | |   |
Query    LVSLLLKCGADVNRVTYQGYSPYQLTWGRPSTRIQQQLGQLTLENLQMLPESEDEESYDT
         HHHHHHHCCCCCCCCCCCCCCHHHHHHHCCCHHHHHHHHHCCCCCCCCCCCCCCCCCCCC
               240       250       260       270       280       290
```

```
         --------------------------
1uohA00  --------------------------

Query    ESEFTEFTEDELPYDDCVFGGQRLTL
         HHCCCCCCCCCCCCCCCCCCCCCCCC
               300       310
```

Percentage Identity = 22.4.

>>> Alignment with 2f8yB00:

```
         ------------------------------------------------------C--CCC
2f8yB00  ------------------------------------------------------A--VIS

Query    MFQAAERPQEWAMEGPRDGLKKERLLDDRHDSGLDSMKDEEYEQMVKELQEIRLEPQEVP
         CCCCCCCCHHHHHCCCCCCHHHHHHHHHHHCCCCCCCCCCCCHHHHHHHHHHCCCCCCCC
                 10        20        30        40        50        60


             10         20        30            40        50
         HHHHCCCC-CCCCCCCCCCCHHHHHHHCCC----HHHHHHHHHCCCCCCCCCCCCCCHHH
2f8yB00  DFIYQGAS-LHNQTDRTGETALHLAARYSR----SDAAKRLLEASADANIQDNMGRTPLH
              |      |    |   ||||                    |  | | |   ||||
Query    ----RGSEPWKQQLTEDGDSFLHLAIIHEEKALTMEVIRQVKGDLAFLNFQNNLQQTPLH
         ----CCCCCCCCCCCCCCCCHHHHHHHCCHHHHHHHHHHHHHCCCCCCCCCCCCCCHHHH
                     70        80        90       100       110


                  70        80        90       100       110
         HHHHHCCHHHHHHHHCCCCCCCCCCCCCCCHHHHHHHHCCHHHHHHHHHHCCCCCC----
2f8yB00  AAVSADAQGVFQILIRNRATDLDARMHDGTTPLILAARLAVEGMLEDLINSHADVN----
          ||          |      |   |   | ||| ||           |  |
Query    LAVITNQPEIAEALLGAGC-DPELRDFRGNTPLHLACEQGCLASVGVLTQSCTTPHLHSI
         HHHHCCCHHHHHHHHHCCC-CCCCCCCCCCCHHHHHHHCCHHHHHHHHHHCCCCCCCCCC
          120       130        140       150       160       170


             120       130       140       150        160       170
         --CCCCCCCHHHHHHHHCCCHHHHHHHHHCCCCCCCC-CCCCCCHHHHHHHHCCHHHHHH
2f8yB00  --AVDDLGKSALHWAAAVNNVDAAVVLLKNGANKDMQ-NNREETPLFLAAREGSYETAKV
           |    |   || |           |   ||    |      | |  ||
Query    LKATNYNGHTCLHLASIHGYLGIVELLVSLGADVNAQEPCNGRTALHLAVDLQNPDLVSL
         CCCCCCCCCHHHHHHHHCCCHHHHHHHHHCCCCCCCCCCCCCCHHHHHHHHCCCHHHHHH
           180       190       200       210       220       230


              180       190       200
         HHHCCCCCCCCCCCCCCHHHHHHHCCHHHHHHHHHHC-----------------------
2f8yB00  LLDHFANRDITDHMDRLPRDIAQERMHHDIVRLLDEY-----------------------
         ||   |           |      |    |   |
Query    LLKCGADVNRVTYQGYSPYQLTWGRPSTRIQQQLGQLTLENLQMLPESEDEESYDTESEF
         HHHCCCCCCCCCCCCCCHHHHHHHCCHHHHHHHHHHCCCCCCCCCCCCCCCCCCCCHHCC
           240       250       260       270       280       290


         ----------------------
2f8yB00  ----------------------

Query    TEFTEDELPYDDCVFGGQRLTL
         CCCCCCCCCCCCCCCCCCCCCC
           300       310
```

Percentage Identity = 23.0.

>>> Alignment with 2dznA00:

```
                                                   10        20
         ----------------------------------CCHHHHHHHCCCHHHHHHHHHHC---
2dznA00  ----------------------------------NYPLHQACMENEFFKVQELLHSK---
                                                      |       |
Query    MFQAAERPQEWAMEGPRDGLKKERLLDDRHDSGLD-----SMKDEEYEQMVKELQEIRLE
         CCCCCCCCHHHHHCCCCCHHHHHHHHHHHCCCCCC-----CCCCCHHHHHHHHHHHCCCC
                 10        20        30             40        50


                      30        40            50        60        70
         --------CCCCCCCCCCCCCHHHHHHHCCC----HHHHHHHHHCCCCCCCCCCCCCCCC
2dznA00  --------PSLLLQKDQDGRIPLHWSVSFQA----HEITSFLLSKMENVNLDDYPDDSGW
                      |   ||   ||            |            |
Query    PQEVPRGSEPWKQQLTEDGDSFLHLAIIHEEKALTMEVIRQVKGDLAFLNF---QNNLQQ
         CCCCCCCCCCCCCCCCCCCCCHHHHHHHCCCHHHHHHHHHHHHHCCCCCCC---CCCCCC
            60        70        80        90       100          110


                80        90       100       110       120       130
         CHHHHHHHHCCHHHHHHHHCCCCCCCCCCCCCCCCCHHHHHHHCCHHHHHHHHHHCCCCC
```

```
2dznA00  TPFHIACSVGNLEVVKSLYDRPLKPDLNKITNQGVTCLHLAVGKKWFEVSQFLIENGASV
         || | |      |    |       |       | | ||||           |
Query    TPLHLAVITNQPEIAEALLGA--GCDPELRDFRGNTPLHLACEQGCLASVGVLTQSCTTP
         HHHHHHHHCCCHHHHHHHHHC--CCCCCCCCCCCCCHHHHHHHCCCHHHHHHHHHCCCCC
              120       130         140       150       160       170


                     140       150       160       170       180
         C------CCCCCCCCHHHHHHHCCCHHHHHHHHCCCCCCCCCCCCCCCCHHHHHHHCCCH
2dznA00  R------IKDKFNQIPLHRAASVGSLKLIELLCGLGKSAVNWQDKQGWTPLFHALAEGHG
                         || |   | |   |||  ||          | | |  |
Query    HLHSILKATNYNGHTCLHLASIHGYLGIVELLVSLGADVNAQEPCNGRTALHLAVDLQNP
         CCCCCCCCCCCCCCHHHHHHHHCCCHHHHHHHHHCCCCCCCCCCCCCHHHHHHHHHCCCH
                180       190       200       210       220       230


           190       200       210           220
         HHHHHHHHHHCCCCCCCCCCCCHHHHHCCCCC----HHHHHHCCC---------------
2dznA00  DAAVLLVEKYGAEYDLVDNKGAKAEDVALNEQ----VKKFFLNNV---------------
         |   ||    ||    |   |
Query    DLVSLLLK-CGADVNRVTYQGYSPYQLTWGRPSTRIQQQLGQLTLENLQMLPESEDEESY
         HHHHHHHH-CCCCCCCCCCCCCCHHHHHHHCCCHHHHHHHHHCCCCCCCCCCCCCCCCCC
                 240       250       260       270       280


         ----------------------------
2dznA00  ----------------------------

Query    DTESEFTEFTEDELPYDDCVFGGQRLTL
         CCHHCCCCCCCCCCCCCCCCCCCCCCCC
                 300       310

Percentage Identity = 19.5.

>>> Alignment with 1bd8A00:

         ------------------------------------------------------------
1bd8A00  ------------------------------------------------------------

Query    MFQAAERPQEWAMEGPRDGLKKERLLDDRHDSGLDSMKDEEYEQMVKELQEIRLEPQEVP
         CCCCCCCCHHHHHCCCCCCHHHHHHHHHHCCCCCCCCCCCHHHHHHHHHHHCCCCCCCCC
                 10        20        30        40        50        60


                             10            20        30        40
         ------------CHHHHHHHHHHHCC----HHHHHHHHHCCCCCCCCCCCCCCHHHHHC-
1bd8A00  ------------RAGDRLSGAAARGD----VQEVRRLLHRELVHPDALNRFGKTALQVM-
                          |  |             |      |      |    | |
Query    RGSEPWKQQLTEDGDSFLHLAIIHEEKALTMEVIRQVKGD-LAFLNFQNNLQQTPLHLAV
         CCCCCCCCCCCCCCCCHHHHHHHCCHHHHHHHHHHHHHHC-CCCCCCCCCCCHHHHHHHH
                 70        80        90      100        110


              50        60        70        80        90
         CCCCHHHHHHHHHCCCCCCCCCCCCCHHHHHHHHCCHHHHHHHHHHCCCCCCCC------
1bd8A00  MFGSTAIALELLKQGASPNVQDTSGTSPVHDAARTGFLDTLKVLVEHGADVNVP------
               ||  ||  |  |   |  |  | | |   | |    ||
Query    ITNQPEIAEALLGAGCDPELRDFRGNTPLHLACEQGCLASVGVLTQSCTTPHLHSILKAT
         HCCCHHHHHHHHHCCCCCCCCCCCCCCHHHHHHHCCCHHHHHHHHHCCCCCCCCCCCCCC
                 130       140       150       160       170


         100       110       120         130       140       150
         CCCCCCHHHHHHHHCCHHHHHHHHC-CCCCCCC-CCCCCCHHHHHHHCCCHHHHHHHHCC
1bd8A00  DGTGALPIHLAVQEGHTAVVSFLAA-ESDLHRR-DARGLTPLELALQRGAQDLVDILQGH
            |    |||   |    |  |     |        | | | ||      |||  |
Query    NYNGHTCLHLASIHGYLGIVELLVSLGADVNAQEPCNGRTALHLAVDLQNPDLVSLLLKC
         CCCCCHHHHHHHHCCCHHHHHHHHHCCCCCCCCCCCCCHHHHHHHHHCCCHHHHHHHHHC
                 190       200       210       220       230


         C-----------------------------------------------------------
1bd8A00  M-----------------------------------------------------------

Query    GADVNRVTYQGYSPYQLTWGRPSTRIQQQLGQLTLENLQMLPESEDEESYDTESEFTEFT
         CCCCCCCCCCCCCHHHHHHHCCCHHHHHHHHHCCCCCCCCCCCCCCCCCCCCHHCCCCCC
                 250       260       270       280       290


         ------------------
1bd8A00  ------------------

Query    EDELPYDDCVFGGQRLTL
         CCCCCCCCCCCCCCCCCC
                 310
```

```
Percentage Identity = 25.0.

>>> Alignment with 1s70B01:

         ------------------------------------------------------------
1s70B01  ------------------------------------------------------------

Query    MFQAAERPQEWAMEGPRDGLKKERLLDDRHDSGLDSMKDEEYEQMVKELQEIRLEPQEVP
         CCCCCCCHHHHHHCCCCCHHHHHHHHHHHCCCCCCCCCCCHHHHHHHHHHCCCCCCCCCC
                 10        20        30        40        50        60


                                       10        20        30
         ----------------------CCCHHHHHHHHHHHHHHHHCCCCCCCCCCCCCCCCCCC
1s70B01  ----------------------MKMADAKQKRNEQLKRWIGSETDLEPPVVKRKKTKVKF
                                     |    |      |    |
Query    RGSEPWKQQLTEDGDSFLHLAIIH--EEKALTMEVIRQVKGDLAFL---------NFQNN
         CCCCCCCCCCCCCCCCHHHHHHHC--CCHHHHHHHHHHHHHCCCCC---------CCCCC
                 70        80          90       100


                   50        60        70        80        90
         CHHHHHHHHHHHCCHHHHHHHHHHCCCCCCCCCCCCHHHHHHHHCCHHHHHHHHHHCCCC
1s70B01  DDGAVFLAACSSGDTEEVLRLLERGADINYANVDGLTALHQACIDDNVDMVKFLVENGAN
                 |      |    ||  | |       | | || ||       |  |
Query    LQQTPLHLAVITNQPEIAEALLGAGCDPELRDFRGNTPLHLACEQGCLASVGVLTQSCTT
         CCCHHHHHHHHCCHHHHHHHHHHCCCCCCCCCCCCCHHHHHHHHCCHHHHHHHHHHCCCC
                 120       130       140       150       160


                        110       120       130        140
         CCCC------CCCCCHHHHHHHHHCCHHHHHHHHHCCCCCCCC-CCCCCCHHHHC---CC
1s70B01  INQP------DNEGWIPLHAAASCGYLDIAEYLISQGAHVGAV-NSEGDTPLDIA---EE
                      |   || |   ||| | | | | || | |     | | |  |
Query    PHLHSILKATNYNGHTCLHLASIHGYLGIVELLVSLGADVNAQEPCNGRTALHLAVDLQN
         CCCCCCCCCCCCCCHHHHHHHHHCCCHHHHHHHHHCCCCCCCCCCCCCHHHHHHHHHCCC
                 180       190       200       210       220


                  160
         HHHHHHHHHHHCCC----------------------------------------------
1s70B01  EAMEELLQNEVNRQ----------------------------------------------
              ||
Query    PDLVSLLLKCGA-DVNRVTYQGYSPYQLTWGRPSTRIQQQLGQLTLENLQMLPESEDEES
         HHHHHHHHHCCC-CCCCCCCCCCCHHHHHHHCCCHHHHHHHHHCCCCCCCCCCCCCCCCC
                 240        250       260       270       280


         -----------------------------
1s70B01  -----------------------------

Query    YDTESEFTEFTEDELPYDDCVFGGQRLTL
         CCCHHCCCCCCCCCCCCCCCCCCCCCCCC
                  300       310

Percentage Identity = 24.1.

>>> Alignment with 3c5rA00:

         ------------------------------------------------------------
3c5rA00  ------------------------------------------------------------

Query    MFQAAERPQEWAMEGPRDGLKKERLLDDRHDSGLDSMKDEEYEQMVKELQEIRLEPQEVP
         CCCCCCCHHHHHHCCCCCHHHHHHHHHHHCCCCCCCCCCCHHHHHHHHHHCCCCCCCCCC
                 10        20        30        40        50        60


         ------------------------------------------------------------
3c5rA00  ------------------------------------------------------------

Query    RGSEPWKQQLTEDGDSFLHLAIIHEEKALTMEVIRQVKGDLAFLNFQNNLQQTPLHLAVI
         CCCCCCCCCCCCCCCCHHHHHHHCCCHHHHHHHHHHHHHCCCCCCCCCCCCHHHHHHHHH
                 70        80        90       100       110       120


                                  10        20        30
         ---------------C--CCCCCCCHHHHHHHHHCCHHHHHHHHHCCC-------CCCCC
3c5rA00  ---------------P--FTNHRGETLLHIASIKGDIPSVEYLLQNGD-------PNVKD
                               || | || |   |   ||  | |
Query    TNQPEIAEALLGAGCDPELRDFRGNTPLHLACEQGCLASVGVLTQSCTTPHLHSILKATN
         CCCHHHHHHHHHCCCCCCCCCCCCCHHHHHHHHCCHHHHHHHHHHCCCCCCCCCCCCCCC
                130       140       150       160       170       180


           40        50        60         70        80        90
```

```
          CCCCCHHHHHHHCCCHHHHHHHHHHCCCCCC-CCCCCCCCHHHHHHHCCCHHHHHHHHHHCC
3c5rA00   HAGWTPLHEACNHGHLKVVELLLQHKALVN-TTGYQNDSPLHDAAKNGHVDIVKLLLSYG
            | | || |  || |  ||||    | ||          || |      | | |||  |
Query     YNGHTCLHLASIHGYLGIVELLVSLGADVNAQEPCNGRTALHLAVDLQNPDLVSLLLKCG
          CCCCHHHHHHHHHCCCHHHHHHHHHHCCCCCCCCCCCCCHHHHHHHHCCCHHHHHHHHHHCC
                 190       200       210       220       230       240


            100       110       120
          CCCCCCCCCCCCHHHHCCCHHHHHHHCCC-------------------------------
3c5rA00   ASRNAVNIFGLRPVDYTDDESMKSLLLLP-------------------------------
          |  | |   |  |   |      |
Query     ADVNRVTYQGYSPYQLT--WGRPSTRIQQQLGQLTLENLQMLPESEDEESYDTESEFTEF
          CCCCCCCCCCCCHHHHH--HHCCCHHHHHHHHHHCCCCCCCCCCCCCCCCCCCHHCCCCC
                 250         260       270       280       290


          -------------------
3c5rA00   -------------------

Query     TEDELPYDDCVFGGQRLTL
          CCCCCCCCCCCCCCCCCCC
                   310
```

Percentage Identity = 33.9.

>>> Alignment with 1ycsB01:

```
          ------------------------------------------------------------
1ycsB01   ------------------------------------------------------------

Query     MFQAAERPQEWAMEGPRDGLKKERLLDDRHDSGLDSMKDEEYEQMVKELQEIRLEPQEVP
          CCCCCCCCHHHHHCCCCCCHHHHHHHHHHCCCCCCCCCCCCHHHHHHHHHHCCCCCCCCC
                  10        20        30        40        50        60


          -----------------------------------------------------CHHHHHH
1ycsB01   -----------------------------------------------------PLALLLD
                                                               |   |
Query     RGSEPWKQQLTEDGDSFLHLAIIHEEKALTMEVIRQVKGDLAFLNFQNNLQQTP---LHL
          CCCCCCCCCCCCCCCCHHHHHHHCCHHHHHHHHHHHHHHCCCCCCCCCCCCCHH---HHH
                  70        80        90       100       110


           10        20        30        40        50        60
          HHHHCCHHHHHHHHCCCCCCCCCCCCCCCCHHHHHHHHCCHHHHHHHHHHHCCCCC------
1ycsB01   SSLEGEFDLVQRIIYEVDDPSLPNDEGITALHNAVCAGHTEIVKFLVQFGVNVN------
                            || |    | | || |   |    |  | |
Query     AVITNQPEIAEALLGAGCDPELRDFRGNTPLHLACEQGCLASVGVLTQSCTTPHLHSILK
          HHHCCCHHHHHHHHHHCCCCCCCCCCCCCHHHHHHHCCCHHHHHHHHHCCCCCCCCCCCC
          120       130       140       150       160       170


                 70        80        90       100       110       120
          CCCCCCCCHHHHHHHCCCHHHHHHHHHHCCCCCCCCCCCCCCHHHHCCCCCCCCCCCHHHH
1ycsB01   AADSDGWTPLHCAASCNNVQVCKFLVESGAAVFAMTYSDMQTAADKCEEMEEGYTQCSQF
          |    | | || |           ||  || | |       ||
Query     ATNYNGHTCLHLASIHGYLGIVELLVSLGADVNAQEPCNGRTALHLAVDL--QNPDLVSL
          CCCCCCCHHHHHHHHHCCCHHHHHHHHHHCCCCCCCCCCCCCHHHHHHHHHC--CCHHHHHH
          180       190       200       210       220         230


          HHHHHHC-----------------------------------------------------
1ycsB01   LYGVQEK-----------------------------------------------------
          |
Query     LLKCGADVNRVTYQGYSPYQLTWGRPSTRIQQQLGQLTLENLQMLPESEDEESYDTESEF
          HHHCCCCCCCCCCCCCCHHHHHHHCCHHHHHHHHHHCCCCCCCCCCCCCCCCCCCCHHCC
            240       250       260       270       280       290


          ----------------------
1ycsB01   ----------------------

Query     TEFTEDELPYDDCVFGGQRLTL
          CCCCCCCCCCCCCCCCCCCCCC
            300       310
```

Percentage Identity = 22.7.

>>> Alignment with 1bi7B00:

```
          ------------------------------------------------------------
1bi7B00   ------------------------------------------------------------
```

```
Query    MFQAAERPQEWAMEGPRDGLKKERLLDDRHDSGLDSMKDEEYEQMVKELQEIRLEPQEVP
         CCCCCCCHHHHHHCCCCCCHHHHHHHHHHCCCCCCCCCCCHHHHHHHHHHHCCCCCCCCC
                 10        20        30        40        50        60

                                                                 10
         ------------------------------------------------CCCCCHHHHHHH
1bi7B00  ------------------------------------------------EPSADWLATAAA
                                                               |  |
Query    RGSEPWKQQLTEDGDSFLHLAIIHEEKALTMEVIRQVKGDLAFLNFQNNLQQTPLHLAVI
         CCCCCCCCCCCCCCCCHHHHHHHCCCHHHHHHHHHHHHHCCCCCCCCCCCCCHHHHHHHH
                 70        80        90       100       110       120

               20        30        40         50        60
         HCCHHHHHHHHCCCCCCCCCCCCCCCCCCC-CCCCCHHHHHHHHCCCCCCCCCC-----C
1bi7B00  RGRVEEVRALLEAGANPNAPNSYGRRPIQV-MMMGSARVAELLLLHGAEPNCAD-----P
             |   ||| ||  |      |  |       |       |      |
Query    TNQPEIAEALLGAGCDPELRDFRGNTPLHLACEQGCLASVGVLTQSCTTPHLHSILKATN
         CCCHHHHHHHHCCCCCCCCCCCCCCCHHHHHHHCCHHHHHHHHHHCCCCCCCCCCCCCCC
                130       140       150       160       170       180

           70        80        90        100       110       120
         CCCCCHHHHHHHHCCHHHHHHHHHHCCCCCCC-CCCCCCHHHHHHHHCCHHHHHHHCCC-
1bi7B00  ATLTRPVHDAAREGFLDTLVVLHRAGARLDVR-DAWGRLPVDLAEELGHRDVARYLRAA-
                | |   | |     |   ||         ||    ||  |   |    |
Query    YNGHTCLHLASIHGYLGIVELLVSLGADVNAQEPCNGRTALHLAVDLQNPDLVSLLLKCG
         CCCCHHHHHHHCCCHHHHHHHHHHCCCCCCCCCCCCCHHHHHHHHCCHHHHHHHHHHHCC
                190       200       210       220       230       240

         C-----------------------------------------------------------
1bi7B00  A-----------------------------------------------------------
         |
Query    ADVNRVTYQGYSPYQLTWGRPSTRIQQQLGQLTLENLQMLPESEDEESYDTESEFTEFTE
         CCCCCCCCCCCHHHHHHHHCCCHHHHHHHHHCCCCCCCCCCCCCCCCCCCCHHCCCCCCC
                250       260       270       280       290       300

         -----------------
1bi7B00  -----------------

Query    DELPYDDCVFGGQRLTL
         CCCCCCCCCCCCCCCCC
                310

Percentage Identity = 23.2.

>>> Alignment with 1n0qB00:
                                                        10        20
         ---------------------------------------CCCCHHHHHHHCCCHHHHHHH
1n0qB00  ---------------------------------------NGRTPLHLAARNGHLEVVKLL
                                                              ||
Query    MFQAAERPQEWAMEGPRDGLKKERLLDDRHDSGLDSMKDEEYEQMVKELQEIRLEPQEVP
         CCCCCCCHHHHHHCCCCCCHHHHHHHHHHCCCCCCCCCCCHHHHHHHHHHHCCCCCCCCC
                 10        20        30        40        50        60

                30        40            50        60        70
         HHHCCCCCCCCCCCCHHHHHHHHCCC----HHHHHHHHHCCCCCCCCCCCCCHHHHHHHH
1n0qB00  LEAGADVNAKDKNGRTPLHLAARNGH----LEVVKLLLEAGADVNAKDKNGRTPLHLAAR
                      |   ||||          ||        |  |       ||||||
Query    RGSEPWKQQLTEDGDSFLHLAIIHEEKALTMEVIRQVKGDLAFLNFQNNLQQTPLHLAVI
         CCCCCCCCCCCCCCCCHHHHHHHCCCHHHHHHHHHHHHHCCCCCCCCCCCCCHHHHHHHH
                 70        80        90       100       110       120

          80        90
         CCCHHHHHHHHHCCCC-----
1n0qB00  NGHLEVVKLLLEAGAY-----
             |    || ||
Query    TNQPEIAEALLGAGCDPELRD
         CCCHHHHHHHHCCCCCCCCCC
                130       140

Percentage Identity = 23.7.

>>> Alignment with 1s70B02:

         ------------------------------------------------------------
1s70B02  ------------------------------------------------------------

Query    MFQAAERPQEWAMEGPRDGLKKERLLDDRHDSGLDSMKDEEYEQMVKELQEIRLEPQEVP
```

```
         CCCCCCCCHHHHHCCCCCCHHHHHHHHHHCCCCCCCCCCCCHHHHHHHHHHCCCCCCCCC
                 10        20        30        40        50        60


         ---------------------------------------------------CCCHHHHHH
1s70B02  ---------------------------------------------------GVDIEAARK
                                                                  |
Query    RGSEPWKQQLTEDGDSFLHLAIIHEEKALTMEVIRQVKGDLAFLNFQNNLQQTPLHLAVI
         CCCCCCCCCCCCCCCCHHHHHHHCCHHHHHHHHHHHHHHCCCCCCCCCCCCCHHHHHHHH
                 70        80        90       100       110       120

                  20        30        40        50        60
         HHHHHHHHHHHHHHHHCCCCCCCCCCCCCHHHHHHHHCCHHHHHHHHHCCCCCCC-----
1s70B02  EEERIMLRDARQWLNSGHINDVRHAKSGGTALHVAAAKGYTEVLKLLIQARYDVN-----
                                    | | || |   |       | |
Query    TNQPEIAEALLGAGCDPEL----RDFRGNTPLHLACEQGCLASVGVLTQSCTTPHLHSIL
         CCCHHHHHHHHHCCCCCCC----CCCCCCHHHHHHHHCCHHHHHHHHHCCCCCCCCCCCC
                130           140       150       160       170

              70        80        90       100       110       120
         -CCCCCCCCHHHHHHHCCCHHHHHHHHHCCCCCCCC-CCCCCCCCCCCCHHHHHHHHHHH
1s70B02  -IKDYDGWTPLHAAAHWGKEEACRILVENLCDMEAV-NKVGQTAFDVADEDILGYLEELQ
             | | | || |   |       ||    |  |     | ||   |       |  |
Query    KATNYNGHTCLHLASIHGYLGIVELLVSLGADVNAQEPCNGRTALHLAVDLQNPDLVSLL
         CCCCCCCCHHHHHHHHCCCHHHHHHHHHCCCCCCCCCCCCCHHHHHHHHCCHHHHHHHHH
          180       190       200       210       220       230


         HHCCCCC-----------------------------------------------------
1s70B02  KKQNLLH-----------------------------------------------------
          |
Query    LKCGADVNRVTYQGYSPYQLTWGRPSTRIQQQLGQLTLENLQMLPESEDEESYDTESEFT
         HHCCCCCCCCCCCCCHHHHHHHCCCHHHHHHHHHCCCCCCCCCCCCCCCCCCCCCHHCCC
          240       250       260       270       280       290


         ---------------------
1s70B02  ---------------------

Query    EFTEDELPYDDCVFGGQRLTL
         CCCCCCCCCCCCCCCCCCCCC
          300       310

Percentage Identity = 20.9.

>>> Alignment with 1sw6A00:
                                          10                     20
         --------------CCEEECC-----------CCCCCCCCCCC-------------CCCE
1sw6A00  --------------GPIITFT-----------HDLTSDFLSSP-------------LKIM
                       ||                ||   |                  |
Query    MFQAAERPQEWAMEGP---RDGLKKERLLDDRHDSGLDSMKDEEYEQMVKELQEIRLEPQ
         CCCCCCCCHHHHHCCC---CCCHHHHHHHHHHCCCCCCCCCCCCHHHHHHHHHHCCCCCC
                 10           20        30        40        50

               30        40          50        60        70        80
         ECCCCCCCCCHHHHHHHHHHHHHHHCC--HHHHHHHHHHHCCCCCCCCCCCCCCCCCCHH
1sw6A00  KALPSPVVNDNEQKMKLEAFLQRLLFS--FDSLLQEVNDAFPNTQLNLNIPVDEHGNTPL
                            ||                          ||        |||
Query    EVPRGSEPWKQQLTEDGDSFLHLAIIHEEKALTMEVIRQVKGDL-AFLNFQ-NNLQQTPL
         CCCCCCCCCCCCCCCCCCCHHHHHHHCCHHHHHHHHHHHHHHCC-CCCCCC-CCCCCHHH
          60        70        80        90       100         110

                 90       100       110       120       130       140
         HHHHHCCCHHHHHHHHHCCCCCCCCCCCCCHHHHHHHHCCHHHHCCHHHHHHHHHHHHHH
1sw6A00  HWLTSIANLELVKHLVKHGSNRLYGDNMGESCLVKAVKSVNNYDSGTFEALLDYLYPCLI
         |        |    |   |      |  |   |  |         |             |
Query    HLAVITNQPEIAEALLGAGCDPELRDFRGNTPLHLACEQGCLASVGVLTQSCTTPHLHSI
         HHHHHCCCHHHHHHHHHCCCCCCCCCCCCCHHHHHHHHCCHHHHHHHHHCCCCCCCCCCC
           120       130       140       150       160       170

                150       160       170       180            190
         EECCCCCCHHHHHHHHHCCCCCHHHHHHHHHHHHHHHHHHHHCCEEECC----HHHHHCC
1sw6A00  LEDSMNRTILHHIIITSGMTGCSAAAKYYLDILMGWIVKKQNRPIQSGD----SILENLD
         |                                              |       |  |
Query    LKATNYNGHTCLHLASIH-----------------------------GYLGIVELLVSLG
         CCCCCCCCCHHHHHHHHC-----------------------------CCHHHHHHHHHCC
           180       190                                    200

          200       210       220       230       240       250
         HHHHHHHCCCCCCCCCCHHHHHHHHHCCHHHHHHHHHCCCCCCCCCCCCCCHHHHCCC--
```

```
1sw6A00  LKWIIANMLNAQDSNGDTCLNIAARLGNISIVDALLDYGADPFIANKSGLRPVDFGAG--
              |        || | |  |  | |   |  ||  |||       |  |     |
Query    -----ADVNAQEPCNGRTALHLAVDLQNPDLVSLLLKCGADVNRVTYQGYSPYQLTWGRP
         -----CCCCCCCCCCCCHHHHHHHHCCCHHHHHHHHHCCCCCCCCCCCCCCHHHHHHHCC
               210       220       230       240       250       260


         --------------------------------------------------------
1sw6A00  --------------------------------------------------------

Query    STRIQQQLGQLTLENLQMLPESEDEESYDTESEFTEFTEDELPYDDCVFGGQRLTL
         CHHHHHHHHHCCCCCCCCCCCCCCCCCCCCHHCCCCCCCCCCCCCCCCCCCCCCCC
               270       280       290       300       310

Percentage Identity = 17.3.

>>> Alignment with 3c5rA00:
                                                   10        20
         ----------------------------------CCCCCCCCCHHHHHHHHHCCHHHHHH
3c5rA00  ----------------------------------PFTNHRGETLLHIASIKGDIPSVEYL

Query    MFQAAERPQEWAMEGPRDGLKKERLLDDRHDSGLDSMKDEEYEQMVKELQEIRLE-PQEV
         CCCCCCCCHHHHHCCCCCCHHHHHHHHHHCCCCCCCCCCCHHHHHHHHHHHHCCC-CCCC
                 10        20        30        40        50


           30         40        50            60        70        80
         HHCCCCCCC--CCCCCCHHHHHHHCCC----HHHHHHHHHCCCCCCCCCCCCCCHHHHHH
3c5rA00  LQNGDPNVK--DHAGWTPLHEACNHGH----LKVVELLLQHKALVNTTGYQNDSPLHDAA
              |        |   || |  |        |        |  |        ||| |
Query    PRGSEPWKQQLTEDGDSFLHLAIIHEEKALTMEVIRQVKGDLAFLNFQNNLQQTPLHLAV
         CCCCCCCCCCCCCCCCCHHHHHHHHCCCHHHHHHHHHHHHHHCCCCCCCCCCCHHHHHHH
                  70        80        90       100       110


                 90       100       110       120
         HCCCHHHHHHHHHCCCCCCCCCCCCCHHHHCCCHHHHHHHHCCC
3c5rA00  KNGHVDIVKLLLSYGASRNAVNIFGLRPVDYTDDESMKSLLLLP
               |   ||  |
Query    ITNQPEIAEALLGAGCD---------------------------
         HCCCHHHHHHHHHCCCC---------------------------
                 130

Percentage Identity = 13.7.

>>> Alignment with 1n0rA00:
                    10         20         30        40        50
         ---CCCCHHHHHHHHCC--HHHHHHHHHHC-CCCCCCCCCCCCHHHHHHHHCCHHHHHHH
1n0rA00  ---NGRTPLHLAARNGH--LEVVKLLLEAG-ADVNAKDKNGRTPLHLAARNGHLEVVKLL
                |   |       |    ||                           ||
Query    MFQAAERPQEWAMEGPRDGLKKERLLDDRHDSGLDSMKDEEYEQMVKELQEIRLEPQEVP
         CCCCCCCCHHHHHCCCCCCHHHHHHHHHHCCCCCCCCCCCHHHHHHHHHHHHCCCCCCCC
                 10        20        30        40        50        60


             60        70        80            90       100       110
         HHCCCCCCCCCCCCCHHHHHHHHCCC----HHHHHHHHHCCCCCCCCCCCCCCHHHHHHH
1n0rA00  LEAGADVNAKDKNGRTPLHLAARNGH----LEVVKLLLEAGADVNAKDKNGRTPLHLAAR
                      |   ||||          ||        |  |
Query    RGSEPWKQQLTEDGDSFLHLAIIHEEKALTMEVIRQVKGDLAFLNFQN------------
         CCCCCCCCCCCCCCCCHHHHHHHHCCCHHHHHHHHHHHHHHCCCCCCC------------
                  70        80        90       100


                120
         CCCHHHHHHHHHHCCC
1n0rA00  NGHLEVVKLLLEAGAY

Query    ----------------
         ----------------


Percentage Identity = 12.7.

>>> Alignment with 2bkgA00:
                 10        20        30        40           50
         CHHHHHHHHHHHHCHHHHHHHHHHHCCCCCCCCCCCCHHHHHHHHHCC---HHHHHHHHH
2bkgA00  SDLGKKLLEAARAGQDDEVRILMANGADVNAEDTYGDTPLHLAARVGH---LEIVEVLLK
                                               |   |
Query    -------------------------------MFQAAERPQEWAMEGPRDGLKKERLLDDR
         -------------------------------CCCCCCCCHHHHHCCCCCCHHHHHHHHHH
                                                10        20
```

```
          60        70        80        90       100       110
         CCCCCCCCCCCCCCHHHHHHHHCCCHHHHHHHHHHCCCCCCCCCCCCCCHHHHHHHHCCC---
2bkgA00  NGADVNALDFSGSTPLHLAAKRGHLEIVEVLLKYGADVNADDTIGSTPLHLAADTGH---
                                 ||  ||              |   ||||
Query    HDSGLDSMKDEEYEQMVKELQEIRLEPQEVPRGSEPWKQQLTEDGDSFLHLAIIHEEKAL
         CCCCCCCCCCCHHHHHHHHHHHCCCCCCCCCCCCCCCCCCCCCCCCHHHHHHHHCCCHHH
                  40        50        60        70        80

             120       130       140       150
         -HHHHHHHHHCCCCCCCCCCCCCCHHHHHHHCCCCCHHHHHCC
2bkgA00  -LEIVEVLLKYGADVNAQDKFGKTAFDISIDNGN-DLAEIL-
           |         |  | |
Query    TMEVIRQVKGDLAFLNFQNNLQQ-------------------
         HHHHHHHHHHCCCCCCCCCCCCC-------------------
                 100       110

Percentage Identity = 9.7.

>>> Alignment with 1s70B02:
                      10        20        30        40        50
         -----CCCHHHHHHHHHHHHHHHHHHHHHHHHCCCCCCCCCCCCCHHHHHHHHCCHHHHHH
1s70B02  -----GVDIEAARKEEERIMLRDARQWLNSGHINDVRHAKSGGTALHVAAAKGYTEVLKL
                  | |  |  |  |   |                              |
Query    MFQAAERPQEWA-MEGPRDGLKKERLLDDRHDSGLDSMKDEEYEQMVKELQEIRLEPQEV
         CCCCCCCCHHHH-HCCCCCCHHHHHHHHHHCCCCCCCCCCCHHHHHHHHHHCCCCCCCC
                 10          20        30        40        50

            60        70        80            90       100       110
         HHCCCCCCCCCCCCCCCCHHHHHHHCCC----HHHHHHHHHCCCCCCCCCCCCCCCCCCCC
1s70B02  LIQARYDVNIKDYDGWTPLHAAAHWGK----EEACRILVENLCDMEAVNKVGQTAFDVAD
                      ||   || |          |  |     |      |   |
Query    PRGSEPWKQQLTEDGDSFLHLAIIHEEKALTMEVIRQVKGDLAFLNFQNNLQQ-------
         CCCCCCCCCCCCCCCCCHHHHHHHHCCHHHHHHHHHHHHHHCCCCCCCCCCCC-------
                  70        80        90       100       110

               120
         CHHHHHHHHHHHHHCCCCC
1s70B02  EDILGYLEELQKKQNLLH

Query    ------------------
         ------------------

Percentage Identity = 13.2.

>>> Alignment with 1ycsB01:
                        10          20          30        40
         -------CHHHHHHHHHHCCHHHHH--HHC--CCCCCCCCCCCCCCCHHHHHHHHCCHHH
1ycsB01  -------PLALLLDSSLEGEFDLVQ--RII--YEVDDPSLPNDEGITALHNAVCAGHTEI
              |           ||  |      |              ||                |
Query    MFQAAERP----QEWAMEGPRDGLKKERLLDDRHDSGLDSMKDEEYEQMVKELQEIRLEP
         CCCCCCCC----HHHHHCCCCCCHHHHHHHHHHCCCCCCCCCCCHHHHHHHHHHHCCCCC
                     10        20        30        40        50

                  60        70           80        90       100
         HHHHHHHCCCCCCCCCCCCCHHHHHHHHCCC----HHHHHHHHHCCCCCCCCCCCCCCCHH
1ycsB01  VKFLVQFGVNVNAADSDGWTPLHCAASCNN----VQVCKFLVESGAAVFAMTYSDMQTAA
                         ||   || |           |        |          ||
Query    QEVPRGSEPWKQQLTEDGDSFLHLAIIHEEKALTMEVIRQVKGDLAFLNFQNN-LQQTP-
         CCCCCCCCCCCCCCCCCCCCHHHHHHHHCCCHHHHHHHHHHHHHHCCCCCCCCC-CCCHH-
           60        70        80        90       100        110

           110       120
         HHCCCCCCCCCCHHHHHHHHHHC
1ycsB01  DKCEEMEEGYTQCSQFLYGVQEK

Query    -----------------------
         -----------------------

Percentage Identity = 13.3.

>>> Alignment with 1awcB00:
                 10        20        30           40           50
         CHHHHHHHHHHHHCCHHHHHHHHHHHCCCCCCCCC---CCCHHHHHHHHHCC---HHHHHHHH
1awcB00  DLGKKLLEAARAGQDDEVRILMANGAPFTTDWL---GTSPLHLAAQYGH---FSTTEVLL
                                                |   |
Query    -------------------------------MFQAAERPQEWAMEGPRDGLKKERLLDD
         -------------------------------CCCCCCCCHHHHHCCCCCCHHHHHHHHH
                                                 10        20
```

```
             60        70        80        90       100       110
         CCCCCCCCCCCCCCCHHHHHHHHHCCHHHHHHHHCCCCCCCCCCCCCCHHHHHHHHCCC--
1awcB00  RAGVSRDARTKVDRTPLHMAASEGHANIVEVLLKHGADVNAKDMLKMTALHWATEHNH--
         |     |               |      ||                  || |  |
Query    RHDSGLDSMKDEEYEQMVKELQEIRLEPQEVPRGSEPWKQQLTEDGDSFLHLAIIHEEKA
         HCCCCCCCCCCCHHHHHHHHHHHCCCCCCCCCCCCCCCCCCCCCCCCCHHHHHHHHCCCHH
                   40        50        60        70        80


                120       130       140       150
         --HHHHHHHHHCCCCCCCCCCCCCCHHHHHHHHCCCHHHHHHC
1awcB00  --QEVVELLIKYGADVHTQSKFCKTAFDISIDNGNEDLAEILQ
            ||        |    |
Query    LTMEVIRQVKGDLAFLNFQN-----------------------
         HHHHHHHHHHHCCCCCCCCC-----------------------
                  100

Percentage Identity = 9.8.

>>> Alignment with 1ympA00:
                 10        20        30        40        50
         CCCCCCCCCCCCCCHHHHHHHHCCCCHHHHHHHCCCCCCCCCCCCCCHHHHHHHCCC---
1ympA00  RATDLDARMHDGTTPLILAARLALEGMLEDLINSHADVNAVDDLGKSALHWAAAVNN---
                                                           ||
Query    ----------------------------------------MFQAAERPQEWAMEGPRDGL
         ----------------------------------------CCCCCCCCHHHHHCCCCCCH
                                                         10        20


          60        70        80        90       100       110
         HHHHHHHHHCCCCCCCCCCCCCHHHHHHHHHCCHHHHHHHHHCCCCCCCCCCCCCCHHHH
1ympA00  VDAAVVLLKNGANKDMQNNKEETPLFLAAREGSYETAKVLLDHFANRDITDHMDRLPRDI
                       |     |         |   |   |
Query    KKERLLDDRHDSGLDSMKDEEYEQMVKELQEIRLEPQEVPRGSEPWKQQLTEDGDSFLHL
         HHHHHHHHHCCCCCCCCCCCCHHHHHHHHHHCCCCCCCCCCCCCCCCCCCCCCCCCHHHH
                 30        40        50        60        70        80


         120           130
         HHHCCC----HHHHHHHC--
1ympA00  AQERMH----HDIVRLLD--
         |             |
Query    AIIHEEKALTMEVIRQVKGD
         HHHCCCHHHHHHHHHHHHHC
                 90       100

Percentage Identity = 6.9.

>>> Alignment with 1s70B01:
                 10        20        30        40        50
         CCCHHHHHHHHHHHHHHHHCCCCCCCCCCCCCCCCCCCHHHHHHHHHHHHCC--HHHHHH
1s70B01  MKMADAKQKRNEQLKRWIGSETDLEPPVVKRKKTKVKFDDGAVFLAACSSGD--TEEVLR
                                                       |
Query    ---------------------------MFQAAER--------PQEWAMEGPRDGLKKERL
         ---------------------------CCCCCCC--------CHHHHHCCCCCCHHHHHH
                                                    10        20


                    70        80        90       100       110
         HHHHC-CCCCCCCCCCCHHHHHHHCCCHHHHHHHHHHCCCCCCCCCCCCCHHHHHHHHHC
1s70B01  LLERG-ADINYANVDGLTALHQACIDDNVDMVKFLVENGANINQPDNEGWIPLHAAASCG
         |  |                                       |    |   || |
Query    LDDRHDSGLDSMKDEEYEQMVKELQEIRLEPQEVPRGSEPWKQQLTEDGDSFLHLAII--
         HHHHCCCCCCCCCCCHHHHHHHHHHHCCCCCCCCCCCCCCCCCCCCCCCCHHHHHHHH--
            30        40        50        60        70        80


         120       130       140       150       160
         CHHHHHHHHHCCCCCCCCCCCCCHHHHCCCHHHHHHHHHHHHCCC
1s70B01  YLDIAEYLISQGAHVGAVNSEGDTPLDIAEEEAMEELLQNEVNRQ

Query    ---------------------------------------------
         ---------------------------------------------


Percentage Identity = 4.9.

>>> Alignment with 2pw8I00:

         ------------------------------------------------------------
2pw8I00  ------------------------------------------------------------

Query    MFQAAERPQEWAMEGPRDGLKKERLLDDRHDSGLDSMKDEEYEQMVKELQEIRLEPQEVP
```

```
         CCCCCCCCHHHHHCCCCCCHHHHHHHHHHHCCCCCCCCCCCCHHHHHHHHHHHCCCCCCCC
                 10        20        30        40        50        60


         ------------------------------------------------------------
2pw8I00  ------------------------------------------------------------

Query    RGSEPWKQQLTEDGDSFLHLAIIHEEKALTMEVIRQVKGDLAFLNFQNNLQQTPLHLAVI
         CCCCCCCCCCCCCCCCHHHHHHHCCCHHHHHHHHHHHHHHCCCCCCCCCCCCCHHHHHHHH
                 70        80        90       100       110       120


         ------------------------------------------------------------
2pw8I00  ------------------------------------------------------------

Query    TNQPEIAEALLGAGCDPELRDFRGNTPLHLACEQGCLASVGVLTQSCTTPHLHSILKATN
         CCCHHHHHHHHHCCCCCCCCCCCCCCHHHHHHHCCCHHHHHHHHHCCCCCCCCCCCCCC
                130       140       150       160       170       180


         ------------------------------------------------------------
2pw8I00  ------------------------------------------------------------

Query    YNGHTCLHLASIHGYLGIVELLVSLGADVNAQEPCNGRTALHLAVDLQNPDLVSLLLKCG
         CCCCHHHHHHHHCCCHHHHHHHHHCCCCCCCCCCCCCHHHHHHHHCCCHHHHHHHHHCC
                190       200       210       220       230       240


         ------------------------------------------------------------
2pw8I00  ------------------------------------------------------------

Query    ADVNRVTYQGYSPYQLTWGRPSTRIQQQLGQLTLENLQMLPESEDEESYDTESEFTEFTE
         CCCCCCCCCCCCHHHHHHCCCHHHHHHHHHCCCCCCCCCCCCCCCCCCCCHHCCCCCCC
                250       260       270       280       290       300

                   10        20        30        40        50
         --CCCCCCCCCCEECCCCCCCCCCCCCCEEEECCCCCCCCEEEECCCEECCCCCCCCCCCC
2pw8I00  --LTYTDCTESGQNLCLCEGSN-CGQGNKCILG-DGEKNQCVTGEGTPKPQSGDFEEIP-
           | | ||   || | |
Query    DELPYDDCVFGGQRLTL-------------------------------------------
         CCCCCCCCCCCCCCCCC-------------------------------------------
                310


         CCC
2pw8I00  ELQ

Query    ---
         ---


Percentage Identity = 13.1.

>>> Alignment with 1bi7B00:
         ------------------------------------------------------------
1bi7B00  ------------------------------------------------------------

Query    MFQAAERPQEWAMEGPRDGLKKERLLDDRHDSGLDSMKDEEYEQMVKELQEIRLEPQEVP
         CCCCCCCCHHHHHCCCCCCHHHHHHHHHHHCCCCCCCCCCCCHHHHHHHHHHHCCCCCCCC
                 10        20        30        40        50        60

                           10            20        30        40
         -----------CCCCCHHHHHHHHCC----HHHHHHHHCCCCCCCCCCCCCCCCCCCCC
1bi7B00  -----------EPSADWLATAAARGR----VEEVRALLEAGANPNAPNSYGRRPIQVMMM
                    |     |  |          |  |      |  |  |
Query    RGSEPWKQQLTEDGDSFLHLAIIHEEKALTMEVIRQVKGDLAFLNFQNN-----------
         CCCCCCCCCCCCCCCCHHHHHHHCCCHHHHHHHHHHHHHHCCCCCCCCC-----------
                 70        80        90       100

            50        60        70        80        90       100
         CCHHHHHHHHCCCCCCCCCCCCCCCCHHHHHHHHCCHHHHHHHHHHCCCCCCCCCCCCH
1bi7B00  GSARVAELLLLHGAEPNCADPATLTRPVHDAAREGFLDTLVVLHRAGARLDVRDAWGRLP

Query    ------------------------------------------------------------
         ------------------------------------------------------------


           110       120
         HHHHHHHCCHHHHHHHCCCC
```

```
1bi7B00   VDLAEELGHRDVARYLRAAA

Query     --------------------
          --------------------


Percentage Identity = 6.4.

>>> Alignment with 1gupA02:

          ------------------------------------------------------------
1gupA02   ------------------------------------------------------------

Query     MFQAAERPQEWAMEGPRDGLKKERLLDDRHDSGLDSMKDEEYEQMVKELQEIRLEPQEVP
          CCCCCCCCHHHHHCCCCCCHHHHHHHHHHCCCCCCCCCCCCHHHHHHHHHHCCCCCCCCC
                  10        20        30        40        50        60


          ------------------------------------------------------------
1gupA02   ------------------------------------------------------------

Query     RGSEPWKQQLTEDGDSFLHLAIIHEEKALTMEVIRQVKGDLAFLNFQNNLQQTPLHLAVI
          CCCCCCCCCCCCCCCCHHHHHHHCCCHHHHHHHHHHHHHHCCCCCCCCCCCCCHHHHHHHH
                  70        80        90       100       110       120


          ------------------------------------------------------------
1gupA02   ------------------------------------------------------------

Query     TNQPEIAEALLGAGCDPELRDFRGNTPLHLACEQGCLASVGVLTQSCTTPHLHSILKATN
          CCCHHHHHHHHHCCCCCCCCCCCCCHHHHHHHCCCHHHHHHHHHCCCCCCCCCCCCCC
                 130       140       150       160       170       180


          ------------------------------------------------------------
1gupA02   ------------------------------------------------------------

Query     YNGHTCLHLASIHGYLGIVELLVSLGADVNAQEPCNGRTALHLAVDLQNPDLVSLLLKCG
          CCCCHHHHHHHHHCCCHHHHHHHHHCCCCCCCCCCCCCHHHHHHHHCCCHHHHHHHHHCC
                 190       200       210       220       230       240


          ------------------------------------------------------------
1gupA02   ------------------------------------------------------------

Query     ADVNRVTYQGYSPYQLTWGRPSTRIQQQLGQLTLENLQMLPESEDEESYDTESEFTEFTE
          CCCCCCCCCCCCHHHHHHCCHHHHHHHHHCCCCCCCCCCCCCCCCCCCHHCCCCCCC
                 250       260       270       280       290       300

                   10        20        30        40        50
          -CCCCCCCCCCCCCCCCCCCCCCCCCCCCEEEECCCCCCCCCCCCCCCCCCCCCCCCC
1gupA02   -VLPAHDPDCFLCAGNVRVTGDKNPDYTGTYVFTNDFAALMSDTPDAPESHDPLMRCQSA
            ||  | ||    |  | |
Query     DELP-YD-DCVF--GGQRLTL---------------------------------------
          CCCC-CC-CCCC--CCCCCCC---------------------------------------
                   310

                   70        80        90       100       110
          CEEEEEEECCCCCCCCCCCCCHHHHHHHHHHHHHHHHHHHHHHCCEEEEECCCCCCCCCCC
1gupA02   RGTSRVICFSPDHSKTLPELSVAALTEIVKTWQEQTAELGKTYPWVQVFENKGAAMGCSN

Query     ------------------------------------------------------------
          ------------------------------------------------------------


                  130
          CCCEEEEEEEECCC
1gupA02   PHPGGQIWANSFL

Query     -------------
          -------------


Percentage Identity = 6.1.

>>> Alignment with 1wdyA00:
                  10        20        30        40        50        60
          CHHHHHHHHHHHHHHCCCHHHHHHHHHCCCCCCCCCCCCCCHHHHHHHCCCHHHHHHHHH
1wdyA00   AAVEDNHLLIKAVQNEDVDLVQQLLEGGANVNFQEEEGGWTPLHNAVQMSREDIVELLLR
```

```
Query    ------------------------------------------------------------
         ------------------------------------------------------------


                 70        80          90        100       110
         CCCCCCCCCCCCCCHHHHHHHHCC--HHHHHHHHHC-CCCCCCCCCCCCHHHHHHHHCCC
1wdyA00  HGADPVLRKKNGATPFLLAAIAGS--VKLLKLFLSK-GADVNECDFYGFTAFMEAAVYGK
                       |   |        |   |
Query    -----MFQA--AERPQEWAMEGPRDGLKKERLLDDRHDSGLDSMKDEEYEQMVKELQEIR
         -----CCCC--CCCCHHHHHCCCCCCHHHHHHHHHHCCCCCCCCCCCHHHHHHHHHHHCC
                        10        20        30        40        50

         120       130       140       150       160       170
         HHHHHHHHHCCCCCCCCCCCCHHHHHCCCCCCHHHHHHHHHCCHHHHHHHHHCCCCCCCC
1wdyA00  VKALKFLYKRGANVNLRRKTKEDQERLRKGGATALMDAAEKGHVEVLKILLDEMGADVNA

Query    LEPQEVPRGSEPWKQQLTED----------------------------------------
         CCCCCCCCCCCCCCCCCCCC----------------------------------------
              60        70

         180       190       200       210       220       230
         CCCCCCCHHHHHHHCCCCCCHHHHHHHHHHCCCCCCCCCCCCCHHHHHHHHCCCHHHHHH
1wdyA00  CDNMGRNALIHALLSSDDSDVEAITHLLLDHGADVNVRGERGKTPLILAVEKKHLGLVQR

Query    ------------------------------------------------------------
         ------------------------------------------------------------


         240       250       260       270       280
         HHHCCCCCCCCCCCCCCCHHHHHHHCCCHHHHHHHHHHCCCCCCCCCC
1wdyA00  LLEQEHIEINDTDSDGKTALLLAVELKLKKIAELLCKRGASTDCGDLV

Query    ------------------------------------------------
         ------------------------------------------------


Percentage Identity = 1.4.

>>> Alignment with 1ubdC04:

         ------------------------------------------------------------
1ubdC04  ------------------------------------------------------------

Query    MFQAAERPQEWAMEGPRDGLKKERLLDDRHDSGLDSMKDEEYEQMVKELQEIRLEPQEVP
         CCCCCCCCHHHHHCCCCCCHHHHHHHHHHCCCCCCCCCCCHHHHHHHHHHHCCCCCCCCC
                 10        20        30        40        50        60


         ------------------------------------------------------------
1ubdC04  ------------------------------------------------------------

Query    RGSEPWKQQLTEDGDSFLHLAIIHEEKALTMEVIRQVKGDLAFLNFQNNLQQTPLHLAVI
         CCCCCCCCCCCCCCCHHHHHHHHCCHHHHHHHHHHHHHHHCCCCCCCCCCCCHHHHHHHH
                 70        80        90       100       110       120


         ------------------------------------------------------------
1ubdC04  ------------------------------------------------------------

Query    TNQPEIAEALLGAGCDPELRDFRGNTPLHLACEQGCLASVGVLTQSCTTPHLHSILKATN
         CCCHHHHHHHHHCCCCCCCCCCCCCCHHHHHHHCCHHHHHHHHHHCCCCCCCCCCCCCCC
                130       140       150       160       170       180


         ------------------------------------------------------------
1ubdC04  ------------------------------------------------------------

Query    YNGHTCLHLASIHGYLGIVELLVSLGADVNAQEPCNGRTALHLAVDLQNPDLVSLLLKCG
         CCCCHHHHHHHHCCCHHHHHHHHHCCCCCCCCCCCCCHHHHHHHHHCCCHHHHHHHHHCC
                190       200       210       220       230       240


         ------------------------------------------------------------
1ubdC04  ------------------------------------------------------------

Query    ADVNRVTYQGYSPYQLTWGRPSTRIQQQLGQLTLENLQMLPESEDEESYDTESEFTEFTE
         CCCCCCCCCCCCHHHHHHHCCCHHHHHHHHHCCCCCCCCCCCCCCCCCCCHHCCCCCCCC
                250       260       270       280       290       300


                   10        20
```

```
          -CCCCC-CCCCCCCCCCCCCCCCHHHHHHHC
1ubdC04   -DRPYV-CPFDGCNKKFAQSTNLKSHILTHA
             ||   | | |       |    |
Query     DELPYDDCVFGG------QRLTL--------
          CCCCCCCCCCCC------CCCCC--------
                 310

Percentage Identity = 24.1.
```

Supplementary Figure 3. pDomThreader prediction results identifying 46 possible structural templates with reliable secondary structural similarity.

A

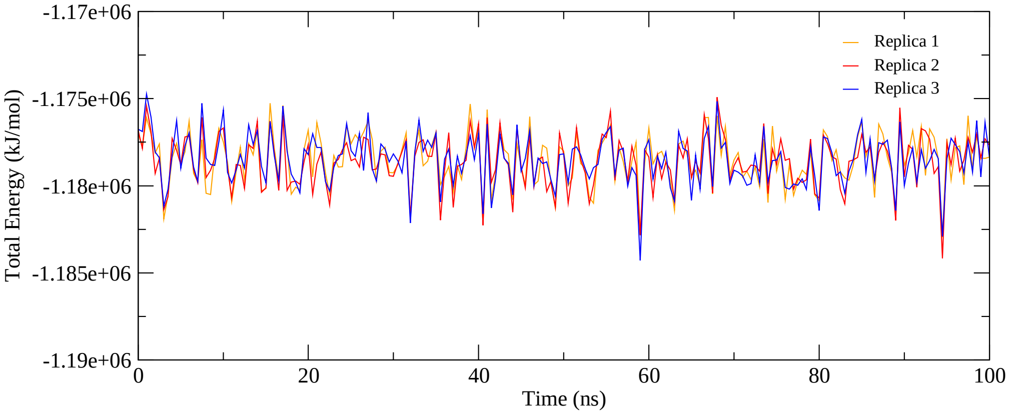

B

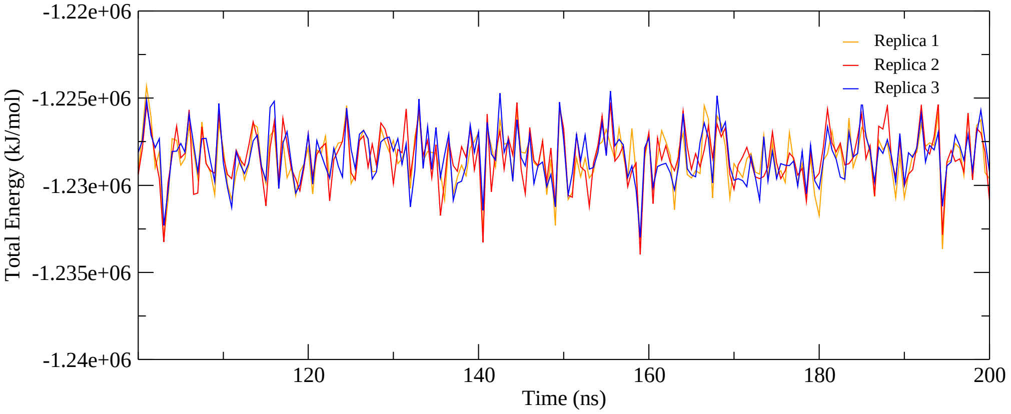

Supplementary Figure 4. The total energy variation of the three system replicas for the initial (A) and final (B) 100ns simulation.

A

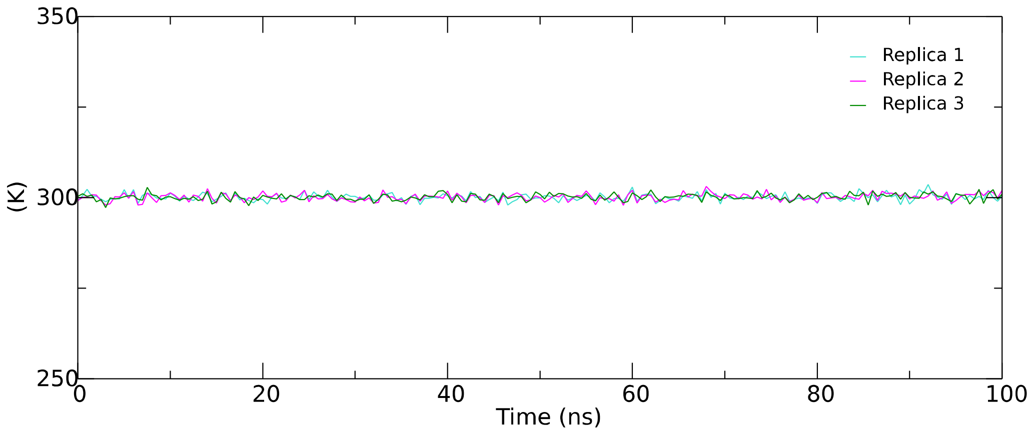

B

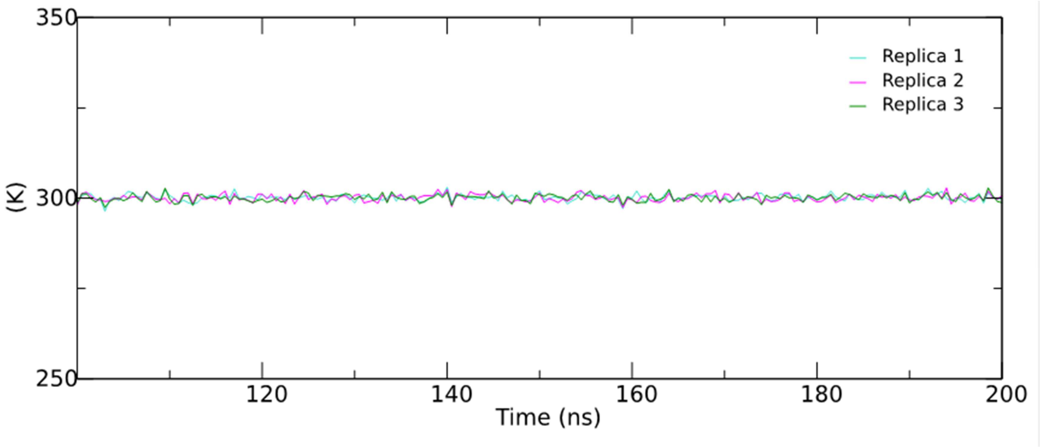

Supplementary Figure 5. Temperature of the three system replicas for the initial 100 ns (A) and the final 100ns (B).

A

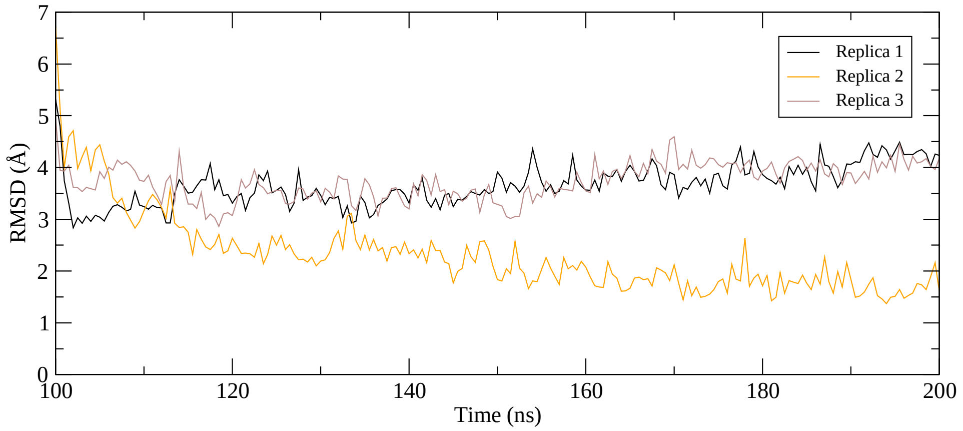

B

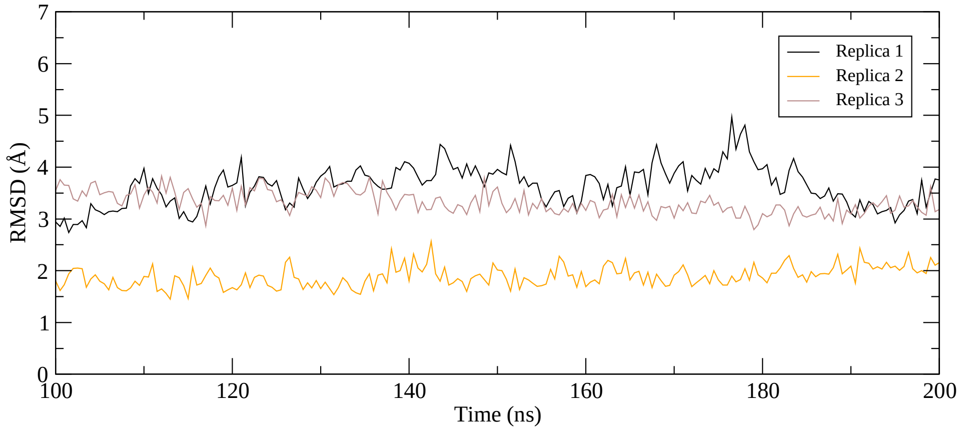

Supplementary Figure 6. RMSD of the complexed IκBα/NF-κB protein backbone against the average structure of the three system replicas for the initial 100 ns (A) and the final 100 ns (B).

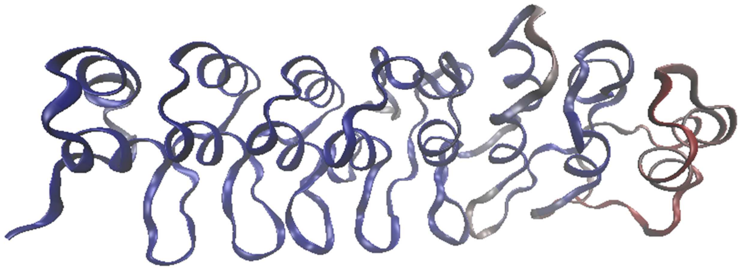

(RMSF after 100ns)

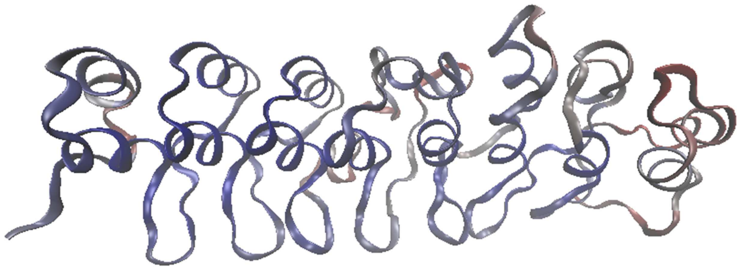

(RMSF after 200ns)

Supplementary Figure 7. Root mean square fluctuations of amino acid residues mapped onto Cα-backbone atoms of IκBα (from blue to red) after 100 ns (top) and 200 ns (bottom).

A

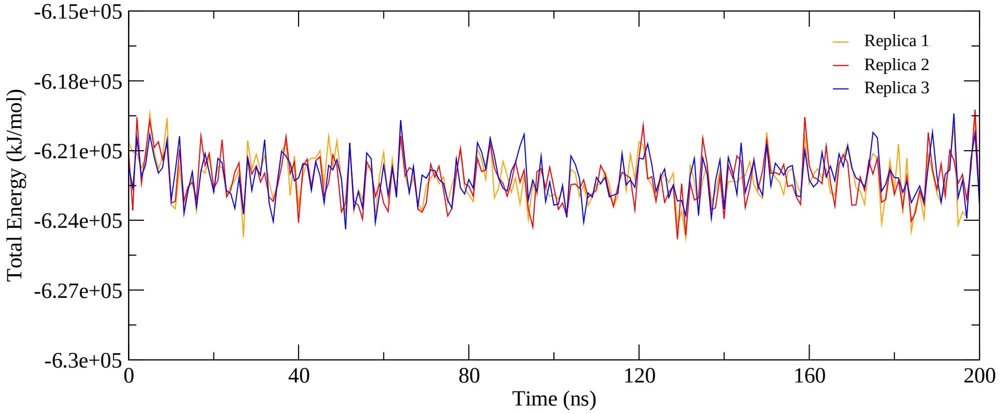

B

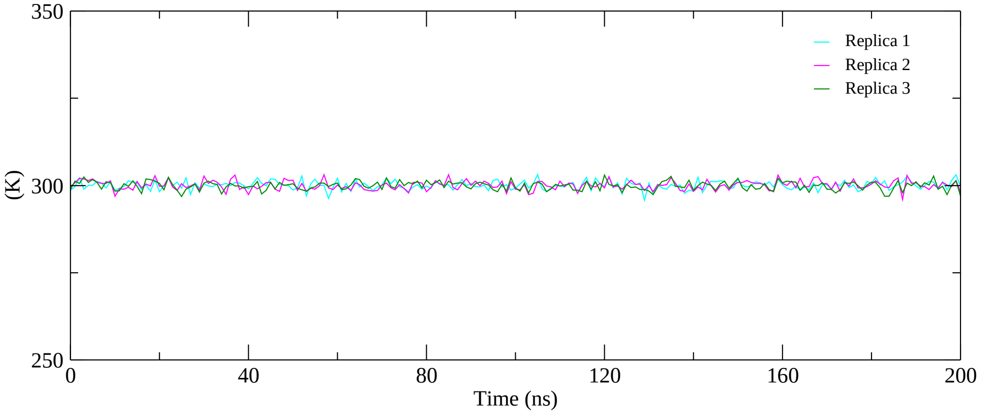

Supplementary Figure 8. (A) The total energy variation of the three system replicas for the free IκBα simulations. (B) Temperature of the three system replicas for the free IκBα simulations.
